# Ferroptosis in cardiovascular diseases: molecular mechanisms and a novel therapeutic target

**DOI:** 10.1186/s43556-026-00420-9

**Published:** 2026-03-08

**Authors:** Suli Yu, Zhen Pang, Hong Fang, Chi Liu

**Affiliations:** 1 Limb Function Reconstruction Center, Jing’an District Centre Hospital of Shanghai, Fudan University, Shanghai, China; 2 National Clinical Research Center for Aging and Medicine, Jing’an District Central Hospital, Fudan University, Shanghai, 200040 China; 3 Department of Cardiology, Tongji Hospital, School of Medicine, Tongji University, Shanghai, 200065 China

**Keywords:** Ferroptosis, Cardiovascular disease, Iron metabolism, Lipid peroxidation, System Xc-/GSH/GPX4, Therapeutic strategies

## Abstract

Ferroptosis, a regulated cell death modality driven by iron accumulation and lipid peroxidation, has emerged as a pivotal pathophysiological mechanism across a broad spectrum of cardiovascular diseases (CVDs), which remain the leading cause of global mortality. Although robust preclinical evidence indicates that modulation of ferroptosis attenuates myocardial and vascular injury, clinical translation is constrained by incomplete understanding of context-specific roles, the paucity of validated biomarkers, and the absence of targeted therapeutics with acceptable safety profiles. In this Review, we systematically characterizes the molecular architecture underlying ferroptosis, focusing on its core machinery governing iron homeostasis and lipid peroxidation, as well as the principal antioxidant defense systems that counteract this process. We subsequently survey the pathological contributions of ferroptosis across CVDs, detailing its involvement in atherosclerotic plaque instability, myocardial ischemia–reperfusion injury, heart failure progression, cardiomyopathies, and hypertensive cardiac remodeling. Furthermore, we evaluate emerging therapeutic strategies-ranging from iron chelation and radical-trapping antioxidants to GPX4-modulating agents and advanced nanomedicine-based delivery platforms-and critically appraise the landscape of candidate biomarkers indispensable for clinical translation, encompassing circulating lipid peroxidation products, iron metabolism indices, regulatory non-coding RNAs, and advanced imaging surrogates.By integrating mechanistic insights with translational perspectives, this Review positions ferroptosis as both a fundamental driver of cardiovascular pathology and a promising frontier for the development of precision diagnostics and targeted therapies aimed at mitigating the global burden of CVD.

## Introduction

Cardiovascular diseases (CVDs) remain the leading cause of global morbidity and mortality, encompassing a broad spectrum of disorders such as atherosclerosis, ischemic heart disease, heart failure, cardiomyopathies, hypertension, pulmonary arterial hypertension, stroke, and aortic dissection-that together account for nearly one-third of all deaths worldwide [1]. A fundamental driver of CVD progression is the irreversible loss of terminally differentiated cardiomyocytes and vascular cells [2, 3]. For decades, apoptosis and necrosis [4] were regarded as the dominant forms of regulated cell death in the cardiovascular system [5, 6]. However, the identification of ferroptosis in 2012 [7] marked a paradigm shift in our understanding of cardiovascular pathology. Ferroptosis is defined as an iron-dependent form of regulated cell death (RCD)driven by the accumulation of lipid peroxides and is mechanistically and morphologically distinct from other cell death modalities. Since its initial description, a rapidly growing body of evidence has implicated ferroptosis as a critical pathophysiological contributor across a wide spectrum of cardiovascular conditions [8], ranging from acute ischemic injury to chronic maladaptive remodeling.

Despite these advances, the role of ferroptosis in cardiovascular disease remains less well characterized than in fields such as oncology and neurodegeneration, where it has been more extensively investigated [9–11]. Current CVD research reveals a critical knowledge gap: although preclinical studies robustly demonstrate that ferroptosis inhibition attenuates tissue injury, the molecular pathways governing its activation are highly context dependent, and its definitive contribution to human disease progression remains incompletely defined. Moreover, ferroptosis is increasingly recognized as a double-edged sword—while its excessive activation promotes pathology, its precise modulation may offer novel therapeutic opportunities [12]. The accelarating pace of discovery, including the identification of new regulatory networks and the complex interplay with redox imbalance, metabolic reprogramming, and inflammation, underscores the need for a timely and comprhensive synthesis. Such a review is essential to consolidate current knowledge, resolve outstanding controversies, and define priorities for translating ferroptosis biology into cardiovascular medicine.

This review bridges the gap between the fundamental molecular machinery of ferroptosis and its burgeoning relevance to clinical cardiology. We provide an integrated synthesis of current knowledge, with a particular focus on linking mechanistic insights to translational potential. Key highlights include a systematic delineation of both core and emerging ferroptosis-regulatory networks, a detailed analysis of the pathological contributions of ferroptosis across distinct CVDs, and a critical evaluation of evolving therapeutic strategies ranging from small-molecule inhibitors to advanced nanomedicine-based approaches. Importantly, we also evaluate the landscape of candidate ferroptosis-related biomarkers, which are indispensable for clinical translation, and discuss the major challenges that must be addressed to advance ferroptosis-targeted interventions from preclinical models to patient care.

To achieve these aims, the review is structured to guide the reader logically from molecular mechanisms to clinical application. We first outline the core molecular machinery of ferroptosis, focusing on the interplay between iron homeostasis, lipid peroxidation, the GPX4–glutathione axis, and recently identified defense pathways. We then examine the growing body of evidence implicating ferroptosis in the pathogenesis of major cardiovascular disorders, including atherosclerosis, myocardial ischemia–reperfusion injury, cardiomyopathies, heart failure, and hypertension. Next, we assess emerging therapeutic strategies targeting ferroptosis, highlighting both their promise and the translational hurdles that remain. We subsequently address the urgent need for diagnostic and prognostic tools by summarizing current ferroptosis-related biomarkers, encompassing circulating indicators and advanced imaging surrogates. Finally, we conclude by outlining key unresolved questions and future directions, emphasizing the need to define context-specific mechanisms and develop precise, clinically viable anti-ferroptosis strategies for cardiovascular disease.

## Core molecular machinery governing ferroptosis

### The pro-ferroptotic axis: iron, lipids, and oxidative damage

#### Iron homeostasis in ferroptosis

Iron is one of the most important metallic elements in living organisms. Maintaining iron homeostasis is crucial for regulating iron uptake, sustaining normal physiological activities, and ensuring the proper functioning of internal organs [13]. Iron enters the body primarily through dietary iron consumption in the duodenum and upper jejunum. Heme iron, which is found primarily in meat and animal offal, has a higher absorption rate; non-heme iron, which is mainly present in plant-based foods, has a relatively lower absorption rate. Dietary non-heme iron is predominantly absorbed in its reduced Fe^2^⁺ form, following luminal reduction of Fe^3^⁺. Fe^3+^ can be combined with transferrin (TF) to form TF-Fe^3+^ complex circulating in the blood for various tissues to take up on demand [14]. The TF-Fe^3+^ complex circulating in the blood can bind to the transferrin receptor 1 (TfR1) on the cell membrane and then transfer to the endosome [15]. Within endosomes, Fe^3^⁺ is reduced to Fe^2^⁺ via the six transmembrane epithelial antigen protein 3 (STEAP3). Fe^2^⁺ is primarily transported into the cytoplasm via divalent metal transporter-1 (DMT1)DMT1, while certain ZIP family members (e.g. ZIP8/ZIP14) may contribute under specific pathological conditions. Subsequently, a portion of Fe^2^⁺ binds to ferritin heavy chain 1 (FTH1) in the cytoplasm and is oxidized to Fe^3^⁺, then combines with ferritin light chain (FTL) to form ferritin complexes stored within the cell; Concurrently, the remaining Fe^2^⁺ forms the labile iron pool(LIP) in the cytoplasm [16]. On one hand, Poly(rC)-binding proteins (PCBPs) act as cytosolic iron chaperones, facilitating the delivery of Fe^2^⁺ to ferritin and other non-heme iron–dependent proteins [17]. On the other hand, Fe^2^⁺ in the labile iron pool is redox-active and can act as a catalyst in the Fenton reaction [18, 19], reacting with hydrogen peroxide to produce highly reactive hydroxyl radicals, which can inflict oxidative damage on cellular components, including DNA, proteins, and cell membranes lipids, lead to the accumulation of lipid reactive oxygen species (ROS). Excess Fe^2^⁺ can be transported out of the cell via the transmembrane protein ferrotransporter 1 (FPN), which is the only known mammalian iron export protein. Mouse cardiomyocytes lacking FPN exhibit iron accumulation and demonstrate cardiac dysfunction [20]. In a lipopolysaccharide (LPS)-induced endotoxemia rat model, FPN gene knockdown promoted iron accumulation and oxidative reactions, and was associated with ferroptosis and de novo atrial fibrillation [21].

Under conditions of limited iron availability, Ferritin can be targeted by specific nuclear receptor coactivator 4(NCOA4) [22], which facilitate its degradation by lysosomal enzymes and the stored iron is released. Intracellularly, iron storage proteins, transporters, and other factors are regulated to ensure adequate but nontoxic iron levels within cells [23]. The body lacks active iron clearance mechanisms, so iron homeostasis is maintained through regulation of iron absorption. Excess iron (including heme and non-heme iron) is primarily stored as ferritin and hemosiderin within hepatic parenchyma and the reticuloendothelial system, particularly in reticuloendothelial cells of the bone marrow, spleen, and liver [24].

The regulation of iron homeostasis in the body is primarily mediated by hepcidin-produced by the liver [25]. When iron load increases (e.g., elevated iron stores, high serum iron levels) [26], elevated hepcidin directly binds to membrane-bound FPN, promoting its internalization and degradation within intestinal epithelial cells, thereby inhibiting iron absorption into the bloodstream [27]. Under hypoxic conditions, hepcidin expression decreases under the influence of hypoxia-inducible factor (HIF), thereby promoting iron release [28].

Iron overload, first hypothesized to contribute to cardiovascular risk by Sullivan in the late twentieth century [29], is characterized by elevated transferrin saturation and the appearance of non-transferrin-bound iron (NTBI). At the cellular level, NTBI is considered part of the LIPs. Cellular iron homeostasis is regulated at the post-transcriptional level through iron-responsive element (IRE)–iron regulatory protein (IRP) interactions, which control the translation of mRNAs encoding ferritin, FPN, TfR1, and DMT1.Comprising ferrous iron bound by low-affinity iron chelators, NTBI is a direct consequence of oxidative stress in the circulation and tissue iron loading [30]. It represents the kinetic equilibrium between iron excretion into serum, TF binding, clearance from the circulation, and utilization within the circulation [31]. NTBI exhibits high redox reactivity, leading to oxidative damage in various cells and organs [32]. The accumulation of NTBI causes cellular damage in organs, including the heart. A study [33] has found that elevated ferritin levels increase the risk of new-onset heart failure, particularly in women.

**Fig. 1:**
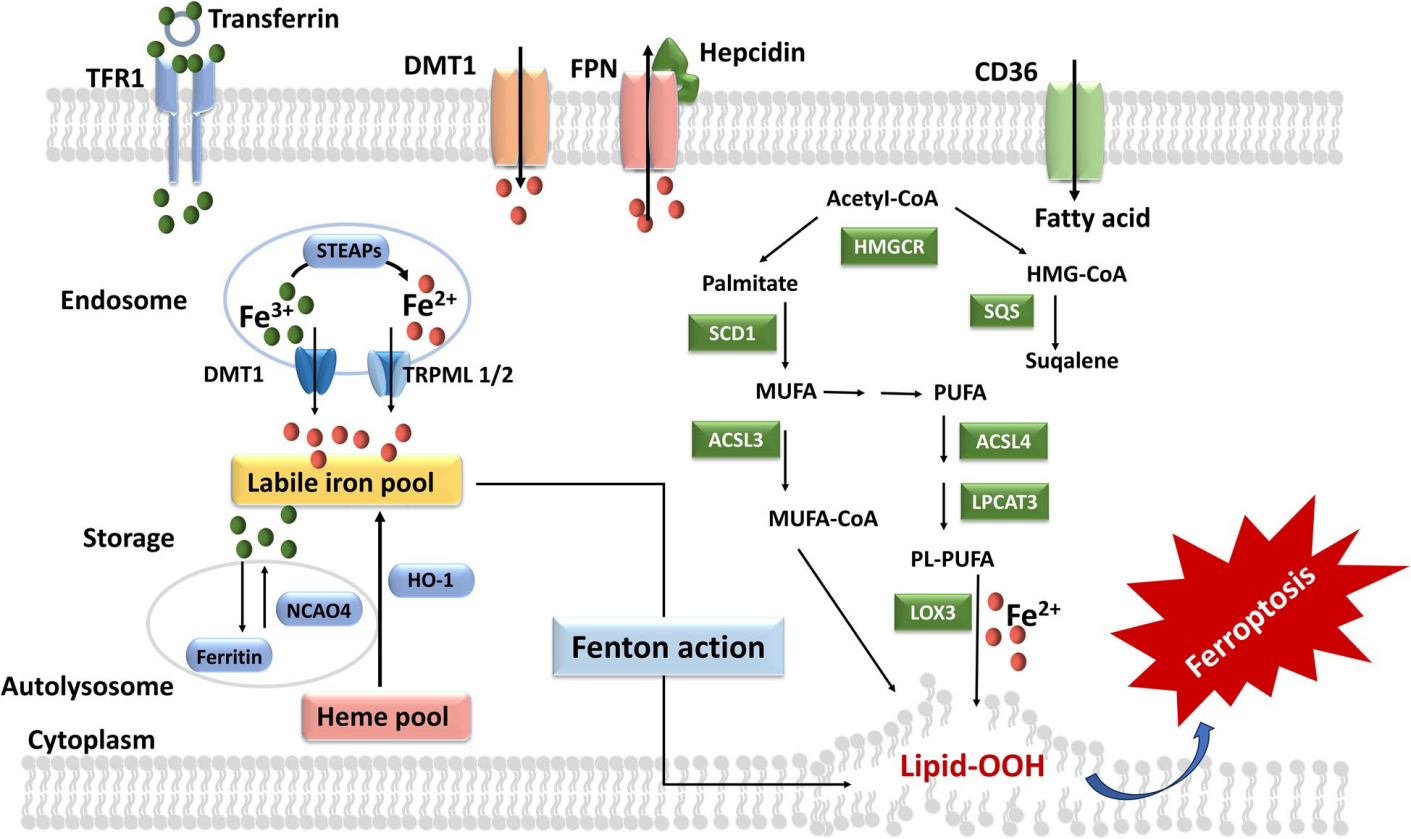
Dysregulation of iron metabolism and peroxidation of PUFAs can induce ferroptosis [36]. Transferrin (TF) delivers Fe^3^⁺ via TFR1, while DMT1 mediate Fe^2^⁺ uptake. Inside endosomes, STEAP metalloreductases convert Fe^3^⁺ to Fe^2^⁺, which is released to the cytosol by DMT1 and TRPML1/2 (lysosomal cation channel mucolipin). Cytosolic Fe^2^⁺ forms the labile iron pool, balanced by ferritin storage through NCOA4-dependent ferritinophagy and export by ferroportin (FPN) regulated by hepcidin. Excess iron drives hydroxyl radical generation through the Fenton reaction, promoting lipid oxidation. In parallel, acetyl-CoA, generated through lipid and glucose metabolism, fuels the mevalonate pathway via HMG-CoA reductase (HMGCR) and squalene synthase (SQS) to produce squalene and CoQ_10_, both contributing to antioxidant defense. Palmitate, converted by stearoyl-CoA desaturase 1 (SCD1) to monounsaturated fatty acids (MUFAs), protects against ferroptosis. Fatty acid uptake (CD36) and metabolism through ACSL3/4 and LPCAT3 promote PUFA incorporation into phospholipids (PL-PUFAs) and formation of lipid hydroperoxides (Lipid-OOH). The imbalance between these iron- and lipid-metabolic pathways disrupts redox homeostasis and culminates in ferroptosis

At the cellular level, iron overload promotes ferroptosis primarily through reactive oxygen species generation. Redox-active Fe^2+^ catalyzes lipid peroxidation via the Fenton reaction [34], initiating self-propagating peroxidative chain reactions that culminate in ferroptotic cell death [35] (Fig. 1).

#### Lipid peroxidation in ferroptosis

Lipid peroxidation plays a crucial role in ferroptosis. Polyunsaturated fatty acids (PUFAs) and their derivatives serve as primary substrates for lipid peroxidation, and the cellular abundance of PUFA-containing phospholipids largely determines susceptibility to ferroptotic stress. In particular, arachidonic acid (AA) and adrenic acid (AdA) are highly peroxidizable fatty acids that can be incorporated into membrane phospholipids and subsequently drive lipid peroxide propagation [35]. The biosynthetic incorporation of these PUFAs into phospholipids requires two key enzymes: acyl-CoA synthetase long-chain family member 4 (ACSL4) and lysophosphatidylcholine acyltransferase 3 (LPCAT3). ACSL4 catalyzes the ligation of free long-chain PUFAs to coenzyme A (CoA), generating PUFA–CoA esters [37]. These activated intermediates, including AA–CoA and AdA–CoA, are subsequently esterified into phospholipids by LPCAT3 [38],producing PUFA-enriched phosphatidylethanolamine species such as PE-AA and PE-AdA, which represent key substrates for lipid peroxidation during ferroptosis. Consistently, knockout of LPCAT3 in lung epithelial cells conferred resistance to ferroptosis induced by the Ras-selective lethal compound 3 (RSL3), and reduced expression of ACSL4 or LPCAT3 diminished the availability of peroxidizable phospholipid substrates [39], thereby inhibiting ferroptosis and further emphasizing the critical role of PUFA metabolism in ferroptosis.

Reactive oxygen species react with PUFAs in phospholipid membranes,initiating lipid peroxidation chain reactions that ultimately compromise membrane integrity and trigger ferroptotic cell death. Erastin-induced ferroptosis has been closely associated with pathological ROS accumulation [13], and suppression of ROS generation can inhibit ferroptosis [40]. Multiple cellular sources contribute to ROS production; among them, iron-driven radical formation through Fenton chemistry is a major initiator and amplifier of lipid peroxidation [41]. Specifically, ferrous iron (Fe^2^⁺) reacts with hydrogen peroxide (H₂O₂) to generate highly reactive hydroxyl radicals [34], which initiate non-enzymatic lipid autoxidation and accelerate the propagation of lipid peroxides. Hydroxyl radicals can serve as targets for regulating ferroptosis (Fig. 1) [36].

In addition to non-enzymatic oxidation, enzymatic lipid peroxidation mediated by lipoxygenases (LOXs) also contributes to ferroptosis. LOXs are non-heme iron-dependent dioxygenases capable of oxidizing PUFA-containing phospholipids into phospholipid hydroperoxides (PLOOHs). Pharmacological inhibition of LOX12/15 with PD146176 reduces ferroptosis in GPX4-deficient cells [42], and LOX inhibitors have been reported to suppress erastin-induced ferroptosis in certain experimental models [43].

In cardiomyocytes, excessive ROS and lipid peroxidation can further disrupt calcium homeostasis and excitation–contraction coupling, thereby exacerbating contractile dysfunction. Oxidative modifications may impair the expression or activity of sarcoplasmic reticulum Ca^2^⁺-ATPase (SERCA2a), leading to defective Ca^2^⁺ reuptake during diastole, sustained cytosolic Ca^2^⁺ elevation further causes diastolic dysfunction and delayed relaxation [44].

Notably, antioxidant treatment with N-acetylcysteine (NAC) reversed these abnormalities in metabolic syndrome rat hearts [44]. Moreover, ROS also leads to cardiac ryanodine receptor (RyR2) Ca^2+^ channel hyperphosphorylation or oxidation, resulting in aberrant Ca^2^⁺ leak from the sarcoplasmic reticulum, which contributes to contractile dysfunction and arrhythmogenesis [45].

### The cellular defense network against ferroptosis

#### The canonical system Xc-/GSH/GPX4 pathway

**Fig. 2:**
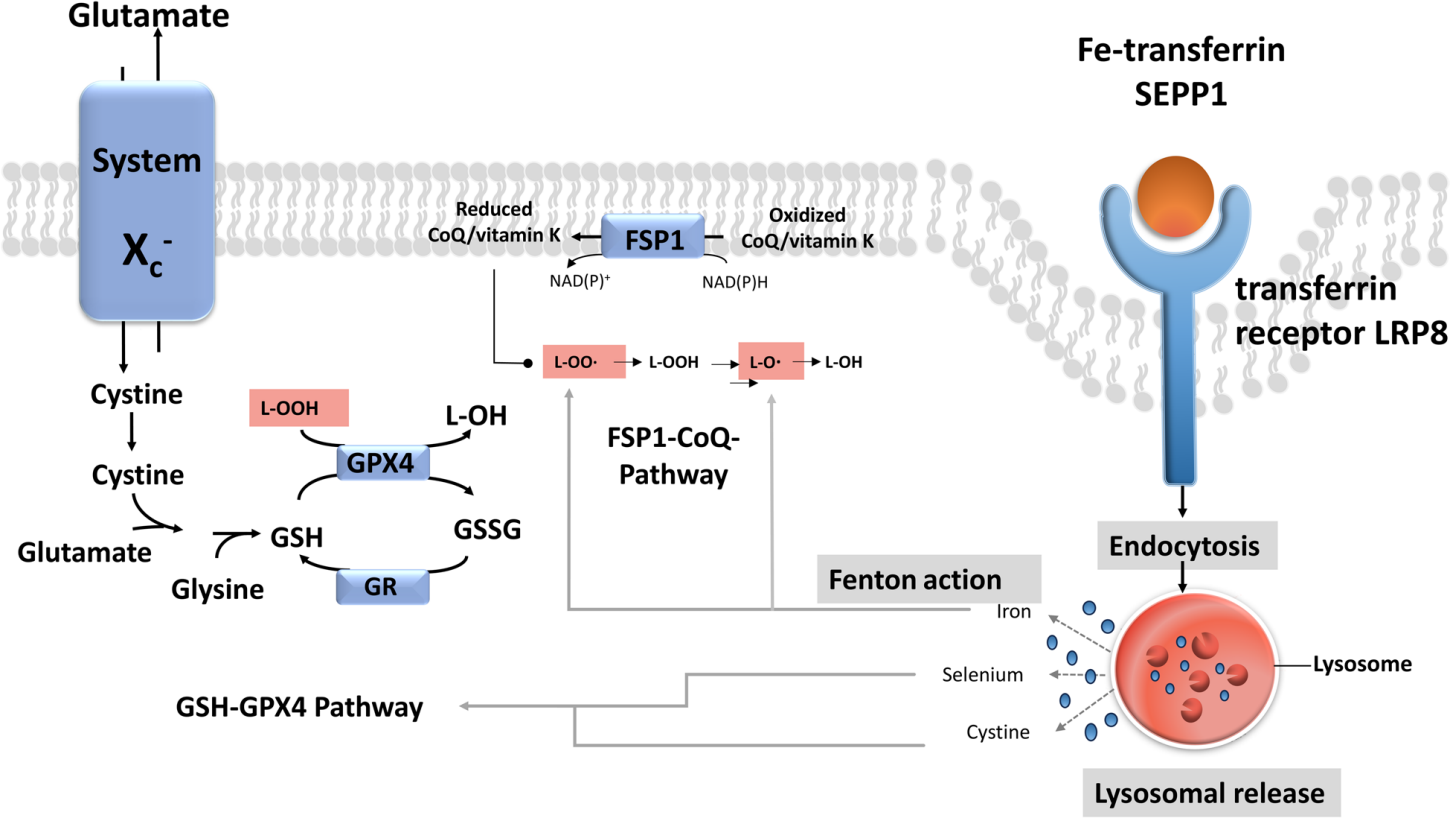
GPX4 inhibition and GSH consumption in ferroptosis [59]. The system Xc⁻ antiporter exchanges extracellular cystine for intracellular glutamate. Imported cystine is reduced to cysteine, enabling glutathione (GSH) synthesis. GSH is oxidized to glutathione disulfide (GSSG) and subsequently reduced back to GSH by glutathione reductase (GR). GSH serves as a cofactor for GPX4, which converts lipid hydroperoxides (L-OOH) into non-toxic lipid alcohols (L-OH), preventing membrane damage. In parallel, FSP1 uses nicotinamide adenine dinucleotide phosphate (NAD(P)H) to regenerate coenzyme Q_10_ (CoQ) and vitamin K, which neutralize lipid peroxyl radicals (LOO•) and terminate lipid peroxidation. Receptor-mediated endocytosis also affects ferroptosis sensitivity: transferrin receptor–mediated iron uptake promotes ferroptosis, whereas SEPP1–LRP8 signaling provides protection. Within lysosomes, released iron and selenium drive opposing outcomes—iron catalyzes radical formation that enhances lipid peroxidation, while selenium and cysteine support GSH and GPX4 synthesis, reinforcing antioxidant defense

The dynamic equilibrium between glutathione (GSH) biosynthesis and consumption is crucial for protecting cells from oxidative damage. GSH can be produced enzymatically from intracellular L-cysteine (L-Cys), glutamate (Glu), and glycine (Gly) [46]. Among these, L-Cys is the most important precursor, primarily acquired through the glutamate/cysteine antiporter system Xc^−^ which plays a key role (Fig. 2) in maintaining intracellular glutathione (GSH) levels by mediating the uptake of cystine, which is subsequently reduced to cysteine for GSH synthesis [13]. This system comprises the 12-transmembrane domain solute carrier family 3 member 2 (SLC3A2) and the single-transmembrane domain solute carrier family 7 member 11 (SLC7A11) [47]. SLC7A11 as a functional subunit of System Xc-, has a high specificity for cystine, and its role is to participate in extracellular uptake of cystine and release of Glu. Activating transcription factor 3 (ATF3) binds to the SLC7A11 promoter to inhibit System Xc- and promote Erastin-induced ferroptosis [48]. Erastin can inhibit the activity of System Xc- by combining the key amino acid residue Phe254 with the light chain xCT (encoded by the SLC7A11) [49] and changing the spatial conformation of the transmembrane (TM) domain TM6b of xCT [50]. In addition, Knockout of the SLC7A11 gene increases cellular susceptibility to ferroptosis by blocking L-cysteine uptake [51]. Conversely, SLC7A11 overexpression enhances cellular resistance to ferroptosis. Enhancing the activity of system Xc⁻ increases cysteine availability, promotes GSH biosynthesis, also influences the functional activity of glutathione-dependent enzymes. GSH functions as a reducing substrate for glutathione peroxidase 4 (GPX4), which reduces peroxides to inactivate ROS [52]. Glutathione (GSH) functions as an essential redox cofactor in cellular antioxidant defense. Rather than directly detoxifying lipid hydroperoxides (LOOHs), GSH serves as the reducing substrate for glutathione peroxidase 4 (GPX4), which catalyzes the conversion of LOOHs into non-toxic lipid alcohols. Through this GPX4-dependent reaction, GSH prevents the accumulation of lipid peroxides and thereby suppresses the initiation and propagation of ferroptosis. GPX4 utilizes GSH as an essential reducing substrate, accepting electrons from the thiol group of GSH to catalyze the reduction of phospholipid hydroperoxides. Depletion of intracellular GSH compromises GPX4 catalytic activity, leading to the accumulation of lipid peroxides and ultimately triggering ferroptosis [53]. Genetic ablation of GPX4 induces ferroptosis in multiple mouse cell types, underscoring its indispensable role in cellular redox homeostasis. Conversely, adequate selenium availability promotes GPX4 biosynthesis and activity, thereby enhancing cellular resistance to ferroptosis [54]. In recent years, an increasing number of small molecules have been identified that modulate ferroptosis sensitivity through the GPX4–GSH axis. For example, ML210 is a covalent GPX4 inhibitor that induces ferroptosis and has been widely used as a chemical probe to study ferroptotic mechanisms [55]. Similarly, pharmacological inhibition of GPX4 by Ras-selective lethal compound 3 (RSL3) blocks the enzymatic detoxification of lipid hydroperoxides, resulting in excessive lipid peroxidation and ferroptotic cell death [56–58] (Fig. 2) [59].

#### FSP1-CoQ10 pathway

Ferroptosis suppressor protein 1 (FSP-1, also known as flavoprotein apoptosis-inducing factor mitochondria-associated 2, AIFM2) has been identified as a glutathione-independent ferroptosis inhibitor [60]. It protects cells from ferroptosis regardless of cellular GSH levels, GPX4 activity, or p53 status. Its activity is mediated by extramitochondrial ubiquinone (also known as coenzyme Q_10_, CoQ_10_) [60]. The reduced form of coenzyme Q_10_, ubiquinol (CoQH_2_), prevents lipid peroxidation, and FSP1 maintains CoQ_10_ regeneration in an NADPH-dependent manner. FSP1 recruitment to the plasma membrane as a redox enzyme, requires canonical N terminal myristoylation motif, thereby conferring ferroptosis resistance [61].

Disruption of the FSP1-coenzyme Q_10_ pathway induces ferroptosis. Loss of FSP1 function, whether induced by the small-molecule inhibitor iFSP1 or by gene deletion, promotes ferroptosis. Another ferroptosis inducer, FIN56, promotes GPX4 protein degradation and also interferes with FSP1-CoQ_**10**_ pathway activation by inhibiting coenzyme Q_10_ activity through activation of squalene synthase in the mevalonate pathway [62]. Studies in mice indicate that doxorubicin can activate FSP1 translocation [63] through lipid peroxidation products in the heart, thereby inducing cardiac injury. However, further research is needed to elucidate the precise role of FSP1 in cardiovascular diseases.

#### GCH1-BH4-DHFR pathway

The guanosine 5'-triphosphate (GTP) cyclohydrolase-1 (GCH1) tetrahydrobiopterin (BH4) pathway is a key non-GPX4 ferroptosis regulatory system. GTP serves as the precursor for BH4 synthesis, a process requiring three enzymatic steps catalyzed by GCH1, 6-propylthio-tetrahydropterin synthase (PTS), and spirodactyl reductase (SPR), with GCH1 acting as the rate-limiting enzyme [64]. Genetic or pharmacological inhibition of GCH1 leads to BH4 deficiency, driving cells toward peroxisome accumulation and ferroptosis [65]. Conversely, GCH1 overexpression enhances BH4 biosynthesis and reduces ROS production [66, 67].

BH4 pairs with dihydrobiopterin (BH2) to form a redox cycle that scavenges endogenous oxidative radicals, protects lipid membranes, and inhibits ferroptosis [68]. BH2 enables BH4 regeneration, catalyzed by dihydrofolate reductase (DHFR) with NADP +/NADPH as cofactors. Studies reveal that DHFR inhibition induces ferroptosis in GCH1-knockout cells, while BH4 supplementation directly reverses this process [65, 68]. Furthermore, BH₄ may promote coenzyme Q₁₀ production by influencing the synthesis of its precursor, 4-OH-benzoate. These mechanisms link the GCH1-BH₄-DHFR axis to the FSP1-coenzyme Q₁₀ axis, enabling coordinated and precise regulation of ferroptosis.

#### Mitochondrial DHODH-CoQH₂ system

Dihydroorotate dehydrogenase (DHODH) is an enzyme located in the inner mitochondrial membrane (IMM) that reduces CoQ to CoQH₂, thereby neutralizing peroxy radicals in mitochondrial lipids and preventing ferroptosis [69, 70]. The presence of DHODH in mitochondria and its capacity to neutralize lipid peroxides support its protective role against ferroptosis, while DHODH deficiency has been demonstrated to induce ferroptosis [71]. DHODH-CoQH₂ and GPX₄ function independently but both reduce CoQ to CoQH₂ [72]. Supplementation with DHO attenuates the GPX4 inhibition-induced increase in the anti-ferroptosis defense network in mitochondria [73]. Consequently, interfering with DHODH in ferroptosis has emerged as a promising therapeutic target for various diseases [71, 74, 75].

### The central role of mitochondria

#### Mitochondria as a hub for iron and ROS metabolism

Mitochondria are semi-autonomous organelles enclosed by a double-membrane system, consisting of the outer mitochondrial membrane (OMM) and the highly folded inner mitochondrial membrane (IMM) that forms cristae [76, 77]. Notably, mitochondria harbour a labile iron pool with strong redox activity [41], positioning these organelles as a critical hub for iron-dependent oxidative metabolism. Iron is the most abundant transition metal within mitochondria and is indispensable for multiple physiological processes, including the biosynthesis of iron–sulfur clusters (Fe–S), heme, and other essential cofactors that support electron transport and diverse enzymatic reactions [17, 78, 79]. Mitochondrial ferritin (FtMt), a mitochondria-localized iron storage protein with iron-binding affinity comparable to cytosolic ferritin [80], contributes to the maintenance of mitochondrial iron homeostasis [81]. Through its ferroxidase activity, FtMt prevents the accumulation of redox-active ferrous iron and restrains mitochondrial reactive oxygen species (mtROS) generation [82]. Accordingly, FtMt dysfunction promotes mitochondrial iron overload and excessive mtROS accumulation, thereby increasing cellular susceptibility to ferroptosis [83]. Iron overload can induce profound mitochondrial dysfunction, characterized by impaired mitochondrial respiration, elevated mtROS production, mitochondrial membrane depolarization, and mitochondrial swelling [84, 85]. Thus, disruption of mitochondrial iron homeostasis may amplify oxidative injury to mitochondrial proteins, lipids, and DNA, compromise ATP generation, and ultimately trigger energy stress that converges on ferroptotic cell death.

Voltage-dependent anion channels (VDACs) are highly abundant transport proteins located in the OMM and mediate the exchange of metabolites, including adenosine diphosphate (ADP), adenosine triphosphate (ATP), and respiratory substrates [86, 87]. Elevated VDAC expression has been associated with enhanced vulnerability to oxidative stress. In a mouse model of myocardial ischemia, the ferroptosis inhibitor liproxstatin-1 (Lip-1) attenuated ischemia/reperfusion (I/R)-induced GPX4 depletion and reduced mitochondrial ROS production, accompanied by decreased VDAC1 expression and preservation of mitochondrial ultrastructural integrity. These effects collectively conferred cardioprotection and reduced infarct size [88, 89].

Mitochondrial metabolism also contributes to ferroptotic sensitivity through nutrient utilization and bioenergetic rewiring.Glutamine is essential for ferroptosis, and glutamine synthase 2 (GLS2), a key regulator of glutaminolysis, has been demonstrated to promote ferroptosis [90].

**Fig. 3:**
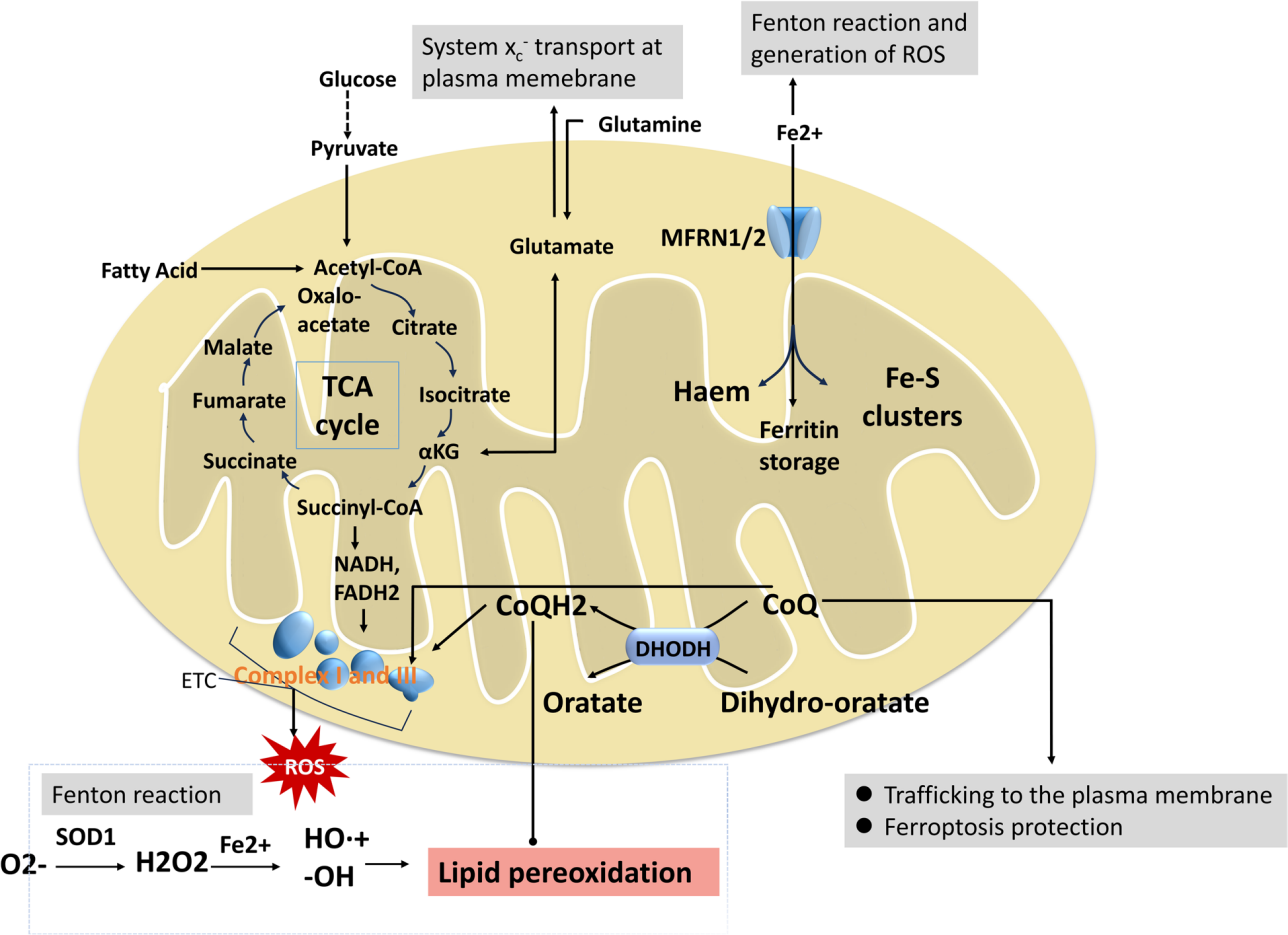
Mitochondrial metabolism and iron handling in ferroptosis [59]. Glucose and amino acid metabolism via glycolysis and the TCA cycle generate NADH and FADH₂, fueling the mitochondrial electron transport chain (ETC). Electron leakage at Complexes I and III produces superoxide (O₂•⁻), which is converted to H₂O₂ and, through the Fenton reaction (Fe^2^⁺ → HO•), yields highly reactive radicals. Glutamine catabolism supplies glutamate for the system Xc⁻ antiporter and forms α-ketoglutarate (αKG), enhancing mitochondrial ROS and ferroptosis sensitivity. Mitoferrin-1/2 (MFRN1/2) import iron across the inner mitochondrial membrane, supporting Fe–S cluster and hame synthesis. Within mitochondria, dihydroorotate dehydrogenase (DHODH) reduces coenzyme Q (CoQ) to CoQH₂, functioning independently of GPX₄ to detoxify lipid peroxides. DHODH deficiency induces ferroptosis, whereas CoQH₂ generation provides antioxidant defense

The mitochondrial tricarboxylic acid (TCA) cycle operates within the matrix and generates intermediates that can sustain ferroptosis-promoting metabolic programs. Notably, α-ketoglutarate (α-KG)- mimic the role of glutamine in ferroptosis, and its downstream metabolites, including succinate and fumarate, potentiate cysteine depletion-induced ferroptosis [41]. In parallel, citrate synthase supports fatty acid biosynthesis, whereas acyl-CoA synthetase family member 2 (ACSF2) contributes to fatty acid activation, providing lipid substrates that can fuel peroxidation reactions [41]. Moreover, pyruvate dehydrogenase kinase 4 (PDK4), localized to the IMM, has been implicated in metabolic regulation of ferroptosis by inhibiting pyruvate oxidation and altering lipid metabolic pathways, thereby modulating lipid peroxidation-dependent ferroptotic vulnerability [91] (Fig. 3) [59].

#### Mitochondrial contribution to lipid metabolism

Mitochondria are key organelles in lipid catabolism and redox metabolism and interact extensively with the ER to coordinate lipid homeostasis. In the context of ferroptosis, lipid remodelling enzymes that enrich PUFA-containing phospholipids—the primary substrates for lipid peroxidation—are particularly important. Among these, ACSL4 catalyses the activation of PUFAs, such as arachidonic acid, into their corresponding acyl-CoA derivatives, whereas LPCAT3 incorporates these activated PUFAs into membrane phospholipids through phospholipid remodelling. This metabolic wiring generates oxidizable PUFA-phospholipid species that can subsequently undergo enzymatic peroxidation (for example, via lipoxygenases) or non-enzymatic oxidation driven by iron-dependent reactive oxygen species, thereby establishing a lipid network that amplifies ferroptotic vulnerability.

ACSL4 is widely recognized as a key pro-ferroptotic determinant and has been proposed as a molecular indicator of ferroptosis susceptibility [92]. Consistently, upregulation of ACSL4 sensitizes cells to ferroptosis, whereas genetic silencing or pharmacological inhibition of ACSL4 suppresses ferroptotic cell death. Notably, thiazolidinediones (TZDs) have been reported to inhibit ACSL4 activity, suggesting a potential strategy for therapeutic modulation of ferroptosis [37]. Beyond its metabolic role, ACSL4 expression is also regulated by upstream signaling pathways. The E-cadherin–Merlin–Hippo–YAP axis has been shown to control ACSL4 transcription, thereby linking cell–cell contact signaling to ferroptosis susceptibility [93], and further supporting ACSL4 as a pivotal mediator of ferroptotic regulation [94]. Moreover, the PRDX2–MFN2–ACSL4 pathway has been implicated in mitochondria-associated ferroptosis and has been suggested to exert protective effects in the diabetic cardiac microvascular system [95].Collectively, these findings highlight that mitochondrial–lipid crosstalk, coordinated through lipid remodeling programs, represents a key determinant of ferroptotic sensitivity in cardiovascular pathology.

#### Mitochondrial dysfunction and structural changes in ferroptosis

Ferroptosis is distinguished from other forms of regulated cell death by its characteristic mitochondrial morphology. Hallmark ultrastructural features include mitochondrial shrinkage, increased membrane density, loss or collapse of cristae, condensation of the matrix, and, in some contexts, rupture of the outer mitochondrial membrane [96]. These morphological signatures are widely considered to reflect overwhelming oxidative stress and lipid peroxidation, linking ferroptotic execution to profound mitochondrial injury. Given that mitochondrial architecture is tightly coupled to bioenergetic capacity, disruption of mitochondrial structural integrity has important consequences for ATP generation and mitochondrial–cytoplasmic metabolite exchange [97, 98].

At the ultrastructural level, ferroptotic mitochondria typically appear condensed and fragmented, with markedly reduced size and electron-dense membranes. Cristae are frequently diminished or absent, and discontinuities of the outer mitochondrial membrane are commonly observed [99]. Under conditions of GPX4 inhibition or severe oxidative stress, mitochondria may also exhibit swelling or lamellar remodeling, often accompanied by near-complete cristae depletion [100]. These structural derangements coincide with robust mitochondrial lipid peroxidation. For instance, genetic ablation of GPX4 promotes the accumulation of oxidized cardiolipin within mitochondriaa [54], and lipid peroxidation has been reported to emerge initially in mitochondrial membranes before propagating to other cellular compartments.

Functionally, ferroptotic stimuli precipitate collapse of mitochondrial proton gradient. Exposure to canonical ferroptosis inducers, including erastin, RSL3, and glutamate, triggers a rapid loss of mitochondrial membrane potential (Δψm), thereby impairing oxidative phosphorylation and compromising respiratory ATP production [101, 102]. Notably, perturbation of mitochondrial dynamics modulates this vulnerability: deletion of the fission regulator dynamin-related protein 1 (Drp1) prolongs Δψm maintenance and confers resistance to ferroptotic death [103], whereas ferroptotic signaling is associated with Drp1 activation and mitochondrial fragmentation. In parallel, VDAC1 oligomerization has been implicated in ferroptosis-associated mitochondrial dysfunction, and pharmacological blockade of VDAC1 oligomerization suppresses mitochondrial ROS accumulation and lipid peroxidation during ferroptosis [99]. Together, these mitochondrial events — encompassing cristae loss, membrane destabilization, Δψm collapse, and aberrant fission — converge to disrupt oxidative metabolism and promote the energetic failure that accompanies ferroptotic cell death.

### Upstream regulatory pathways and transcription factors

#### p53 pathway

p53 is a master stress-responsive transcription factor that governs cell-cycle arrest, senescence and apoptosis, and its functional inactivation represents a central event in tumorigenesis; accordingly, TP53 is widely regarded as a canonical tumour suppressor gene [104]. Beyond its established roles in apoptosis, p53 can also modulate ferroptosis through metabolic and redox regulatory programs. Mechanistically, p53 represses the expression of SLC7A11, thereby limiting cellular cystine uptake, reducing GSH biosynthesis and compromising GPX4-dependent detoxification of lipid hydroperoxides. This redox imbalance promotes the accumulation of lipid ROS and sensitizes cells to ferroptotic death [104, 105]. Consistently, knockdown of p53 alleviates the repression of SLC7A11 and partially restores antioxidant capacity, thereby reducing ferroptosis susceptibility [106]. In addition to suppressing SLC7A11, p53 has also been reported to activate ALOX12, further enhancing lipid peroxidation and ferroptotic signalling [107].

Importantly, p53-mediated ferroptosis can also occur independently of the canonical SLC7A11–GSH–GPX4 axis through a distinct p53–SAT1–ALOX15 pathway. p53 induces the expression of spermidine/spermine N1-acetyltransferase 1 (SAT1), and its inhibition partially abrogates p53-mediated ferroptosis. This pathway depends on ALOX15 rather than ALOX12, which belongs to the same eukaryotic lipoxygenase family [108].

#### AMPK signaling pathway

Adenosine monophosphate -activated protein kinase (AMPK) is a key regulator of ATP homeostasis and exerts dual pro- and anti-ferroptotic effects depending on its substrates. AMPK-mediated phosphorylation of BECN1(beclin 1) promotes ferroptosis by inhibiting SLC7A11 activity or inducing autophagy [109, 110] whereas AMPK-mediated phosphorylation of ACACA suppresses ferroptosis by inhibiting fatty acid biosynthesis [111].

#### NRF2 -HO-1 pathway

Nuclear factor erythroid 2–related factor 2 (NRF2) is a master regulator of endogenous antioxidant defenses and plays a pivotal role in maintaining iron homeostasis [112]. NRF2 transcriptionally regulates key components of iron metabolism, including ferritin heavy and light chains (FTH1/FTL), which mediate iron sequestration, and ferroportin (SLC40A1), the primary cellular iron exporter [113]. Beyond iron handling, NRF2 modulates the GSH antioxidant system by inducing the expression of enzymes required for GSH biosynthesis, such as glutamate–cysteine ligase catalytic and modifier subunits (GCLC/GCLM), glutathione synthase (GSS), and the cystine/glutamate antiporter subunit SLC7A11 [114]. NRF2/HO-1 constitutes a primary signaling pathway regulating ferroptosis. As a transcription factor, NRF2 translocates to the nucleus and activates genes containing antioxidant response elements (AREs), including heme oxygenase-1 (HO-1)and NRF2 itself [115]. HO-1 is an enzyme with anti-inflammatory and antioxidant stress effects [116]. NRF2 is a key regulator of HO-1 expression. HO-1 catalyzes heme degradation into carbon monoxide, ferrous iron, and biliverdin [117], which is subsequently reduced to bilirubin—both of which possess potent reactive oxygen species (ROS)-scavenging properties. Activation of the NRF2–HO-1 axis represents a critical cellular defense mechanism against oxidative stress. However, because HO-1 activity also liberates redox-active iron, its impact on ferroptosis is highly context dependent and relies on the coordinated upregulation of iron sequestration and export pathways. Under conditions where antioxidant and iron-buffering systems are adequately engaged, NRF2 activation enhances GPX4, superoxide dismutase (SOD), and GSH levels, reduces lipid hydroperoxides, malondialdehyde (MDA), and ROS accumulation [118], and thereby suppresses ferroptotic cell death.

#### NF-κB

NF-κB is a classical transcription factor, and studies indicate that ferroptosis involves the NF-κB signaling pathway. Dimethyl fumarate (DMF), as an activator of NF-E2-related factor 2 (NRF2), leads to the upregulation of IκBα and suppression of NF-κB signaling pathway activation, thereby promoting the expression of key ferroptosis factors heme oxygenase 1 (HMOX1), NADPH quinone oxidoreductase 1 (NQO1), and GPX4. In a rat model of cognitive impairment induced by bilateral cerebral artery occlusion, it alleviated neuroinflammation, improved chronic cerebral hypoperfusion, and ultimately protected cells from oxidative stress and ferroptosis [119].

#### MAPK pathway

Activation of the MAPK pathway–dependent inflammatory cascade has been implicated in the regulation of ferroptosis. In a neonatal rat model of hypoxia–ischemia, stimulation of the TLR4–p38 MAPK signaling pathway induced the production of proinflammatory cytokines, including IL-1β, IL-6, and IL-18, while simultaneously downregulating the ferroptosis-related proteins SLC7A11 and GPX4, thereby promoting neuroinflammation and ferroptotic cell death [120]. Consistently, oxygen–glucose deprivation (OGD) was found to activate the same TLR4–p38 MAPK pathway, resulting in elevated malondialdehyde (MDA) accumulation and neuronal ferroptosis. Notably, pharmacologic inhibition of p38 with SB203580 reversed these effects by restoring SLC7A11 and GPX4 expression, thus alleviating OGD-induced ferroptosis in neuronal cells [120].

### Regulation by non-coding RNAs

Non-coding RNAs (ncRNAs) are functional RNA molecules classified into distinct types based on length and structure, including microRNAs (miRNAs) [121], long non-coding RNAs (lncRNAs) [122], circular RNAs (circRNAs) [123], transfer RNAs (tRNAs) [124], and ribosomal RNAs (rRNAs) [125]. ncRNAs play crucial roles in various biological processes, including chromatin modification, alternative splicing, and DNA replication, by regulating the expression of target genes [126]. Growing evidence indicates that ncRNAs, particularly miRNAs, lncRNAs, and circRNAs, serve as key regulators at multiple junctures of ferroptosis [127–129]. They participate in ferroptosis by modulating iron, ROS, and ferroptosis-associated amino acid metabolism (Table 1).

**Table 1:** Noncoding RNAs regulate ferroptosis

**ncRNA**	**Target**	**Mechanism**	**Effects**	**Refs**
**miR-23a-3p**	DMT1	ACSL4	Inhibits ferroptosis	[130]
**miRNA-17–92**	A20	Inhibits A20-ACSL4 axis	protects endothelial cells from ferroptosis	[131]
**miR-135b-3p **	GPX4	Inhibits GPX4 pathway	promotes ferroptosis	[132]
**miR-15a-5p **	GPX4	Inhibits GPX4 pathway	Promotes ferroptosis	[133]
**miR-29b-3p**	PTX3	Inhibits PTX3 pathway	Promotes ferroptosis	[134]
**miR-30d**	ATG5	Inhibits ATG5 pathway	Inhibits ferroptosis	[135]
**miR-190a-5p**	GLS2	Activates GLS2 pathway	Inhibits ferroptosis	[136]
**miR-199a-5p**	GSH/GSSG, GPX4	Akt/eNOS pathway	Promotes ferroptosis	[137]
**circRNA1615**	miRNA152-3p	Inhibits miRNA152-3p/LRP6	Inhibits ferroptosis	[138]
**circSnx12**	miR-224-5p	Activates FTH1	Promotes ferroptosis	[139]
**lncRNA- KCNQ1OT1 **	miR-7-5p	Inhibits KCNQ1OT1/miR-7-5p	Promotes ferroptosis	[140]
**lncRNA- XXYLT1-AS2 **	AKT ↑ and NF-κB	Activates AKT-NF-κB pathway	Inhibits ferroptosis	[141]
**lncRNA-UCA1**	miR-873-5p	Inhibits miR-873-5p/XIAP axis	Inhibits ferroptosis	[142]
**lncRNA-p21**	MDM2	Inhibits the formation of the p300-p53 complex	Promotes ferroptosis	[143]

MicroRNAs (miRNAs) are small ncRNAs consisting of 18–25 nucleotides. They suppress specific gene expression by directly binding to the 3'-untranslated region (3'UTR) of target mRNA transcripts, participating in nearly all physiological and pathological processes [121, 144, 145]. Overexpression of miR-15a-3p increases ROS, intracellular Fe^2^⁺, and malondialdehyde accumulation in CRC cells by binding to the 3′UTR of GPX4 to suppress its expression [146]. Nrf2, which activates downstream antioxidant factors as a transcription factor, is also a key miRNA target in ferroptosis [147]. Zhang et al. demonstrated that miR-27a significantly exacerbates ferroptosis by directly targeting Nrf2, with its antagonists reversing miR-27a-mediated effects [148]. miR-199a-5p was reported to stimulate ferroptosis-induced organ cell death [137]. MiR-135b-3p exacerbated ferroptosis via inhibiting GPX4 [133].

Long noncoding RNAs (lncRNAs) are functional RNA transcripts exceeding 200 nucleotides in length. They regulate target gene expression at the transcriptional, translational, and post-translational levels by binding to DNA, RNA, and proteins [149]. lncRNAs participate in regulating ferroptosis processes [150–152]. Sun et al. discovered that ferroptosis in HK-2 cells exposed to hypoxia/reoxygenation (H/R) could be inhibited by the lncRNA TUG1. Further functional analysis revealed that TUG1 suppresses ACSL4 expression by interacting with SRSF1 to reduce its mRNA stability, thereby exerting an inhibitory effect on ferroptosis [150]. Increasing evidence suggests that lncRNAs may upregulate downstream gene expression by competing endogenous RNAs (ceRNAs) and sponge miRNAs [153]. LncAABR07025387.1 was shown to function as a ceRNA to sponge miR-205 (downregulating miR-205 expression), consequently enhancing ACSL4 expression and exacerbating ferroptosis I/R [154]. Conversely, several lncRNAs exert ferroptosis-protective effects. For example, the bone marrow mesenchymal stem cell (BMSC)-derived lncRNA Mir9-3hg was reported to inhibit ferroptosis via regulating the pumilio RNA binding family member 2 (Pum2)/peroxiredoxin 6 (PRDX6) axis [155].

Circular RNAs (circRNAs) are a special class of single-stranded non-coding RNAs featuring stable covalent closed-loop structures that resist degradation by RNA nucleases [156]. Zheng et al. constructed a circRNA-miRNA-mRNA regulatory network involving 7 circRNAs, 7 miRNAs, and 4 mRNAs by analyzing differentially expressed genes associated with iron metabolism. Among these, the downregulation of circSnx12 induces iron overload by releasing miR-224-5p to downregulate FTH1 levels [139]. CircRNA1615 regulated the expression of low-density lipoprotein receptor-related protein 6 (LRP6) through sponge adsorption of miR-152-3p to prevent LRP6-mediated autophagy-related ferroptosis [138].

Building upon the detailed elucidation of the core molecular machinery governing ferroptosis—including iron dysregulation, lipid peroxidation, antioxidant failure, and multilayered regulation by non-coding RNAs—it becomes essential to establish a mechanistic bridge linking these fundamental processes to CVD pathophysiology. Ferroptosis represents more than an isolated form of regulated cell death; rather, it functions as a pathophysiological nexus through which metabolic imbalance, oxidative stress, inflammation, and cell fate decisions converge within cardiovascular tissues.

## Ferroptosis in the pathogenesis of different CVDs

CVD typically begins with vascular abnormalities and frequently culminates in heart failure (HF), with the death of terminally differentiated cardiomyocytes representing a key pathological driver. The cardiovascular system is uniquely vulnerable to ferroptotic stress due to its high metabolic demand, elevated oxygen consumption, and dense mitochondrial networks, which together generate substantial reactive oxygen species (ROS). In addition, cardiomyocytes, endothelial cells, and vascular smooth muscle cells are enriched in polyunsaturated fatty acids, rendering their membranes highly susceptible to lipid peroxidation—the defining event of ferroptosis. This vulnerability is further exacerbated by the heart’s central role in iron handling and redox regulation, where disruption of iron homeostasis can rapidly promote ferroptotic injury.

When dysregulated iron metabolism, impaired antioxidant defenses (such as GPX4 inactivation or system Xc⁻ inhibition), and excessive lipid peroxidation converge, ferroptosis contributes directly to cardiovascular disease pathogenesis. In cardiomyocytes, it drives mitochondrial dysfunction and contractile failure, whereas in the vasculature it promotes endothelial dysfunction, vascular remodeling, and inflammation. Importantly, ferroptosis is inherently pro-inflammatory, establishing a self-amplifying pathogenic axis that operates across major cardiovascular diseases. Accordingly, the following sections examine the interplay between ferroptosis and inflammation and its disease-specific roles in atherosclerosis, myocardial ischemia–reperfusion (I/R) injury, cardiomyopathies, heart failure and hypertention, among others.

### Inflammation and ferroptosis

Inflammation represents a fundamental pathological mechanism that pervades virtually all forms of cardiovascular disease, contributing to endothelial dysfunction, vascular remodeling, myocardial injury, and disease progression. Emerging evidence indicates that ferroptosis and inflammation are tightly interconnected through bidirectional regulatory pathways, forming a self-amplifying pathological circuit. Inflammatory signaling can sensitize cardiovascular cells to ferroptosis by disrupting iron homeostasis and antioxidant defenses, whereas ferroptotic cell death amplifies inflammatory responses through the release of lipid peroxidation products and damage-associated molecular patterns. Understanding this reciprocal crosstalk is therefore critical for elucidating how ferroptosis contributes to cardiovascular pathophysiology across diverse disease contexts.

#### The immunogenic nature of ferroptosis

Unlike apoptosis, which proceeds in an immunologically silent manner, ferroptosis is inherently immunogenic. Cells undergoing ferroptosis release damage-associated molecular patterns (DAMPs), including high mobility group box 1 (HMGB1), ATP, and oxidized lipid species, which serve as potent triggers for inflammatory responses [157–159]. These DAMPs are recognized by pattern recognition receptors on immune cells and neighboring tissue cells, initiating cascades of pro-inflammatory signaling. In the cardiovascular context, DAMPs release from ferroptotic cardiomyocytes or endothelial cells activates resident macrophages, recruits circulating monocytes and neutrophils, and stimulates the production of inflammatory cytokines such as tumor necrosis factor-α (TNF-α), interleukin-1β (IL-1β), and interleukin-6 (IL-6). This inflammatory milieu not only exacerbates tissue injury but also creates conditions that promote further ferroptosis in adjacent cells, establishing a vicious cycle of cell death and inflammation.

#### Arachidonic acid metabolism: a molecular bridge linking ferroptosis and inflammation

Arachidonic acid (AA) metabolism represents a critical molecular intersection where ferroptosis and inflammation converge. During cellular stress, AA is released from membrane phospholipids by phospholipase A₂ (PLA2) or phospholipase C (PLC). Subsequently, AA undergoes enzymatic conversion through two principal pathways: the cyclooxygenase (COX) pathway, which generates prostaglandins (PGs), and the lipoxygenase (LOX) pathway, which produces leukotrienes (LTs), lipoxins (LXs), and hydroxyeicosatetraenoic acids (HETEs) [160].

The COX pathway is particularly relevant to the ferroptosis-inflammation axis. COX exists in two isoforms: COX-1, which is constitutively expressed, and COX-2, an inducible enzyme encoded by prostaglandin-endoperoxide synthase 2 (PTGS2). COX-2 plays a dual role in ferroptosis and inflammation. Studies demonstrate that ferroptosis directly upregulates PTGS2 expression, thereby accelerating AA metabolism and promoting the secretion of pro-inflammatory mediators [161]. Conversely, the enzymatic activity of both COX and LOX is directly regulated by cellular lipid hydroperoxide homeostasis [162]. Under normal conditions, the GPX4-GSH antioxidant system maintains low levels of lipid hydroperoxides, which suppresses excessive COX and LOX activity. The cysteine-GSH-GPX4 axis inhibits LOX activation by maintaining a reduced intracellular redox environment [42, 163]. However, when ferroptosis occurs and GPX4 function is compromised, massive accumulation of lipid hydroperoxides stimulates COX and LOX activity. This creates a feed-forward loop wherein ferroptotic cells, laden with oxidized lipids, serve as transcellular donors of AA and oxidized AA derivatives, fueling inflammatory lipid mediator production in neighboring cells.

#### Bidirectional regulation between ferroptosis and inflammation

The relationship between ferroptosis and inflammation is characterized by reciprocal reinforcement, with each process potentiating the other. On one hand, ferroptosis triggers inflammation through multiple mechanisms. The release of oxidized phospholipids and their breakdown products acts as danger signals that activate inflammatory pathways. DAMPs released from ferroptotic cells engage Toll-like receptors and other pattern recognition receptors, initiating NF-κB signaling and cytokine production [157–159]. Dar et al. demonstrated that bacterial-secreted LOXs can induce phospholipid oxidation in host epithelial cells, triggering ferroptosis [164]. The ferroptosis-derived oxidized lipids subsequently activate immune responses, creating a pathogen-induced inflammation-ferroptosis cascade.

On the other hand, inflammatory mediators can directly induce or sensitize cells to ferroptosis. Multiple inflammatory cytokines, including TNF-α, prostaglandin E2 (PGE2), IL-1β, IL-6, and IL-1, have been shown to modulate GPX4 expression and activity [165]. TNF-α treatment, for instance, causes sustained downregulation of GPX4, rendering cells more susceptible to ferroptosis [166]. Furthermore, LOX-derived pro-inflammatory metabolites, including leukotrienes (LTB4, LTC4, LTD4, and LTE4), HETEs, and oxoeicosanoids, not only recruit and activate immune cells but also create an oxidative microenvironment that facilitates lipid peroxidation and ferroptosis [159]. This bidirectional regulation establishes a self-perpetuating cycle where initial ferroptotic events trigger inflammatory responses, which in turn promote additional rounds of ferroptosis in surrounding tissue.

#### Ferroptosis-inflammation interactions in cardiovascular pathology

The ferroptosis-inflammation crosstalk manifests across multiple cardiovascular disease contexts, though its specific features vary depending on the pathological condition. In ischemia–reperfusion injury, which occurs during myocardial infarction, stroke, or following heart transplantation, ferroptosis of endothelial cells and cardiomyocytes releases DAMPs that initiate robust inflammatory responses [167]. Under ischemic conditions, ferroptosis in transplanted endothelial cells enhances neutrophil recruitment to damaged cardiac tissue through upregulation of adhesion molecules and chemokine gradients [168]. This inflammatory cell infiltration amplifies tissue injury through release of proteolytic enzymes, reactive oxygen species, and additional inflammatory mediators.

Zhang et al. provided direct evidence for the ferroptosis-inflammation connection in cerebrovascular injury, demonstrating that ferroptosis induces inflammation following intracerebral hemorrhage (ICH) in rats. Treatment with the ferroptosis inhibitor Fer-1 significantly reduced both ROS levels and concentrations of inflammatory mediators such as IL-1β and TNF-α, while simultaneously improving neurological function [169]. These findings suggest that ferroptosis acts upstream of inflammatory activation in certain injury contexts, and that ferroptosis inhibition can break the pathological cycle.

In atherosclerosis, the ferroptosis-inflammation nexus contributes to plaque progression and instability. Oxidized low-density lipoprotein (ox-LDL) induces ferroptosis in macrophages and endothelial cells, releasing pro-inflammatory lipid mediators that recruit additional immune cells and perpetuate vascular inflammation. In heart failure, chronic low-grade inflammation creates a tissue environment conducive to ferroptosis through sustained oxidative stress and iron dysregulation, while ferroptotic cardiomyocyte death releases inflammatory signals that drive adverse cardiac remodeling.

The intimate connection between ferroptosis and inflammation positions this crosstalk as a central pathogenic mechanism in cardiovascular disease. Therapeutic strategies targeting either ferroptosis or inflammation may exert beneficial effects through disruption of this reinforcing cycle. Understanding the molecular details of ferroptosis-inflammation interactions in specific cardiovascular pathologies provides a foundation for developing more targeted and effective interventions, as will be explored in the disease-specific sections that follow.

### Atherosclerosis

Atherosclerosis (AS) is the most common disease of the cardiovascular system, providing the pathological basis for a range of cardiovascular diseases [170, 171]. AS is a lipid disorder characterized by lipid accumulation in the arterial wall and the formation of foam cells as its defining features [172–175]. The development of atherosclerotic or fibrous plaques within the vascular endothelial cells (ECs) leads to wall stiffening, lumen narrowing, and reduced elasticity, ultimately causing ischemic changes in the corresponding organs.

Recent studies indicate an association between elevated iron levels and atherosclerosis [176]. Stadler et al. detected iron deposition in coronary plaques [177]. Gustafsson's research demonstrated that coronary plaques in symptomatic atherosclerosis patients exhibit higher iron concentrations and a greater risk of plaque rupture [178]. Vinchi et al. [179] demonstrated that elevated systemic iron levels trigger arterial iron deposition via NTBI, further inducing vascular dysfunction and plaque formation. NTBI, a hallmark of iron overload, increases vascular endothelial permeability and adhesion by promoting oxidation and mediating the release of vascular endothelial growth factor (VEGF); elevated VEGF levels are associated with atherosclerosis [180, 181]. Furthermore, elevated NTBI levels stimulate monocyte chemoattractant protein (MCP-1) expression in ECs and vascular smooth muscle cells (VSMCs), thereby attracting monocytes/macrophages and accelerating foam cell formation and plaque evolution [181, 182].

Inhibiting ferroptosis slows atherosclerotic progression in ApoE-deficient mice [183]. By reducing the availability of iron through systemic iron chelation therapy or surgical intervention like venotomy, the progression of AS can be slowed. Restricting dietary iron intake is another strategy that has been shown to reduce lesion size and increase plaque stability in AS models [184]. This approach helps decrease the oxidative stress and lipid peroxidation associated with AS.

As established in Section “ The canonical system Xc-/GSH/GPX4 pathway”, system Xc-/GSH/GPX4 is the primary defense against lipid peroxidation. In the context of atherosclerosis, this defense system is frequently compromised. For instance, studies have shown that GPX4 expression is negatively correlated with the severity of AS. Zhou et al. [185] examined 40 human coronary artery specimens and found a negative correlation between the severity of AS and the expression of GPX4-an enzyme critical in defending against ferroptosis that inhibits macrophage foam cell formation by interacting with HDL. Bai et al. [186] induced AS in ApoE-deficient mice using a high-fat diet and treated them with a ferroptosis inhibitor ferrostatin-1 (Fer-1). Fer-1 up-regulates the expression of SLC7A11, GPX4, and endothelial nitric oxide synthase (eNOS) while downregulating the expression of adhesion molecules, thereby reducing lipid peroxidation and endothelial dysfunction in aortic endothelial cells and alleviating atherosclerosis. These findings underscore the significance of GPX4 in AS and its potential as a therapeutic target. Liu's [187] research in the ApoE-deficient mice indicates that reducing SLC7A11 and GPX4 levels, leading to altered intracellular GSH and iron concentrations in foam cells and decreased macrophage migratory capacity from the intima, thereby promoting the development of AS. Using the ferroptosis inhibitor Fer-1 mitigated AS lesions and foam cell formation both in vivo and in vitro. Yang’s study [188] demonstrated that Fer-1 activates AMPK signaling, leading to the upregulation of FTH, GPX4, and scavenger receptor class B type I (SCARB1)—a critical mediator of cholesterol efflux. This activation reduces intracellular iron content and lipid accumulation in macrophages, thereby slowing the progression of atherosclerosis.

### Myocardial infarction and ischemia–reperfusion injury

Myocardial infarction (MI) represents a secondary pathological change of atherosclerosis, characterized by irreversible myocardial necrosis resulting from prolonged hypoxia/ischemia. It constitutes the fundamental cause of cardiomyocyte death, ventricular remodeling, and heart failure [189]. TFR1 serves as a key regulator of ferroptosis, Das De et al. demonstrated a significant negative correlation between TFR1 saturation and MI [190]. Park's [191] research revealed that myocardial infarction can induce a reduction in GPX4, another key regulator of ferroptosis. Proteomic analysis of mouse cardiac tissue after myocardial infarction induced by left anterior descending ligation demonstrated that the protein levels of the ferroptosis inhibitor GPX4 were significantly downregulated in the early and mid-stages of myocardial infarction. This may render cardiomyocytes susceptible to ferroptosis under low GSH conditions.

Baba et al. [192] demonstrated that ferroptosis is a major cause of cell death in myocardial infarction zones. Modulating LOOHs and iron via the the mechanistic target of rapamycin (mTOR) can inhibit ferroptosis in adult mouse cardiomyocytes. Gao et al. reported that the inhibition of lncRNA Gm47283 can mitigate the effects of myocardial infarction through elevating the expression of glutathione peroxidase 4 (GPX4) [193]. Furthermore, Li et al. [138] discovered that circular RNA 1615 (circRNA1615) can regulate the expression of Low-density lipoprotein receptor-related protein 6 (LRP6) through sponge adsorption of miR-152-3p, thereby preventing LRP6-mediated autophagy-related ferroptosis in cardiomyocytes. This finding indicates that circRNA1615 can control the pathological progression of myocardial infarction by modulating ferroptosis-related pathways.

Revascularization is an effective strategy for salvaging ischemic myocardium in myocardial infarction [194, 195], while restoration of blood flow to the ischemic myocardium paradoxically exacerbates cardiac tissue damage, a phenomenon known as myocardial ischemia–reperfusion (I/R) injury [196, 197]. For a long time, cell atrophy, necrosis, and autophagy-associated cell death have been considered key factors in I/R pathology [198]. However, this process is increasingly recognized as being associated with ferroptosis. A study [199] demonstrated that the presence of intra-myocardial hemorrhage (IMH) in patients with ST-segment elevation myocardial infarction who underwent interventional therapy reperfusion for the first time was related to residual myocardial iron at follow-up, suggesting a link between iron metabolism and reperfusion injury. In mouse models of myocardial ischemia–reperfusion, substantial ferritin accumulation along the scar areas indicates that excess iron can lead to cardiomyocyte death through ferroptosis [200]. Studies have found that the oxidative burst associated with reperfusion is accompanied by lipid peroxidation [201] and elevated intracellular iron levels [202]. Fang et al.'s research demonstrated that ferroptosis occurs in a mouse model of myocardial I/R injury [203]. Stamenkovic et al. [204] demonstrated that oxidized phosphatidylcholine production increases during IR injury, causing extensive cell death via ferroptosis. Gao et al. further observed increased expression of ferroptosis marker ACSL4 in rat hearts following I/R or hypoxia-reoxygenation, demonstrating that ferroptosis can be suppressed by iron chelation or inhibition of glutamine metabolism [205].

Ferroptosis occurs during specific periods. Tang et al. [206] observed no significant change in ferroptosis hallmarks (ACSL4, GPX4, iron, and malondialdehyde) during severe myocardial ischemia. However, as reperfusion time extended, ACSL4, iron, and malondialdehyde levels increased, while GPX4 levels decreased, suggesting that ferroptosis predominantly occurs during the reperfusion phase rather than the ischemic phase. Reperfusion in ischemic tissue generates massive ROS, triggering oxidative bursts that further mediate I/R injury [207]. Ferroptosis intervention exerts positive effects only on reperfusion injury, while showing no significant impact on ischemic injury [208]. This indicates that the relationship between the reperfusion phase and ferroptosis—characterized by lipid peroxidation—is markedly stronger than that observed during the ischemic phase.

Ferroptosis induces I/R injury by triggering endoplasmic reticulum stress (ERS). ERS is a pathological condition characterized by the massive accumulation of misfolded proteins within the endoplasmic reticulum. It induces apoptosis by binding to the pro-apoptotic protein PUMA through the ATF4-CHOP pathway [209]. Studies reveal that ferroptosis inducers can trigger the unfolded protein response (UPR), subsequently activating the PERK/EIF2α/ATF4/CHOP pathway to initiate ERS [210]. Furthermore, ferroptosis can activate ERS by promoting the system Xc-. Dixon et al. demonstrated that CHOP-mediated ERS plays a crucial role in rat I/R injury [211]. ROS generated during ferroptosis can also trigger ERS as a cellular response to endoplasmic reticulum dysfunction [212]. These studies suggest that ferroptosis-induced ERS serves as a bridge between ferroptosis and I/R injury.

### Cardiomyopathies

Iron accumulation in cardiomyocytes can lead to iron overload cardiomyopathy [213]. Researchers generated cardiomyocyte-specific FTH1-deficient mice to study the specific effects of iron on cardiomyopathy. These mice exhibited altered cardiac iron homeostasis and developed mild cardiomyopathy with aging, while the Fer-1 protected them from hypertrophic cardiomyopathy. Furthermore, SLC7A11 overexpression in FTH1-deficient cardiomyocytes restored GSH production and significantly reduced ferroptosis and cardiomyopathy onset [214]. In cardiomyopathy, decreased cellular FTH1 levels may disrupt iron metabolism, downregulate SLC7A11 and GPX4 expression, and cause an imbalance in GSH synthesis and consumption, ultimately leading to ferroptosis and cellular damage. SLC7A11 is a target gene of p53. p53 sensitizes tumor cells to ferroptosis by transcriptionally suppressing SLC7A11, and inhibition of p53 abolishes this effect [104]. Consequently, p53 knockout mice exhibit preserved cardiac function in anthracycline-induced and I/R-induced cardiomyopathy [215–217].

Metabolic cardiomyopathy is characterized by abnormalities within cardiomyocytes, such as excessive triacylglycerol accumulation and lipid toxicity injury, which can lead to diastolic dysfunction [218]. In mice models, GPX4-a critical antioxidant enzyme of ferroptosis, deletion has been shown to exacerbate myocardial hypertrophy induced by a high-fat and high-sugar diet, primarily due to increased mitochondrial lipid peroxidation [219]. The expression of GPX is reduced in myocardial tissue from diabetic cardiomyopathy rats [220]. Conversely, the overexpression of GPX4 in mitochondria can protect the heart from damage induced by streptozotocin [221]. This protective effect is further supported by observations that GPX4 levels are diminished in cardiomyocytes of patients with diabetic heart disease [219], suggesting therapies of ferroptosis antioxidants have shown promise in alleviating diabetes-induced oxidative stress and cardiac dysfunction. For instance, antioxidants such as coenzyme Q_10_ and vitamin E, which act as ferroptosis inhibitors, can mitigate oxidative stress and improve cardiac diastolic function in diabetic hearts [222]. In a study [223] involving mice with type 2 diabetes, sulforaphane was used to inhibit ferroptosis, thereby preventing diabetes-related cardiac-inflammatory responses, oxidative damage, and hypertrophy.

As a prominent chemotherapeutic agent, Adriamycin is widely utilized in clinical settings; however, its potential cardiotoxicity can lead to cardiomyopathy and calcium metabolism disturbances. Doxorubicin (DOX) has the inherent ability to chelate Fe3 +, forming a doxorubicin-Fe3 + complex that catalyzes the formation of hydroxyl radicals (OH-). These radicals, in conjunction with superoxide anions and doxorubicin itself, can cause DNA damage [224], and disrupt protein and lipid metabolism, ultimately resulting in cardiac tissue harm. This process is similar to the mechanism of ferroptosis, where ROS generation is intensified, causing oxidative damage that can exacerbate adriamycin-induced cardiotoxicity. Research has revealed that in mice with DOX-induced cardiomyopathy, heme oxygenase-1 (HO-1) levels are significantly upregulated, leading to heme degradation and the release of free iron from the heart [225]. Administration of the ferroptosis inhibitors Fer-1 and DXZ significantly reduced mortality, whereas inhibitors of apoptosis, necrosis, and autophagy did not improve the condition of the mice [226].

### Heart failure

Heart failure (HF) represents the ultimate clinical outcome for most primary CVDs, where cardiomyocytes undergo various pathological changes like apoptosis, autophagy, and necrosis. Iron deficiency is the most prevalent nutritional disorder in humans, affecting up to 75% of heart failure patients [227]. Elevated levels of labile iron pools and lipid peroxides in HF model rats indicate that ferroptosis is directly implicated in HF [228]. The expression of FHC [229] protein is significantly downregulated in mice with HF which leads to a substantial release of free iron, creating a state of excess iron within the cell. The formation of NTBI in the body allows this free iron to wreak havoc on mitochondria, causing cellular and tissue damage [230]. This damage inhibits calcium (Ca^2+^) influx, disrupting the excitation–contraction coupling of cardiomyocytes and leading to heart failure. Liu et al. [231] identified characteristic mitochondrial structural changes(as we discussed in Section “ Mitochondrial dysfunction and structural changes in ferroptosis”) associated with ferroptosis in cardiomyocytes of rats with heart failure, including mitochondrial contraction and increased mitochondrial membrane density. In murine hypertrophic hearts models, the upregulated expression of mitochondrial protein sideroflexin 1 (SFXN1)mediates mitochondrial iron overload by transferring iron from the cytoplasm into the mitochondria, leading to excessive mitochondrial ROS production and increased lipid peroxidation, which triggers ferroptosis [232]. These collective findings substantiate the notion that ferroptosis is a significant mechanism in the progression of heart failure.

Yang et al. [233] and Koleini et al. [234] identified a key role for ferroptosis in DOX-induced HF. DOX induces intracellular accumulation of oxidized phospholipids, which upregulates HO-1 expression via Nrf2, leading to cardiac heme degradation and release of free iron. This triggers ferroptosis in terminally differentiated cardiomyocytes, ultimately leading to heart failure. Inhibiting ferroptosis or downregulating HO-1 significantly reduces DOX-induced cardiac injury and heart failure. Reduced FTH1 levels in cells cause iron metabolism abnormalities and ROS accumulation, thereby inducing ferroptosis. Chen et al. [235]demonstrated that in stress-overloaded HF rats, puerarin inhibits cardiomyocyte ferroptosis and improves cardiac function by inducing FTH1 and GPX4 production while reducing ROS generation. Thus, modulating FTH1 levels via upstream regulatory pathways may represent a key therapeutic strategy for HF.

### Hypertension

Hypertension is a major risk factor for CVD, arising from vascular remodeling, endothelial dysfunction, and increased vasoconstriction [236]. In angiotensin II (Ang II)-induced hypertensive mice, elevated iron levels in cardiac tissue were observed, accompanied by reduced GPX4 and Nrf2 expression and increased malondialdehyde (MDA) [237]. Treatment with the ferroptosis inhibitor Fer-1 reduced myocardial hypertrophy and pathological remodeling [237], underscoring the role of ferroptosis in hypertensive cardiac injury. Ferroptosis has also been shown to promote the phenotypic switch of vascular smooth muscle cells (VSMCs) under high hydrostatic pressure, a process closely linked to hypertension pathophysiology [238].

Clinical and genetic data further support the connection between iron overload and hypertension. A cross-sectional study revealed a positive correlation between serum ferritin levels and hypertension prevalence [239]. Mutations in the major histocompatibility complex class I-like transmembrane protein, specifically the H63D mutation in the HFE gene, and mutations in the hemojuvelin (HJV) gene, both associated with systemic iron overload, have been linked to elevated hypertension risk [240, 241]. Furthermore, Yang et al. [242] discovered that, in hypertensive rats, brain tissues exhibited increased iron and lipid peroxidation levels compared to those with normal blood pressure.

GPX4 inactivation is another key driver. Jin et al. [243]found reduced GPX4 expression in the aortic media of hypertensive patients. Their work further showed that exposing VSMCs to high hydrostatic pressure (200 mmHg) depleted cystathionine γ-lyase/hydrogen sulfide, impairing GSH synthesis, lowering GPX4, and triggering ferroptosis. In cardiomyocytes, Zhang et al. [244] demonstrated that SLC7A11 overexpression suppressed Ang II–induced hypertrophy by reducing levels of prostaglandin-endoperoxide synthase 2 (PTGS2), MDA, and ROS, suggesting SLC7A11 as a promising therapeutic target in hypertensive heart disease.

Oxidative stress, a hallmark of ferroptosis, plays a central role in hypertension-related vascular injury. Elevated ROS exacerbate endothelial damage, stimulate VSMC proliferation, and drive vascular remodeling, thereby raising peripheral resistance and blood pressure [245]. Farooqui et al. [246] showed that Nrf2 inhibition increased oxidative stress and aggravated hypertension in mice, whereas Nrf2 activation alleviated hypertension during Ang II infusion, highlighting its therapeutic relevance.Mitochondrial dysfunction, including impaired energy metabolism, increased mtROS, and mtDNA damage, amplifies ferroptosis and worsens endothelial injury [247, 248]. Loss of the mitochondrial deacetylase Sirtuin 3 (Sirt3) compromises mtROS clearance, enhances ferroptosis, and promotes vascular inflammation and remodeling. In hypertensive patients, reduced Sirt3 expression is associated with endothelial dysfunction, vascular thickening, and end-organ damage [249].

### Other CVDs and ferroptosis

#### Pulmonary hypertension(PH) and ferroptosis

Pulmonary arterial hypertension (PAH) is a vascular disease characterized by remodeling of pulmonary arterioles, elevated pulmonary arterial pressure, and right ventricular hypertrophy [250, 251]. PAH is a form of PH primarily influenced by iron metabolism [252]. Nearly 40% of idiopathic PAH patients have been reported to be associated with iron deficiency and reduced exercise capacity [253]. Intravenous iron supplementation improves quality of life and exercise capacity in PAH patients, potentially resulting from enhanced oxygen transport in skeletal muscle [252, 254, 255]. Ferroptosis participates in the pathogenesis and progression of PH. Iron deficiency specific to pulmonary arterial smooth muscle cells correlates with pulmonary vascular dysfunction and PH progression in mice [256]. Fe^2^⁺ accumulation and reduced GPX4 levels were observed in monocrotaline-induced PH rats, and Fer-1 improved vascular remodeling and right ventricular function by inhibiting ferroptosis [257]. In a chronic hypoxia-induced PH rat model, treatment with the iron chelator DFO attenuated pulmonary vascular remodeling [258].

#### AAD and ferroptosis

Aortic aneurysm and dissection (AAD) are severe vascular diseases characterized by aortic medial degeneration [259, 260]. Ferroptosis is crucial for the onset and progression of AAD. Research indicates that the iron content in the aortas of AD patients is significantly elevated, along with increased levels of transferrin receptor, ferritin, and 4-hydroxynonenal—a product of lipid peroxidation. Concurrently, there is a significant downregulation of ferroptosis inhibitory genes such as SLC7A11, FSP1, and GPX4 [261]. In Stanford type A aortic dissection patients, TfR and HMOX1 expression is upregulated, while SLC7A11 and GPX4 expression are downregulated [261]. Administration of lipstatin-1 (Lip-1) in mouse models improves AAD incidence and mortality by mitigating medial degeneration through ferroptosis inhibition [261]. The histone methyltransferase inhibitor BRD4770 inhibited aortic dilation by preventing ferroptosis and lipid peroxidation, thereby reducing the incidence and mortality of BAPN-induced aortic dissection [262]. Therefore, targeting ferroptosis represents a potential therapeutic strategy for treating AAD.

#### Stroke and ferroptosis

Following ischemic or hemorrhagic stroke, neurons can undergo ferroptosis due to elevated extracellular glutamate concentrations inhibiting system Xc^−^ function [263]. This process can be blocked by the ferroptosis inhibitor ferrostatin-1 [7]. In organotypic hippocampal slice cultures (OHSCs), ferrostatin-1 can prevent neuronal death and reduce hemoglobin-induced iron accumulation, suggesting a pathogenic role for ferroptosis in cerebral hemorrhage [264]. Neurons isolated from ischemic stroke mouse models demonstrated significantly reduced GSH levels and elevated lipid peroxidation, indicating ferroptosis in these cells. Furthermore, upregulating GPX4 expression helped protect hippocampal neurons from ferroptosis in a gerbil cerebral ischemia model [265].

## Ferroptosis as a therapeutic target in CVD

Targeting the core pathological drivers of ferroptosis—including dysregulated iron metabolism, uncontrolled lipid peroxidation, impairment of the GPX4 antioxidant axis, and additional regulatory pathways such as FSP1–CoQ_10_, GCH1–BH4, and DHODH—represents a promising strategy to suppress ferroptosis in cardiovascular disease. By modulating these critical molecular processes, it is possible to restore redox balance, protect cardiomyocytes from lethal oxidative stress, and ultimately attenuate the progression of myocardial injury and other ferroptosis-related cardiovascular pathologies.

### Iron chelators

The term “ferroptosis” originates from the rescue effect of the iron chelator deferoxamine. Clinically, deferoxamine (DFO), deferiprone (DFP), and deferasirox (DFX) are commonly used iron chelators [266]. Reports indicate that DFO can prevent iron overload in various animal disease models, including neurodegeneration, I/R-induced injury, and hemorrhagic stroke [203, 267–270]. Side effects of DFO include anemia and edema [271]. DFP is an effective oral hydrophilic iron chelator developed as an alternative to DFO [272]. DFP exhibits fewer side effects [273] compared to DFO and can cross the blood–brain barrier (BBB) [274]. DFP has been used to improve motor function in patients with Friedreich's ataxia [275]. The only iron chelator currently approved by FDA for therapeutic use is dexrazoxane (DXZ), which prevents doxorubicin (DOX)-induced cardiotoxicity by chelating DOX-induced mitochondrial iron [276]. Ciclopirox olamine, currently used to treat dermatic fungal infections, also inhibits the activity of iron-dependent ribonucleotide reductase and is employed as an iron chelator [277, 278].

Gao et al. [205] reported that TF receptor-TfR1 mediated cellular uptake of iron-bound transferrin is essential for ferroptosis, and that RNA interference (RNAi)-mediated suppression of TfR1 effectively blocks ferroptosis. Feng et al. [279] identified TfR1 as a marker for ferroptosis and investigated the TFR1-specific antibody 3F3-FMA as a potential therapeutic approach. Horonchik and colleagues found that the TFR1 inhibitor ferristatin (also known as NSC306711) induces TfR1 degradation to suppress iron uptake [280].

DMT1 is an NTBI transporter that transports Fe^2^⁺ to the duodenum during the TF cycle [281]. DMT1 inhibitors (including ebselen [282], pyridine dithiocarbamate [282] and benzyl isothiourea [283]) reduce iron-induced injury by decreasing DMT1-mediated cellular uptake of NTBI, suggesting DMT1 may be a potential target for regulating iron-related diseases.

Targeting iron export to regulate ferroptosis represents a promising strategy. FPN is the only known iron exporter in the body [284], primarily regulated by the liver-derived peptide, hepcidin [285]. Hepcidin agonists such as minihepcidins [286] and FPN inhibitors like viti-2763 [286] can modulate ferroptosis by targeting the hepcidin-FPN axis. Some small molecules promote intracellular iron accumulation by directly upregulating hepcidin expression, leading to ferroptosis. Endogenous inducers of hepcidin, such as the cytokine OSM (encoded by the OSM gene) [287] can also promote ferroptosis. Hepcidin antagonists (e.g., PRS-080 [286], NOX-H94 [288], and LY2787106 [289]) may exert their ferroptosis-inhibitory effects by reducing intracellular iron levels.

Ferritinophagy [290] refers to the process whereby NCOA4 binds to iron-containing ferritin under iron-deficient conditions, leading to lysosomal degradation of ferritin and release of iron into LIPs. Ferritinophagy can induce iron overload and lipid peroxidation, thereby triggering ferroptosis [291, 292]. Fang et al. [293] recently discovered a novel compound named 9a that effectively inhibits ferritinophagy-induced ferroptosis by competitively binding to NCOA4 and disrupting its interaction with FTH1.

The enzyme HO-1, encoded by the HMOX1 gene, catalyzes the conversion of heme into carbon monoxide, biliverdin, and free iron (as we discussed in Section “ NRF2 -HO-1 pathway”). Elevated HMOX1 expression leads to increased iron concentrations and high levels of ferrous iron [294], which can accelerate the Fenton reaction when radical scavengers are insufficient [295]. Targeting HO-1 has been considered a viable strategy for treating cardiovascular diseases. Fang et al. [203]demonstrated that inhibiting HO-1 prevents apoptosis-induced cardiomyopathy in mice. Vreman et al. [296] discovered that metalloporphyrins, used to treat neonatal jaundice, act as HO-1 inhibitors but exhibit adverse effects such as phototoxicity and/or off-target effects. Azalanstat [297] represents a safer HO-1 inhibitor. However, the efficacy of these drugs for iron-related diseases requires further validation through research.

### Lipid peroxidation inhibitors

Given the pivotal role of lipid metabolism in ferroptosis, targeting these pathways may offer novel strategies for inhibiting ferroptosis. Approaches to intervene in lipid metabolic pathways include LOX inhibitors, radical-trapping antioxidants (RTAs, which scavenge lipid hydroxyl radicals), ACSL4 inhibitors, and deuterated PUFAs/MUFAs (by reducing PUFA-containing phospholipids).

LOXs are a class of non-heme iron-containing peroxidases [298] that regulate ferroptosis by mediating phospholipid(PL) oxidation [299]. Six LOX subtypes are expressed in humans [300]: ALOX5, ALOX12, ALOX12B, ALOX15, ALOX15B, and ALOXE3. Inhibition of ALOX5/12 has been shown to suppress ferroptosis. Zileuton, an FDA-approved oral medication for treating asthma, is an ALOX5 inhibitor that exerts neuroprotective effects by blocking ALOX5-mediated glutamate toxicity and ferroptosis in HT22 cells, a mouse hippocampal cell line [301]. ML355, an effective selective ALOX12 inhibitor [302], demonstrates organ protection by reducing ALOX12-induced thrombosis-mediated injury [303, 304]. Studies have demonstrated that ALOX15 exacerbates I/R-induced brain injury [305] and ischemia-induced myocardial injury [306] by inducing PL-PUFA peroxidation and ferroptosis, suggesting the therapeutic potential of targeting ALOX15 for these I/R-induced tissue injuries. Cai et al. [307] demonstrated that ML351, a specific ALOX15 inhibitor, reduces ferroptosis in I/R-induced cardiac injury.

PL peroxidation triggered by the generation and propagation of lipid radicals is a hallmark of ferroptosis, and this process can be blocked using radical-trapping antioxidants (RTAs)-a class of lipid chain‒breaking antioxidants. Fer-1 is one such RTA that reduces tissue damage caused by iron toxicity by scavenging radicals, and it has been shown to prolong mouse survival in disease models [203, 308, 309]. However, due to its metabolic instability and poor pharmacokinetics, Fer-1has been used only as a research tool to study various iron-related processes and cannot serve as a clinical drug. Scientists are developing RTA drugs with improved metabolic and kinetic properties [310]. Liproxstatin-1 (Lip-1) is also an effective RTA [54], exhibiting superior pharmacological activity in vivo for inhibiting ferroptosis compared to Fer-1. However, Lip-1 concurrently inhibits the enzymatic activity of CYP2D6 (a member of the cytochrome P450 superfamily of drug-metabolizing enzymes), which may render Lip-1 unsuitable for clinical trials beyond its role in suppressing ferroptosis [54]. Diarylamines are also RTAs commonly used to reduce the occurrence of autooxidation in petroleum derivatives [311]. Their derivatives—phenothiazines and phenoxazines—exhibit ferroptosis-inhibitory effects in mouse embryonic fibroblasts [312]. Notably, the catalytic activity of effective RTAs like Fer-1 and Lip-1 requires relatively high temperatures [313], whereas each phenoxazine molecule can capture > 2 peroxyl radicals at moderate temperatures, opening prospects for developing RTA-based ferroptosis inhibitors [36]. Edaravone, clinically approved for treating acute ischemic stroke and amyotrophic lateral sclerosis [314], is also an RTA that has been demonstrated to prevent ferroptosis under various pathological conditions [315].

ACSL4 involves in the metabolism of PL-PUFAs (such as AA) as a unique and important isoenzyme. Deletion or inhibition of ACSL4 prevents PL-PUFAs from entering the cell membrane, thereby inhibiting ferroptosis [37, 316]. Thiazolidinediones (TZDs), drugs used to treat adult-onset type 2 diabetes, have been found to inhibit ACSL4 [37].

Deuterated PUFAs and MUFAs can also inhibit ACSL4 by reducing PL-PUFAs. PUFAs deuterated at the bis-allylic position (D-PUFAs) were found to suppress ferroptosis in cellular and animal models of Parkinson's disease and Friedreich's ataxia [317, 318]. Furthermore, studies indicate that exogenous MUFAs can suppress ferroptosis in an ACSL3-dependent manner by specifically reducing the accumulation of lipid ROS in the plasma membrane and displacing PUFAs from cell locations [319].

### GPX4 pathway activators

The system Xc^−^/GSH/GPX4 pathway plays a crucial role in suppressing ferroptosis mediated by lipid peroxidation reactions [13]. Activating this pathway enhances cellular resistance to ferroptosis.

N-acetylcysteine (NAC) [320], used to treat acetyl-p-aminophenol overdose, is an antioxidant that can improve neurodegenerative symptoms by increasing cysteine levels and promoting the synthesis of γ-glutamylcysteine and GSH [321]. N-acetylcysteine amide (NACA) has been developed and demonstrated antioxidant activity in multiple preclinical models, exhibiting higher membrane permeability [322] and bioavailability (NACA: 67% vs. NAC: 15%) [323] compared to NAC.

Selenium (Se) supplementation may help prevent tissue damage and diseases associated with iron toxicity. Se is essential for the active site of GPX4 [324]—selenocysteine [325]. Delivering Se to cultured neurons via ionic selenite (SeO₃^2⁻^) increases GPX4 transcription and inhibits hemin- or homocysteine-induced ferroptosis [326]. Animal models revealed that Tat SelPep, a selenocysteine-containing peptide capable of crossing the blood–brain barrier, improves functional recovery in mice after hemorrhagic and ischemic strokes by blocking ferroptosis [326].

The neurotransmitter dopamine exerts numerous physiological effects, with levodopa and dopamine receptor agonists clinically employed to treat Parkinson's disease and various cardiovascular disorders. Research indicates dopamine may serve as a promising therapeutic candidate for mitigating ferroptosis-related tissue damage and diseases, as well as certain neurodegenerative conditions. Non-oxidized dopamine effectively suppresses erastin-induced ferroptosis by stabilizing GPX4 [327]. Li et al. identified eight novel potential GPX4 activators and determined that compound 1d4 is the most potent allosteric activator of GPX4, effectively inhibiting ferroptosis in HT-1080 fibrosarcoma cells [328].

### FSP1 and other novel pathway activators

The FSP1-CoQ_10_-NAD(P)H pathway and glutamine hydrolysis pathway represent potential targets for regulating ferroptosis in CVD. Additionally, NRF2 responds to cellular oxidative stress by activating the transcription of genes involved in redox reactions. Therefore, targeting the KEAP1-NRF2 axis may serve as a viable strategy for modulating ferroptosis.

The FSP1-CoQ_10_-NAD(P)H pathway: FSP1 gene was initially identified as pro-apoptotic gene [329], but it has also been characterized as an anti-ferroptotic gene. Through its redox enzyme activity, FSP1 reduces CoQ_10_ to uniquinol (CoQ_10_H₂) [60, 61]. Fang et al. [330] discovered that the diphenylbutene derivative compound 3f acts as a ferroptosis inhibitor. This compound prevents ischemic stroke in rats by increasing FSP1 protein levels. Novel drugs that upregulate FSP1 expression may be used to treat iron-related diseases.

Glutaminolysis Pathway: Cellular uptake of the amino acid glutamine (Gln) is primarily mediated by specific transporters such as SLC38A1 and SLC1A5. Following cellular uptake, intracellular glutamine is converted to glutamate by glutamine synthase (GLS). This glutamate is then further catalyzed to α-ketoglutarate via deamination mediated by glutamate dehydrogenase (GLUD1) or transamination mediated by transaminases [331]. Given that this reaction promotes ferroptosis by inducing lipid peroxidation accumulation, blocking the glutaminolysis pathway has been proposed as a potential therapeutic approach for ferroptosis-induced tissue injury. In ex vivo models, compound 968 (a GLS inhibitor) has been demonstrated to effectively prevent I/R-induced cardiac injury [205].

Targeting the p62-KEAP1-NRF2 signaling pathway. In response to oxidative stress, p62 activates NRF2 by directly binding to its ubiquitin ligase partner KEAP1 during the oxidative stress response; consequently, NRF2 translocates to the nucleus and regulates cellular redox homeostasis by modulating the expression of target genes, including several genes encoding enzymes involved in GSH synthesis and iron homeostasis [117]. Thus, NRF2 signaling plays a crucial role in regulating iron homeostasis. Sitagliptin, a selective inhibitor of dipeptidyl peptidase 4 used to treat type 2 diabetes, has been shown to suppress ROS-induced tissue damage by promoting Nrf2 translocation to the nucleus [332].

### Alternative strategies targeting ferroptosis

Although numerous ferroptosis-targeting agents have been investigated (in Table 2 we list drugs and potential agents with anti-ferroptosis activity related to CVDs), their properties—low solubility, high metabolic clearance, poor cellular permeability, and systemic toxicity—render them unsuitable as therapeutic compounds. Even though some of these drugs have advanced to clinical trials (as indicated in Table 2), these limitations severely constrain their clinical development progress. Consequently, multi-target ferroptosis modulators and nanomaterial technologies have been explored to achieve more effective in vivo targeting of ferroptosis.

**Table 2:** NCT (national clinical trial) drugs or potential agents with anti-ferroptosis activity in CVDs

**Agent**	**Type**	**Mechanism**	**National clinical trial(NCT)**		**Refs**
Compound 9a	Iron metabolism	Perturbation of NCOA4- ferritin heavy chain 1 interaction Fth1	N/A		[293]
Deferoxamine (DFO)		Chelation of iron	Ischemic stroke	NCT00870883	[333]
			Hypotension	NCT00870883	
			Aneurysmal subarachnoid hemorrhage	NCT04566991	
Deferiprone (DFP)		Chelation of iron	Acute myocardial infarction Type 1	NCT05604131	[334]
			Stroke	NCT05111821	
Deferasirox (DFX)		Chelation of iron	Sickle cell disease	NCT05392101	[335, 336]
Dexrazoxane (DXZ)		Chelation of iron	NCT04997291 NCT03589729		[203]
Resveratrol		Reducing Fe^2+^ and TfR1, increasing FTH1, GPX4	N/A		[337]
Atorvastatin		Regulating SMAD7/hepcidin pathway	N/A		[338]
Baicalein	Lipid metabolism	ALOX12 inhibitor; ALOX12/ALOX15 inhibitor; ACSL4 inhibitor	NCT03830684		[339–341]
Gossypol Acetic Acid		Reducing lipid peroxidation, decreasing ACSL4 and NrF2, increasing GPX4.	N/A		[342]
rapamycin		Inhibiting mTOR	N/A		[343]
Xanthohumol		Inhibiting lipid peroxidation/Chelation of iron	N/A		[344]
apoferritin	Reductive-oxidative	increasing GPX4，reducing iron pool and lipid peroxides	N/A		[345]
Britanin		Upregulating GPX4 through activation of the AMPK/GSK-3β/Nrf2 signaling pathway	N/A		[346]
Carvedilol		upregulating GPX4, *FTH1*, and *FTL1* levels	N/A		[347]
Dapagliﬂozin		Regulating MAPK Pathway	N/A		[348]
Dexmedetomidine		Regulating AMPK Pathway	N/A		[349]
Edaravone		RTA	Myocardial infarction	NCT00265239	[350, 351]
			Cerebral infarction	NCT00200356	
			Acute ischemic stroke	NCT02430350	
Etomidate		Regulating Nrf2/HO-1 Pathway	N/A		[352]
Ferrostatin-1		RTA	N/A		[167]
Ferulic acid		decreasing GSH/GSSG, upregulating AMPK α2 and GPX4	N/A		[353]
Histochrome		Upregulating Nrf2，GPX4	N/A		[354]
Liproxstatin-1		RTA	N/A		[355]
N-acetylcysteine (NAC)		GSH synthesis regulator	Vascular cognitive impairment no dementia	NCT03306979	[356–358]
			Chronic thromboembolic pulmonary hypertension	NCT04081012	
Naringenin		Regulating Nrf2/System xc-/GPX4	N/A		[359]
Propofol		Regulating P53 signaling pathway	N/A		[360]
6-Gingerol	Chinese herbal	Regulating Nrf2/HO-1 Pathway	N/A		[361]
Shenmai Injection		Regulating Nrf2/GPX4 pathway	N/A		[362]

#### Multi-target ferroptosis inhibitors

Glycyrrhizin is a natural antioxidant extracted from glycyrrhiza root [363], exhibiting anti-inflammatory, anti-fibrotic, and antiviral properties that protect the liver [320, 364, 365]. Research by Wang et al. [366]revealed that glycyrrhizin can prevent ferroptosis in animal models by regulating iron levels, the GSH/GPX4 pathway, and the NRF2/HO-1/HMGB1 pathway.

Baicalin [367] has long been used as an antibacterial and antiviral agent. Its protective effects against oxidative damage, achieved by inhibiting iron-catalyzed Fenton reactions, were reported as early as the late twentieth century [368]. Baicalin has been demonstrated as a specific antagonist of ALOX12, preventing I/R-induced myocardial injury in mice [339]. Leyen et al. [340] further revealed that baicalin also prevents I/R-induced brain injury by inhibiting ALOX12/15. Also, baicalin was shown to prevent ferroptosis in erastin-treated PANC1 cells (a human pancreatic cancer cell line) by reducing ferrous iron, inhibiting GSH depletion, and suppressing GPX4 degradation, thereby inhibiting lipid peroxidation [369]. These results indicate that certain natural products exert their inhibitory effects on ferroptosis through multiple targets.

Puerarin, an isoflavone extracted from *Pueraria lobata*, has been increasingly investigated for its cardioprotective effects. Research indicates that puerarin enhances antioxidant defense by regulating ferritin, activates the Nrf2 signaling pathway through upregulation of GPX4 and SLC7A11, and effectively alleviates iron overload and lipid peroxidation in heart failure models, thereby protecting cardiomyocytes from damage [228].

#### Novel technologies

RNA-based therapies refer to a novel class of treatments that utilize ribonucleic acid (RNA) molecules to prevent or treat diseases. Rather than directly altering genetic sequences, they exert their effects by regulating gene expression or delivering functional RNA molecules. Although no clinical trials have yet utilized RNA-based technologies to investigate targeted therapies for iron overload, RNA therapeutics offer unique opportunities in CVD. Messenger RNA (mRNA) therapies can supply functional proteins that are otherwise absent or defective, with potential applications in ischemic heart disease and inherited cardiomyopathies. RNA interference (RNAi) and antisense oligonucleotides (ASOs) enable selective silencing of maladaptive genes, such as those driving lipid metabolism dysregulation (silencing ACSL4 or activating GPX4 expression), inflammation, or myocardial remodeling.

Drug delivery nanoparticles (DDNs) are nanoscale carriers (1–1000 nm) designed to encapsulate, adsorb, or conjugate drugs, enabling targeted, controlled, and safe delivery of therapeutic agents to specific sites in the body. Unlike conventional drug formulations (e.g., tablets, injections), DDNs address key limitations of traditional drug delivery—such as poor solubility, nonspecific tissue distribution, and high systemic toxicity—by leveraging their nanoscale size and modifiable properties. Two types of nanoparticles have been reported to inhibit ferroptosis. Carboxyl-modified polystyrene nanoparticles (CPS) effectively suppress ferroptosis by reducing TFEB (transcription factor EB)-mediated cellular ROS [370]. Another is DEF-HCC-PEG, a deferoxamine-binding nanoparticle that protects cells from both ferroptosis and senescence [371].

**Fig. 4:**
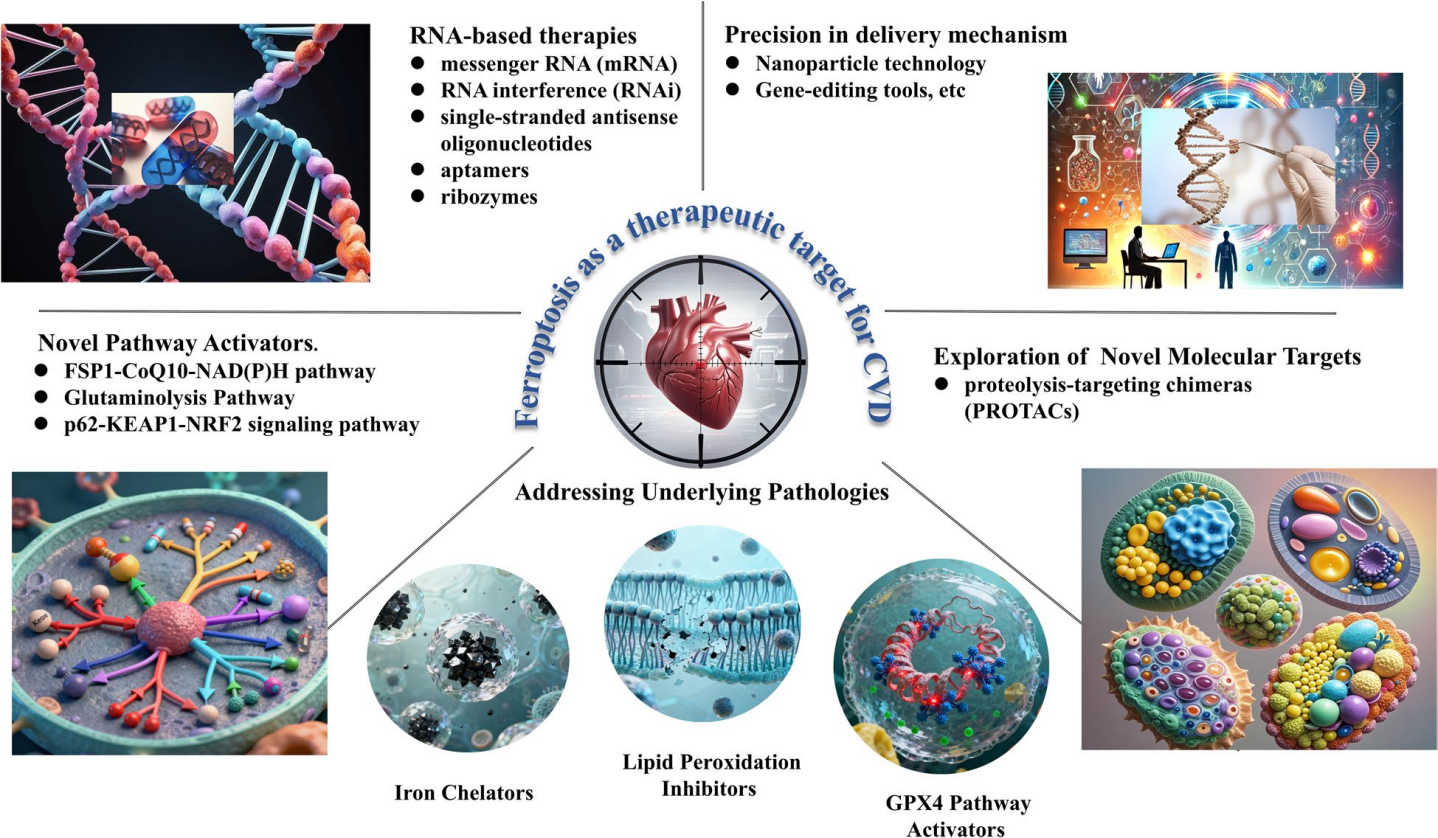
Ferroptosis as a therapeutic target for CVD. Targeting the core pathological drivers of ferroptosis—including iron chelators, lipid peroxidation inhibitors, and GPX4 pathway activators—has shown promise in mitigating oxidative injury and cell death in CVD. Novel regulatory pathway activators, such as the FSP1–CoQ_10_–NAD(P)H axis, glutaminolysis pathway, p62–KEAP1–NRF2, represent additional mechanisms that suppress ferroptosis. Beyond small molecules, RNA-based therapies (messenger RNA, RNA interference, antisense oligonucleotides, aptamers, and ribozymes) offer gene-level modulation to restore protective proteins or silence deleterious genes, particularly relevant to ischemic and inherited cardiomyopathies. Precision delivery technologies, including nanoparticle platforms and gene-editing tools, enhance therapeutic specificity and overcome the limitations of traditional drug delivery. Finally, the exploration of novel molecular targets such as proteolysis-targeting chimeras (PROTACs) enables selective degradation of pro-ferroptotic proteins, expanding the landscape of ferroptosis-based interventions for cardiovascular therapy. *The five small illustrative images in the lower panel of this figure were generated using an AI-assisted image generation tool for conceptual visualization purposes only. These images were subsequently manually cropped, adjusted, and assembled by the authors using Microsoft PowerPoint to create the final figure. No experimental data, quantitative results, or biological images were generated, modified, or interpreted using AI tools, and the use of AI did not influence data analysis, scientific interpretation, or conclusions of the study*

Additionally, novel technologies such as proteolysis-targeting chimeras (PROTACs), gene editing, peptides, and protein therapeutics have been applied to develop ferroptosis-targeting drugs. Artificial intelligence has been employed to rapidly identify lead compounds extracted from plants and microorganisms, significantly accelerating the discovery of novel ferroptosis-targeting therapeutics [36] (Fig. 4).

### Challenges in therapeutic translation

#### Targeting specificity & off-target toxicity

A major challenge is achieving cardiomyocyte-specific ferroptosis modulation without harming other cells. Systemic delivery of ferroptosis inhibitors can affect iron-rich, metabolically active tissues beyond the heart, reflecting poor target selectivity [372, 373]. For example, untargeted ferroptosis suppression may inadvertently impair immune cell function or viability. The myocardium’s close proximity to liver and skeletal muscle (which also have high iron utilization) further complicates specific drug targeting. Consequently, current ferroptosis inhibitors risk off-target toxicities and narrow therapeutic indices. To overcome this, tissue-specific delivery strategies (e.g. cardiotropic nanoparticles or peptide-guided prodrugs) are being explored to confine drug action to the heart, thereby improving safety and efficacy [374].

#### Pharmacokinetics & drug-delivery barriers

Many ferroptosis inhibitors are lipophilic small molecules that suffer from rapid metabolism and clearance, yielding subtherapeutic bioavailability in cardiac tissue. For instance, the prototype ferrostatin-1 is quickly inactivated in vivo due to an ester bond, underscoring inherent stability problems [375]. Achieving sufficient drug concentration in the myocardium is difficult, as reactive oxygen scavengers tend to have short plasma half-lives and may not accumulate at injury sites [376]. Nanocarrier delivery can protect such drugs but faces host defenses: particles are often opsonized and cleared by the mononuclear phagocyte system, reducing cardiac uptake. Furthermore, large biologics (e.g. siRNA against ferroptosis mediators) struggle with cell entry and enzymatic degradation. Emerging solutions include encapsulating ferroptosis inhibitors in long-circulating liposomes or polymeric nanoparticles to prolong circulation and enhance myocardial deposition [376], as well as designing more stable analogues with improved pharmacokinetic profiles [375].

#### Timing & therapeutic window

Ferroptotic cell death in acute cardiac injury occurs within a narrow time frame, making the timing of intervention critical. In myocardial ischemia/reperfusion (I/R) injury, studies show that ferroptosis is triggered mainly upon reperfusion (the reoxygenation phase) rather than during ischemia per se [208]. This implies that administering an anti-ferroptotic agent too early (during ischemia) may be futile, whereas delayed treatment misses the peak window of lipid peroxidation damage. Similarly, in heart failure models ferroptosis may contribute at specific disease stages (e.g. during acute decompensation) rather than continuously. Ill-timed therapy could therefore yield minimal benefit. Moreover, the transient burst of oxidative injury after events like myocardial infarction limits the period during which ferroptosis inhibitors are effective. Optimizing the therapeutic window is an active area of research. One approach is the development of triggerable drug delivery systems (e.g. ROS-responsive carriers) that release inhibitors precisely during oxidative bursts, ensuring ferroptosis is counteracted at the most critical moments.

#### Physiological roles of iron & ROS homeostasis

Iron and ROS are indispensable for normal cardiovascular physiology, so chronically suppressing ferroptosis can disrupt homeostasis. Long-term iron chelation or continuous GPX4 activation might protect against cell death but at the cost of interfering with essential processes like erythropoiesis and mitochondrial respiration [352]. In heart failure patients, iron deficiency is known to impair cardiac energetics (mitochondrial function) and exacerbate symptoms [377], highlighting the risk of overzealous iron removal. Similarly, a basal level of ROS is involved in cell signaling and defense; indiscriminate antioxidant therapies could blunt these beneficial pathways. Thus, completely shutting down ferroptotic pathways may have systemic side effects, including anemia, immunosuppression, or metabolic derangements. To address this, researchers are investigating intermittent or tissue-targeted ferroptosis inhibition, aiming to preserve physiological iron/ROS functions. Careful monitoring of iron indices and combining treatments with iron supplementation (when appropriate) are potential strategies to strike a safe balance.

#### Lack of validated clinical biomarkers & imaging tools

Another translational hurdle is the absence of robust biomarkers to identify ferroptosis in patients and guide therapy. At present, ferroptosis is mainly confirmed by experimental measures (e.g. malondialdehyde or 4-HNE levels for lipid peroxidation, or immunostaining of ACSL4/GPX4 in tissues), which are not standardized for clinical use. Blood tests like serum iron or ferritin are nonspecific, and no FDA-approved companion diagnostic exists to detect “ferroptotic activity” in cardiovascular disease. This makes it difficult to know which patients would benefit from ferroptosis-targeted drugs or to monitor drug efficacy in vivo. Imaging modalities to visualize ferroptosis (for example, novel MRI contrast agents or PET tracers for iron-driven lipid peroxidation) remain in early development. The inability to easily distinguish ferroptosis from other forms of cell death in patients is a significant barrier [378]. Accordingly, current research is focused on discovering specific ferroptosis biomarkers and imaging probes. For instance, recent work on a transferrin receptor-1 antibody suggests a possible ferroptosis detector [279]. Validating such tools in clinical settings will be key to patient stratification and real-time monitoring of anti-ferroptotic therapies.

#### Heterogeneity across CVD entities & comorbidities

The extent and importance of ferroptosis can differ widely between cardiovascular conditions. For example, in atherosclerosis, lipid peroxidation and iron deposition in plaques contribute to lesion instability [379], whereas in acute myocardial infarction ferroptosis plays a direct role in cardiomyocyte cell death during reperfusion. In contrast, in inflammatory heart diseases (e.g. myocarditis or septic cardiomyopathy) pyroptosis and other immune-cell driven death pathways may dominate, with ferroptosis playing a smaller or as-yet undefined role. Indeed, some CVDs (such as myocarditis, dilated cardiomyopathy, or aortic aneurysm) have seen little investigation into ferroptotic mechanisms [380]. Patient comorbidities also modulate ferroptosis susceptibility; for instance, diabetes mellitus elevates oxidative stress and iron dysregulation, potentially exacerbating ferroptosis in diabetic hearts and accelerating cardiomyopathy. This heterogeneity means a one-size-fits-all ferroptosis therapy is unlikely to work across all CVDs. The solution lies in precision medicine: ongoing studies aim to identify which subsets of patients or disease states would benefit most from ferroptosis modulation. Tailoring treatments (or combination therapies) to disease context – for example, anti-ferroptotic drugs for post-MI or high-iron phenotypes, versus other pathways for pure inflammatory cardiomyopathies – will maximize efficacy and minimize unnecessary interventions.

#### Regulatory & manufacturing hurdles

Finally, translating ferroptosis-targeting strategies into real-world therapies faces practical development challenges. Many proposed interventions (e.g. nanoparticle-delivered siRNAs, mRNA therapeutics boosting antioxidant defenses, or PROTACs targeting ferroptosis regulators) are complex biologics that do not fit the traditional small-molecule pipeline. Scaling up their production with consistent quality is non-trivial. For instance, manufacturing a lipid nanoparticle formulation or a recombinant protein therapy requires specialized Good Manufacturing Practice (GMP) facilities and rigorous chemistry, manufacturing, and controls (CMC) protocols [381]. Ensuring batch-to-batch reproducibility, sterility, and stability of such formulations can be challenging and costly. Regulatory agencies have limited experience with ferroptosis inhibitors, so demonstrating safety in the context of long-term CVD treatment (as opposed to oncology, where ferroptosis inducers are also being tested) may demand extensive preclinical data. High development costs and unclear approval pathways can deter pharmaceutical investment in this nascent field. To facilitate clinical translation, researchers and industry are collaborating to streamline manufacturing processes for novel therapeutics. Standardizing nanoparticle production methods, improving PROTAC pharmacokinetics, and reducing costs (potentially via automation and better formulation chemistry) are priorities. Clear regulatory guidelines and early engagement with agencies are also being pursued to ensure that promising ferroptosis-based CVD therapies can enter clinical trials and, ultimately, patient care.

Despite these formidable challenges, the rapid evolution of ferroptosis science and the promising preclinical efficacy of ferroptosis-targeted interventions justify continued translational efforts. As novel therapeutic candidates advance through early-phase trials and nanomedicine platforms mature, overcoming these barriers will require close collaboration among basic researchers, clinicians, industry partners, and regulatory bodies. Importantly, successful clinical translation of ferroptosis-targeted therapies will hinge not only on developing effective and safe drugs, but also on our ability to identify patients most likely to benefit and to monitor therapeutic responses in real time. Such precision medicine approaches are contingent upon robust diagnostic and prognostic tools that can guide patient stratification, define optimal therapeutic windows, and serve as pharmacodynamic endpoints. In the following section, we examine how ferroptosis-related biomarkers and imaging modalities are being developed to guide cardiovascular care.

## Diagnostic & prognostic: from discovery to clinical application

The persistently high incidence and mortality rates of CVD underscore an urgent need for more effective methods for early diagnosis and prognosis assessment. Several biomarkers currently used in clinical practice, such as C-reactive protein (CRP), creatine kinase-MB (CK-MB), and growth differentiation factor-15, have limited applicability due to confounding factors including patient age, genetic background, cardiac comorbidities, and lifestyle [382, 383]. Given that ferroptosis serves as a significant pathophysiological mechanism across the spectrum of CVD, its associated molecular and imaging signatures represent a promising frontier for developing innovative biomarkers with high specificity and sensitivity. While current research is actively exploring a spectrum of ferroptosis-related candidates, none has yet achieved routine clinical use for this specific purpose [384]. The following sections categorize these emerging biomarkers and outline the rigorous path from laboratory discovery to clinical validation.

### Potential ferroptosis biomarkers

Key candidates span circulating biochemical markers, non-coding RNAs, extracellular vesicle (EV) signatures, and imaging surrogates. Each category is at a different stage of development, and translating these findings from discovery to practice requires a phased validation pipeline.

#### Iron-handling proteins and composite indices

Dysregulated iron metabolism is a prerequisite for ferroptosis, making serum iron markers attractive candidates. Ferritin and transferrin saturation (TSAT) are routinely measured indices of body iron. In heart failure (HF), they are used to define iron deficiency when ferritin is < 100 ng/mL or ferritin is 100–299 ng/mL with TSAT < 20% [385]. Such deficits are common in approximately 50% of HF patients and are associated with worse functional status and outcomes. In fact, current guidelines recommend using ferritin/TSAT to guide intravenous iron therapy in patients with heart failure with reduced ejection fraction (HFrEF), demonstrating the established clinical utility of these biomarkers for risk and treatment decision-making [386].

Conversely, elevated ferritin or TSAT may indicate iron overload and heightened oxidative injury. However, for other conditions like atherosclerotic disease and stroke, observational and Mendelian-randomization evidence is mixed, with studies showing positive, inverse, or null associations depending on the specific endpoint. Hepcidin, the master regulator of iron egress, may further refine risk stratification, as low hepcidin levels have been associated with a higher incidence of HF and mortality, potentially reflecting a state of functional iron deficiency [227, 387]. While these protein biomarkers are in advanced analytical validation with standardized clinical assays, their interpretation in the context of ferroptosis is complicated by inflammatory influences, as ferritin is also an acute-phase reactant. Composite indices, such as ferritin adjusted for hepcidin or soluble transferrin receptor, are being studied to better link systemic iron handling with ferroptotic susceptibility.

#### Circulating peroxidized lipids

Lipid peroxidation products in the blood, such as malondialdehyde (MDA), 4-hydroxynonenal (4-HNE), and isoprostanes, directly reflect the oxidative membrane damage central to ferroptosis [388]. Elevated plasma levels of these aldehydes have been linked to cardiac pathology; for instance, 4-HNE accumulates in failing myocardium and impairs RNA regulatory mechanisms [389, 390]. As classical oxidative stress footprints, they correlate with CVD severity. Standardized assays, such as mass spectrometry for isoprostanes, exist and have demonstrated independent risk associations in heart disease [391, 392]. However, their specificity for ferroptosis versus generalized oxidative injury remains a key uncertainty. Furthermore, pre-analytical instability and confounding by diet or comorbidities pose significant challenges. Further validation is needed to determine if circulating lipid peroxides can serve as reliable surrogates for ferroptosis rather than just broad markers of oxidative stress.

#### GPX4 as a pharmacodynamic biomarker

GPX4 is the central enzymatic defender against lipid peroxidation and ferroptosis. At present, GPX4 is primarily a translational/therapeutic target: experimental GPX4 up-regulation or ferroptosis inhibition reduces lesion burden in animal models, suggesting its potential therapeutic utility in the future. Human clinical studies measuring GPX4 activity as a cardiovascular disease risk predictor remain scarce. Most research focuses on tissue expression changes rather than prospectively using plasma GPX4 activity as a criterion for ferroptosis occurrence. If such therapies advance to clinical trials, GPX4 activity or downstream lipid-peroxide readouts would serve as reasonable pharmacodynamic endpoints.

#### Non-coding RNAs and extracellular vesicle signatures

Regulatory non-coding RNAs (ncRNAs) have emerged as fine-tuners of ferroptotic pathways and hold immense potential as circulating biomarkers due to their ideal stability and detectability in body fluids [393]. Multiple microRNAs (miRs) modulate ferroptosis in cardiomyocytes; for example, miR-135b-3p directly downregulates GPX4 and exacerbates myocardial ischemia–reperfusion injury [132], whereas miR-30d and miR-190a-5p confer protection by targeting pro-ferroptotic enzymes or upregulating ferritin heavy chain [135, 136].

Beyond preclinical models, clinical studies have highlighted the diagnostic and prognostic potential of circulating ncRNAs in CVD. For instance, Li et al. found that elevated circulating miRNA-203 could distinguish patients with acute ST-segment elevation myocardial infarction from healthy controls with high accuracy (Area Under the Curve [AUC] = 0.912) [394]. Similarly, Xiao et al. demonstrated that serum miR-30d levels were strongly prognostic in acute HF patients, with an AUC of 0.806 for predicting mortality, outperforming several traditional markers [395]. Long non-coding RNAs (lncRNAs) and circular RNAs (circRNAs) show similar promise. The lncRNA NRF exhibited superior diagnostic value (AUC = 0.937) compared to N-terminal pro-brain natriuretic peptide (AUC = 0.72) in distinguishing acute myocardial infarction patients with HF from those without [396]. Likewise, lncRNA KCNQ1OT1 and a panel of circRNAs (DNAJC6, TMEM56, MBOAT2) have demonstrated high diagnostic and prognostic value for coronary artery disease [397] and hypertrophic cardiomyopathy [398], respectively.

Notably, these RNA biomarkers often circulate packaged in Extracellular Vehicles (EVs), such as exosomes, which protect them from degradation. A striking example is the lncRNA UCA1, which is enriched in exosomes after myocardial infarction and was shown to reduce ferroptotic cell death in cardiac tissue [399]. Also, lncRNA SEMA5A-IT1 carried by small extracellular vesicles after cardiopulmonary bypass protected cardiomyocytes from I/R injury [400].These underscores the dual role of ncRNAs as both mediators and indicators of ferroptosis. This category is largely in the discovery phase; while numerous candidates have been identified, concerns about consistency and reproducibility remain. Further research is needed to definitively link many of these CVD-associated ncRNAs to ferroptosis processes, and larger patient cohorts are required to validate their biomarker potential.

In addition to their cargo, the EVs themselves may carry signatures of ferroptosis. Cells undergoing ferroptosis may release EVs with distinctive membrane markers or protein cargo, such as the enzyme ACSL4, which propagates lipid peroxidation [401]. This biomarker category remains at an early experimental stage, with challenges including the heterogeneity of circulating vesicles and the low abundance of disease-specific EVs.

#### Imaging surrogates of ferroptosis

Non-invasive imaging offers a unique means to monitor ferroptosis-associated changes in vivo. Iron-sensitive magnetic resonance imaging (MRI) sequences, such as T2* mapping, can quantify tissue iron deposition and are already established for detecting myocardial iron overload in conditions like thalassemia [402]. In acute cardiac injury, T2-weighted cardiac MRI can map intramyocardial hemorrhage, an iron-rich hallmark of reperfusion injury that correlates strongly with adverse outcomes [403]. This positions MRI iron mapping as a clinical surrogate for tissue environments prone to ferroptosis. Alongside MRI, positron emission tomography (PET) is being explored for imaging oxidative stress. Novel PET tracers, such as the ^18^F-FPBT probe, can visualize cardiac oxidative burden in preclinical models of cardiotoxicity [404]. While T2 MRI is already in clinical use, ROS-specific PET tracers remain in preclinical validation. A key challenge for both is specificity, as the imaging signal is an indirect indicator of ferroptosis.

### The biomarker development pipeline: from discovery to clinical validation

For any of the aforementioned candidates to become a trusted clinical tool, it must navigate a rigorous, multi-stage validation process [384]. The first step is the discovery phase, which involves identifying candidate markers and demonstrating initial associations with ferroptosis in cellular models or small patient sets. This is followed by analytical validation, confirming that the marker can be measured accurately and reproducibly, for example, by establishing assay precision for lipid peroxides or RNA quantification. The third stage is clinical qualification, where the biomarker’s correlation with disease outcomes, its added prognostic value, and its consistent performance are verified in larger, independent patient cohorts. Finally, upon sufficient evidence, regulatory acceptance can be sought through endorsement by agencies or inclusion in clinical guidelines, allowing the biomarker to be used in routine practice or as an endpoint in drug development. This road is often long, and many promising ferroptosis markers remain bottlenecked in preclinical stages due to limited specificity or insufficient validation data. Table 3 summarizes the current stage of each biomarker category and key barriers to its clinical implementation.

**Table 3:** Developmental Status and Key Challenges of Potential Ferroptosis Biomarkers

**Biomarker Category**	**Stage of Development**	**Key Challenges**	**Refs**
**Circulating peroxidized lipids & Aldehydes (e.g., 4-HNE, isoprostanes)**	Analytical validation	Difficulty in distinguishing ferroptosis-specific signals from general oxidative stress.; Challenges in assay standardization, including analyte stability and pre-analytical variability.	[384, 388]
**Iron-handling proteins & hepcidin (e.g., Ferritin, TSAT, Hepcidin)**	Clinical use (iron status indices) – Context unqualified for ferroptosis	Systemic levels are heavily influenced by confounding factors such as inflammation and iron deficiency states; Requirement for developing composite indices and risk stratification models to link systemic iron status to ferroptotic risk.	[385]
**Non-coding RNAs (e.g., miRNA, lncRNA, circRNA)**	Discovery/early validation	Low reproducibility reported for many candidates identified in preclinical studies; Low abundance in circulation and technical challenges in normalization for quantitative assays; Requires development of robust detection methods and large-scale clinical validation.	[399, 405]
**Extracellular Vesicle (EV) Signatures(Surface proteins & protein cargo)**	Discovery (experimental concept)	Technical challenges in isolating specific EV subpopulations and detecting low-abundance signals; Absence of standardized, consensus protocols for EV detection and cargo analysis; Lack of unique, validated EV markers that are specific to ferroptotic cells.	[406]
Imaging Surrogates(e.g., MRI T2*, PET-ROS probes)	Clinical (MRI iron mapping); Preclinical (PET)	MRI: While clinically validated for imaging iron overload, it lacks specificity for ferroptosis-mediated cell death processes. PET: Novel ROS-sensitive tracers show promise in preclinical models but require extensive human trials and regulatory approval for clinical application.	[384, 404]

### Future directions and concluding remarks

To accelerate the translation of ferroptosis biomarkers into cardiovascular care, future efforts should focus on several key areas. First, developing multi-biomarker panels that integrate complementary markers (such as lipid peroxides, iron indices, and ferroptosis-regulating miRNAs) could improve specificity and predictive power over any single marker. Second, longitudinal clinical studies are essential to track ferroptosis biomarkers over time and in response to interventions, which will help solidify causal links between biomarker dynamics and patient outcomes. Third, technological innovation in high-sensitivity detection methods, such as point-of-care tests or novel PET tracers, is needed to enable early and accessible detection. Finally, proactive engagement with regulatory agencies and education of clinicians will be crucial for the smooth incorporation of validated biomarkers into risk stratification models and for guiding future ferroptosis-targeted therapies in cardiovascular disease.

## Conclusion and prospects

Ferroptosis, distinguished from apoptosis and necrosis by its dependence on iron metabolism and lipid peroxidation, has rapidly emerged as a pivotal form of regulated cell death in cardiovascular medicine, and fundamentally reshaped current concepts of cardiovascular pathophysiology. Over the past decade, accumulating experimental evidence has implicated ferroptosis as a convergent mechanism underlying cardiomyocyte death, vascular remodeling, and inflammation-driven tissue injury across a wide spectrum of CVDs. In multiple preclinical models, pharmacological inhibition of ferroptosis preserves myocardial and vascular function in settings such as ischemic injury, heart failure, and atherosclerosis, positioning ferroptosis as both a central disease driver and a potential therapeutic target. Nevertheless, despite these advances, effective clinical translation remains elusive, and no therapies specifically targeting ferroptosis have yet been approved for cardiovascular indications.

A major barrier to clinical implementation lies in our limited ability to monitor and precisely modulate ferroptosis in humans. Two fundamental challenges predominate. First, the spatiotemporal dynamics of ferroptosis in vivo remain poorly defined. It is unclear at which stages of disease progression—ranging from early atherogenesis to acute myocardial infarction or chronic heart failure—ferroptosis is initiated, how its magnitude evolves, and which cell populations, including cardiomyocytes, endothelial cells, or infiltrating immune cells, contribute most critically to pathology. Second, there is a lack of clinically applicable tools for diagnosis and intervention. Current approaches are largely restricted to experimental settings, while available inhibitors, such as non-specific antioxidants or iron chelators, exhibit limited specificity and raise safety concerns related to off-target effects and disruption of systemic iron homeostasis.

Addressing these challenges will require integrated, human-centric strategies that combine mechanistic insight with technological innovation. Longitudinal cohort studies incorporating non-invasive imaging and serial biomarker profiling will be essential to delineate ferroptosis dynamics across disease stages. Techniques such as iron-sensitive T2* magnetic resonance imaging, coupled with circulating multi-omics analyses, may help identify critical therapeutic windows. concurrently, single-cell and spatial transcriptomic analyses of human cardiovascular tissues will be crucial for resolving cellular heterogeneity and defining cell type–specific contributions. These observations can then be interrogated in advanced human-relevant platforms, including patient-derived iPSC-based cardiac organoids and microfluidic organ-on-a-chip systems, to dissect ferroptosis-associated signaling networks in a controlled environment.

In parallel with mechanistic studies, the development of clinically viable tools is imperative. Therapeutic efforts should move beyond broad-spectrum agents toward highly specific modulators, including structure-guided inhibitors of core regulators such as GPX4, emerging modalities such as PROTAC-based approaches to selectively degrade pro-ferroptotic proteins, and targeted nanomedicine systems for site-specific delivery. On the diagnostic side, the establishment of a composite ferroptosis activity score—integrating lipid peroxidation products, iron metabolism indices, and ferroptosis-associated non-coding RNAs through machine learning frameworks—could enable patient stratification, disease monitoring, and prediction of therapeutic response.

In summary, ferroptosis represents not merely an additional mode of cell death but a conceptual framework that links metabolic dysregulation, oxidative injury, and inflammation in cardiovascular disease. By overcoming current limitations in in vivo assessment and targeted intervention, future research may enable the translation of ferroptosis-based strategies into clinical practice, advancing precision medicine approaches and offering new avenues to mitigate the global burden of CVD.

## Data Availability

The data that support the findings of this study are available from the corresponding author upon reasonable request.

## References

[1] Ga M, V F, Cjl M, Ga R. Global Burden of Cardiovascular Diseases and Risks, 1990–2022. J Am Coll Cardiol [Internet]. 2023;82(25). Available from: https://pubmed.ncbi.nlm.nih.gov/38092509/, 10.1016/j.jacc.2023.11.007. Cited 31 Oct 2025. 10.1016/j.jacc.2023.11.007 PMC7615984 38092509

[2] Tsurusaki S, Kizana E. Mechanisms and therapeutic potential of multiple forms of cell death in myocardial ischemia-reperfusion injury. Int J Mol Sci. 2024;25(24):13492. 10.3390/ijms252413492. 39769255 10.3390/ijms252413492 PMC11728078

[3] Cai K, Jiang H, Zou Y, Song C, Cao K, Chen S, et al. Programmed death of cardiomyocytes in cardiovascular disease and new therapeutic approaches. Pharmacol Res. 2024;206:107281. 10.1016/j.phrs.2024.107281. 38942341 10.1016/j.phrs.2024.107281

[4] Galluzzi L, Vitale I, Aaronson SA, et al. Molecular mechanisms of cell death: recommendations of the Nomenclature Committee on Cell Death 2018 [J]. Cell Death Differ. 2018;25(3):486–541. 29362479 10.1038/s41418-017-0012-4 PMC5864239

[5] Chiong M, Wang ZV, Pedrozo Z, Cao DJ, Troncoso R, Ibacache M, et al. Cardiomyocyte death: mechanisms and translational implications. Cell Death Dis. 2011;2(12):e244. 10.1038/cddis.2011.130. 22190003 10.1038/cddis.2011.130 PMC3252742

[6] Katsiki N, Tziomalos K, Chatzizisis Y, Elisaf M, Hatzitolios AI. Effect of HMG-CoA reductase inhibitors on vascular cell apoptosis: beneficial or detrimental? Atherosclerosis. 2010;211(1):9–14. 10.1016/j.atherosclerosis.2009.12.028. 20060525 10.1016/j.atherosclerosis.2009.12.028

[7] Sj D, Km L, Mr L, R S, Em Z, Ce G, et al. Ferroptosis: an iron-dependent form of nonapoptotic cell death. Cell [Internet]. 2012;149(5). Available from: https://pubmed.ncbi.nlm.nih.gov/22632970/, 10.1016/j.cell.2012.03.042. Cited 15 Sept 2025. 10.1016/j.cell.2012.03.042 PMC3367386 22632970

[8] Guo Y, Lu C, Hu K, Cai C, Wang W. Ferroptosis in cardiovascular diseases: current status, challenges, and future perspectives. Biomolecules. 2022;12(3):390. 10.3390/biom12030390. 35327582 10.3390/biom12030390 PMC8945958

[9] Zhao Y, Linkermann A, Takahashi M, Li Q, Zhou X. Ferroptosis in cardiovascular disease: regulatory mechanisms and therapeutic implications. Eur Heart J. 2025;46(33):3247–60. 10.1093/eurheartj/ehaf374. 40464746 10.1093/eurheartj/ehaf374

[10] Fernández-Acosta R, Vintea I, Koeken I, Hassannia B, Vanden Berghe T. Harnessing ferroptosis for precision oncology: challenges and prospects. BMC Biol. 2025;23(1):57. 10.1186/s12915-025-02154-6. 39988655 10.1186/s12915-025-02154-6 PMC11849278

[11] Wang Y, Lv MN, Zhao WJ. Research on ferroptosis as a therapeutic target for the treatment of neurodegenerative diseases. Ageing Res Rev. 2023;91:102035. 10.1016/j.arr.2023.102035. 37619619 10.1016/j.arr.2023.102035

[12] Wang S, Guo Q, Zhou L, Xia X. Ferroptosis: a double-edged sword. Cell Death Discov. 2024;10(1):265. 10.1038/s41420-024-02037-9. 38816377 10.1038/s41420-024-02037-9 PMC11139933

[13] Zhang DD. Ironing out the details of ferroptosis. Nat Cell Biol. 2024;26(9):1386–93. 10.1038/s41556-024-01361-7. 38429476 10.1038/s41556-024-01361-7

[14] Chen H, Attieh ZK, Su T, Syed BA, Gao H, Alaeddine RM, et al. Hephaestin is a ferroxidase that maintains partial activity in sex-linked anemia mice. Blood. 2004;103(10):3933–9. 10.1182/blood-2003-09-3139. 14751926 10.1182/blood-2003-09-3139

[15] Mayle KM, Le AM, Kamei DT. The intracellular trafficking pathway of transferrin. Biochim Biophys Acta. 2012;1820(3):264–81. 10.1016/j.bbagen.2011.09.009. 21968002 10.1016/j.bbagen.2011.09.009 PMC3288267

[16] Levy JE, Jin O, Fujiwara Y, Kuo F, Andrews NC. Transferrin receptor is necessary for development of erythrocytes and the nervous system. Nat Genet. 1999;21(4):396–9. 10.1038/7727. 10192390 10.1038/7727

[17] Stoyanovsky DA, Tyurina YY, Shrivastava I, Bahar I, Tyurin VA, Protchenko O, et al. Iron catalysis of lipid peroxidation in ferroptosis: Regulated enzymatic or random free radical reaction? Free Radic Biol Med. 2019;133:153–61. 10.1016/j.freeradbiomed.2018.09.008. 30217775 10.1016/j.freeradbiomed.2018.09.008 PMC6555767

[18] Li Y, Huang X, Wang J, Huang R, Wan D. Regulation of iron homeostasis and related diseases. Mediators Inflamm. 2020;2020:6062094. 10.1155/2020/6062094. 32454791 10.1155/2020/6062094 PMC7212278

[19] Galaris D, Barbouti A, Pantopoulos K. Iron homeostasis and oxidative stress: an intimate relationship. Biochim Biophys Acta Mol Cell Res. 2019;1866(12):118535. 10.1016/j.bbamcr.2019.118535. 31446062 10.1016/j.bbamcr.2019.118535

[20] Lakhal-Littleton S, Wolna M, Carr CA, Miller JJJ, Christian HC, Ball V, et al. Cardiac ferroportin regulates cellular iron homeostasis and is important for cardiac function. Proc Natl Acad Sci U S A. 2015;112(10):3164–9. 10.1073/pnas.1422373112. 25713362 10.1073/pnas.1422373112 PMC4364209

[21] Fang J, Kong B, Shuai W, Xiao Z, Dai C, Qin T, et al. Ferroportin-mediated ferroptosis involved in new-onset atrial fibrillation with LPS-induced endotoxemia. Eur J Pharmacol. 2021;913:174622. 10.1016/j.ejphar.2021.174622. 34748769 10.1016/j.ejphar.2021.174622

[22] Santana-Codina N, Gikandi A, Mancias JD. The role of NCOA4-mediated ferritinophagy in ferroptosis. Adv Exp Med Biol. 2021;1301:41–57. 10.1007/978-3-030-62026-4_4. 34370287 10.1007/978-3-030-62026-4_4

[23] Dutt S, Hamza I, Bartnikas TB. Molecular mechanisms of iron and heme metabolism. Annu Rev Nutr. 2022;42:311–35. 10.1146/annurev-nutr-062320-112625. 35508203 10.1146/annurev-nutr-062320-112625 PMC9398995

[24] Hentze MW, Muckenthaler MU, Andrews NC. Balancing acts: molecular control of mammalian iron metabolism. Cell. 2004;117(3):285–97. 10.1016/s0092-8674(04)00343-5. 15109490 10.1016/s0092-8674(04)00343-5

[25] Park CH, Valore EV, Waring AJ, Ganz T. Hepcidin, a urinary antimicrobial peptide synthesized in the liver. J Biol Chem. 2001;276(11):7806–10. 10.1074/jbc.M008922200. 11113131 10.1074/jbc.M008922200

[26] Arezes J, Foy N, McHugh K, Sawant A, Quinkert D, Terraube V, et al. Erythroferrone inhibits the induction of hepcidin by BMP6. Blood. 2018;132(14):1473–7. 10.1182/blood-2018-06-857995. 30097509 10.1182/blood-2018-06-857995 PMC6238155

[27] Roemhild K, von Maltzahn F, Weiskirchen R, Knüchel R, von Stillfried S, Lammers T. Iron metabolism: pathophysiology and pharmacology. Trends Pharmacol Sci. 2021;42(8):640–56. 10.1016/j.tips.2021.05.001. 34090703 10.1016/j.tips.2021.05.001 PMC7611894

[28] Mastrogiannaki M, Matak P, Mathieu JRR, Delga S, Mayeux P, Vaulont S, et al. Hepatic hypoxia-inducible factor-2 down-regulates hepcidin expression in mice through an erythropoietin-mediated increase in erythropoiesis. Haematologica. 2012;97(6):827–34. 10.3324/haematol.2011.056119. 22207682 10.3324/haematol.2011.056119 PMC3366646

[29] Sullivan JL. Iron and the sex difference in heart disease risk. Lancet. 1981;1(8233):1293–4. 10.1016/s0140-6736(81)92463-6. 6112609 10.1016/s0140-6736(81)92463-6

[30] Silva AMN, Rangel M. The (bio)chemistry of non-transferrin-bound iron. Mol Basel Switz. 2022;27(6):1784. 10.3390/molecules27061784. 10.3390/molecules27061784 PMC8951307 35335148

[31] Porter JB, Walter PB, Neumayr LD, Evans P, Bansal S, Garbowski M, et al. Mechanisms of plasma non-transferrin bound iron generation: insights from comparing transfused diamond blackfan anaemia with sickle cell and thalassaemia patients. Br J Haematol. 2014;167(5):692–6. 10.1111/bjh.13081. 25209728 10.1111/bjh.13081 PMC4577015

[32] Hershko C, Graham G, Bates GW, Rachmilewitz EA. Non-specific serum iron in thalassaemia: an abnormal serum iron fraction of potential toxicity. Br J Haematol. 1978;40(2):255–63. 10.1111/j.1365-2141.1978.tb03662.x. 708645 10.1111/j.1365-2141.1978.tb03662.x

[33] Klip IT, Voors AA, Swinkels DW, et al. Serum ferritin and risk for new-onset heart failure and cardiovascular events in the community. Eur J Heart Fail. 2017;19(3):348–56. 10.1002/ejhf.622. 27758018 10.1002/ejhf.622

[34] Ayala A, Muñoz MF, Argüelles S. Lipid peroxidation: production, metabolism, and signaling mechanisms of malondialdehyde and 4-hydroxy-2-nonenal. Oxid Med Cell Longev. 2014;2014:360438. 10.1155/2014/360438. 24999379 10.1155/2014/360438 PMC4066722

[35] Liang D, Minikes AM, Jiang X. Ferroptosis at the intersection of lipid metabolism and cellular signaling. Mol Cell. 2022;82(12):2215–27. 10.1016/j.molcel.2022.03.022. 35390277 10.1016/j.molcel.2022.03.022 PMC9233073

[36] Sun S, Shen J, Jiang J, Wang F, Min J. Targeting ferroptosis opens new avenues for the development of novel therapeutics. Signal Transduct Target Ther. 2023;8(1):372. 10.1038/s41392-023-01606-1. 37735472 10.1038/s41392-023-01606-1 PMC10514338

[37] Doll S, Proneth B, Tyurina YY, Panzilius E, Kobayashi S, Ingold I, et al. ACSL4 dictates ferroptosis sensitivity by shaping cellular lipid composition. Nat Chem Biol. 2017;13(1):91–8. 10.1038/nchembio.2239. 27842070 10.1038/nchembio.2239 PMC5610546

[38] Hishikawa D, Shindou H, Kobayashi S, Nakanishi H, Taguchi R, Shimizu T. Discovery of a lysophospholipid acyltransferase family essential for membrane asymmetry and diversity. Proc Natl Acad Sci U S A. 2008;105(8):2830–5. 10.1073/pnas.0712245105. 18287005 10.1073/pnas.0712245105 PMC2268545

[39] Li Y, Li M, Feng S, Xu Q, Zhang X, Xiong X, et al. Ferroptosis and endoplasmic reticulum stress in ischemic stroke. Neural Regen Res. 2024;19(3):611–8. 10.4103/1673-5374.380870. 37721292 10.4103/1673-5374.380870 PMC10581588

[40] Sun X, Ou Z, Xie M, Kang R, Fan Y, Niu X, et al. HSPB1 as a novel regulator of ferroptotic cancer cell death. Oncogene. 2015;34(45):5617–25. 10.1038/onc.2015.32. 25728673 10.1038/onc.2015.32 PMC4640181

[41] Gao M, Yi J, Zhu J, Minikes AM, Monian P, Thompson CB, et al. Role of mitochondria in ferroptosis. Mol Cell. 2019;73(2):354-363.e3. 10.1016/j.molcel.2018.10.042. 30581146 10.1016/j.molcel.2018.10.042 PMC6338496

[42] Seiler A, Schneider M, Förster H, Roth S, Wirth EK, Culmsee C, et al. Glutathione peroxidase 4 senses and translates oxidative stress into 12/15-lipoxygenase dependent- and AIF-mediated cell death. Cell Metab. 2008;8(3):237–48. 10.1016/j.cmet.2008.07.005. 18762024 10.1016/j.cmet.2008.07.005

[43] Shintoku R, Takigawa Y, Yamada K, Kubota C, Yoshimoto Y, Takeuchi T, et al. Lipoxygenase-mediated generation of lipid peroxides enhances ferroptosis induced by erastin and RSL3. Cancer Sci. 2017;108(11):2187–94. 10.1111/cas.13380. 28837253 10.1111/cas.13380 PMC5666033

[44] Balderas-Villalobos J, Molina-Muñoz T, Mailloux-Salinas P, Bravo G, Carvajal K, Gómez-Viquez NL. Oxidative stress in cardiomyocytes contributes to decreased SERCA2a activity in rats with metabolic syndrome. Am J Physiol Heart Circ Physiol. 2013;305(9):H1344-1353. 10.1152/ajpheart.00211.2013. 23997093 10.1152/ajpheart.00211.2013

[45] Nikolaienko R, Bovo E, Kahn D, Gracia R, Jamrozik T, Zima AV. Cysteines 1078 and 2991 cross-linking plays a critical role in redox regulation of cardiac ryanodine receptor (RyR). Nat Commun. 2023;14(1):4498. 10.1038/s41467-023-40268-z. 37495581 10.1038/s41467-023-40268-z PMC10372021

[46] McBean GJ. The transsulfuration pathway: a source of cysteine for glutathione in astrocytes. Amino Acids. 2012;42(1):199–205. 10.1007/s00726-011-0864-8. 21369939 10.1007/s00726-011-0864-8

[47] Shimada K, Stockwell BR. tRNA synthase suppression activates de novo cysteine synthesis to compensate for cystine and glutathione deprivation during ferroptosis. Mol Cell Oncol. 2016;3(2):e1091059. 10.1080/23723556.2015.1091059. 27308611 10.1080/23723556.2015.1091059 PMC4905397

[48] Wang L, Liu Y, Du T, et al. ATF3 promotes erastin-induced ferroptosis by suppressing system Xc. Cell Death Differ. 2020;27(2):662–75. 10.1038/s41418-019-0380-z. 31273299 10.1038/s41418-019-0380-z PMC7206049

[49] Zhang Y, Tan H, Daniels JD, et al. Imidazole ketone erastin induces ferroptosis and slows tumor growth in a mouse lymphoma model. Cell Chem Biol. 2019;26(5):623-633.e9. 10.1016/j.chembiol.2019.01.008. 30799221 10.1016/j.chembiol.2019.01.008 PMC6525071

[50] Yan R, Xie E, Li Y, et al. The structure of erastin-bound xCT-4F2hc complex reveals molecular mechanisms underlying erastin-induced ferroptosis. Cell Res. 2022;32(7):687–90. 10.1038/s41422-022-00642-w. 35352032 10.1038/s41422-022-00642-w PMC9253326

[51] Magtanong L, Ko PJ, Dixon SJ. Emerging roles for lipids in non-apoptotic cell death. Cell Death Differ. 2016;23(7):1099–109. 10.1038/cdd.2016.25. 26967968 10.1038/cdd.2016.25 PMC5399169

[52] Sun X, Ou Z, Chen R, Niu X, Chen D, Kang R, et al. Activation of the p62-Keap1-NRF2 pathway protects against ferroptosis in hepatocellular carcinoma cells. Hepatol Baltim Md. 2016;63(1):173–84. 10.1002/hep.28251. 10.1002/hep.28251 PMC4688087 26403645

[53] Hayano M, Yang WS, Corn CK, Pagano NC, Stockwell BR. Loss of cysteinyl-tRNA synthetase (CARS) induces the transsulfuration pathway and inhibits ferroptosis induced by cystine deprivation. Cell Death Differ. 2016;23(2):270–8. 10.1038/cdd.2015.93. 26184909 10.1038/cdd.2015.93 PMC4716307

[54] Friedmann Angeli JP, Schneider M, Proneth B, Tyurina YY, Tyurin VA, Hammond VJ, et al. Inactivation of the ferroptosis regulator Gpx4 triggers acute renal failure in mice. Nat Cell Biol. 2014;16(12):1180–91. 10.1038/ncb3064. 25402683 10.1038/ncb3064 PMC4894846

[55] Yang K, Zeng L, Yuan X, Wang S, Ge A, Xu H, et al. The mechanism of ferroptosis regulating oxidative stress in ischemic stroke and the regulation mechanism of natural pharmacological active components. Biomed Pharmacother. 2022;154:113611. 10.1016/j.biopha.2022.113611. 36081288 10.1016/j.biopha.2022.113611

[56] Agoda N, von Rechenberg M, Zaganjor E, et al. RAS-RAF-MEK-dependent oxidative cell death involving voltage-dependent anion channels. Nature. 2007;447(7146):864–868. 10.1038/nature05859. 10.1038/nature05859 PMC3047570 17568748

[57] Santana-Codina N, Mancias JD. The role of NCOA4-mediated ferritinophagy in health and disease. Pharmaceuticals (Basel). 2018;11(4):114. 10.3390/ph11040114. 30360520 10.3390/ph11040114 PMC6316710

[58] Yang WS, Stockwell BR. Synthetic lethal screening identifies compounds activating iron-dependent, nonapoptotic cell death in oncogenic-RAS-harboring cancer cells. Chem Biol. 2008;15(3):234–45. 10.1016/j.chembiol.2008.02.010. 18355723 10.1016/j.chembiol.2008.02.010 PMC2683762

[59] Dixon SJ, Olzmann JA. The cell biology of ferroptosis. Nat Rev Mol Cell Biol. 2024;25(6):424–42. 10.1038/s41580-024-00703-5. 38366038 10.1038/s41580-024-00703-5 PMC12187608

[60] Doll S, Freitas FP, Shah R, Aldrovandi M, da Silva MC, Ingold I, et al. FSP1 is a glutathione-independent ferroptosis suppressor. Nature. 2019;575(7784):693–8. 10.1038/s41586-019-1707-0. 31634899 10.1038/s41586-019-1707-0

[61] Bersuker K, Hendricks JM, Li Z, Magtanong L, Ford B, Tang PH, et al. The CoQ oxidoreductase FSP1 acts parallel to GPX4 to inhibit ferroptosis. Nature. 2019;575(7784):688–92. 10.1038/s41586-019-1705-2. 31634900 10.1038/s41586-019-1705-2 PMC6883167

[62] Shimada K, Skouta R, Kaplan A, Yang WS, Hayano M, Dixon SJ, et al. Global survey of cell death mechanisms reveals metabolic regulation of ferroptosis. Nat Chem Biol. 2016;12(7):497–503. 10.1038/nchembio.2079. 27159577 10.1038/nchembio.2079 PMC4920070

[63] Miriyala S, Thippakorn C, Chaiswing L, Xu Y, Noel T, Tovmasyan A, et al. Novel role of 4-hydroxy-2-nonenal in AIFm2-mediated mitochondrial stress signaling. Free Radic Biol Med. 2016;91:68–80. 10.1016/j.freeradbiomed.2015.12.002. 26689472 10.1016/j.freeradbiomed.2015.12.002 PMC4761499

[64] Xu J, Wu Y, Song P, Zhang M, Wang S, Zou MH. Proteasome-dependent degradation of guanosine 5’-triphosphate cyclohydrolase I causes tetrahydrobiopterin deficiency in diabetes mellitus. Circulation. 2007;116(8):944–53. 10.1161/CIRCULATIONAHA.106.684795. 17679617 10.1161/CIRCULATIONAHA.106.684795

[65] Hu Q, Wei W, Wu D, Huang F, Li M, Li W, et al. Blockade of GCH1/BH4 axis activates ferritinophagy to mitigate the resistance of colorectal cancer to erastin-induced ferroptosis. Front Cell Dev Biol. 2022;10:810327. 10.3389/fcell.2022.810327. 35223839 10.3389/fcell.2022.810327 PMC8866854

[66] Xue J, Yu C, Sheng W, Zhu W, Luo J, Zhang Q, et al. The Nrf2/GCH1/BH4 axis ameliorates radiation-induced skin injury by modulating the ROS cascade. J Invest Dermatol. 2017;137(10):2059–68. 10.1016/j.jid.2017.05.019. 28596000 10.1016/j.jid.2017.05.019

[67] Kraft VAN, Bezjian CT, Pfeiffer S, Ringelstetter L, Müller C, Zandkarimi F, et al. GTP Cyclohydrolase 1/Tetrahydrobiopterin Counteract Ferroptosis through Lipid Remodeling. ACS Cent Sci. 2020;6(1):41–53. 10.1021/acscentsci.9b01063. 31989025 10.1021/acscentsci.9b01063 PMC6978838

[68] Soula M, Weber RA, Zilka O, Alwaseem H, La K, Yen F, et al. Metabolic determinants of cancer cell sensitivity to canonical ferroptosis inducers. Nat Chem Biol. 2020;16(12):1351–60. 10.1038/s41589-020-0613-y. 32778843 10.1038/s41589-020-0613-y PMC8299533

[69] Vasan K, Werner M, Chandel NS. Mitochondrial metabolism as a target for cancer therapy. Cell Metab. 2020;32(3):341–52. 10.1016/j.cmet.2020.06.019. 32668195 10.1016/j.cmet.2020.06.019 PMC7483781

[70] Madak JT, Bankhead A, Cuthbertson CR, Showalter HD, Neamati N. Revisiting the role of dihydroorotate dehydrogenase as a therapeutic target for cancer. Pharmacol Ther. 2019;195:111–31. 10.1016/j.pharmthera.2018.10.012. 30347213 10.1016/j.pharmthera.2018.10.012

[71] Mao C, Liu X, Zhang Y, Lei G, Yan Y, Lee H, et al. DHODH-mediated ferroptosis defence is a targetable vulnerability in cancer. Nature. 2021;593(7860):586–90. 10.1038/s41586-021-03539-7. 33981038 10.1038/s41586-021-03539-7 PMC8895686

[72] Boukalova S, Hubackova S, Milosevic M, Ezrova Z, Neuzil J, Rohlena J. Dihydroorotate dehydrogenase in oxidative phosphorylation and cancer. Biochim Biophys Acta Mol Basis Dis. 2020;1866(6):165759. 10.1016/j.bbadis.2020.165759. 32151633 10.1016/j.bbadis.2020.165759

[73] Wang F, Min J. DHODH tangoing with GPX4 on the ferroptotic stage. Signal Transduct Target Ther. 2021;6(1):244. 10.1038/s41392-021-00656-7. 34145214 10.1038/s41392-021-00656-7 PMC8212586

[74] Zhang W, Wang J, Liu Z, Zhang L, Jing J, Han L, et al. Iron-dependent ferroptosis participated in benzene-induced anemia of inflammation through IRP1-DHODH-ALOX12 axis. Free Radic Biol Med. 2022;193(Pt 1):122–33. 10.1016/j.freeradbiomed.2022.10.273. 36244588 10.1016/j.freeradbiomed.2022.10.273

[75] Jiang M, Song Y, Liu H, Jin Y, Li R, Zhu X. DHODH inhibition exerts synergistic therapeutic effect with Cisplatin to induce ferroptosis in cervical cancer through regulating mTOR pathway. Cancers. 2023;15(2):546. 10.3390/cancers15020546. 36672495 10.3390/cancers15020546 PMC9856746

[76] Horvath SE, Daum G. Lipids of mitochondria. Prog Lipid Res. 2013;52(4):590–614. 10.1016/j.plipres.2013.07.002. 24007978 10.1016/j.plipres.2013.07.002

[77] Nunnari J, Suomalainen A. Mitochondria: in sickness and in health. Cell. 2012;148(6):1145–59. 10.1016/j.cell.2012.02.035. 22424226 10.1016/j.cell.2012.02.035 PMC5381524

[78] Toyokuni S, Ito F, Yamashita K, Okazaki Y, Akatsuka S. Iron and thiol redox signaling in cancer: an exquisite balance to escape ferroptosis. Free Radic Biol Med. 2017;108:610–26. 10.1016/j.freeradbiomed.2017.04.024. 28433662 10.1016/j.freeradbiomed.2017.04.024

[79] Harigae H, Hino K, Toyokuni S. Iron as soul of life on Earth revisited: from chemical reaction, ferroptosis to therapeutics. Free Radic Biol Med. 2019;133:1–2. 10.1016/j.freeradbiomed.2019.01.042. 30736912 10.1016/j.freeradbiomed.2019.01.042

[80] Missirlis F, Holmberg S, Georgieva T, Dunkov BC, Rouault TA, Law JH. Characterization of mitochondrial ferritin in Drosophila. Proc Natl Acad Sci U S A. 2006;103(15):5893–8. 10.1073/pnas.0601471103. 16571656 10.1073/pnas.0601471103 PMC1458669

[81] Campanella A, Rovelli E, Santambrogio P, Cozzi A, Taroni F, Levi S. Mitochondrial ferritin limits oxidative damage regulating mitochondrial iron availability: hypothesis for a protective role in Friedreich ataxia. Hum Mol Genet. 2009;18(1):1–11. 10.1093/hmg/ddn308. 18815198 10.1093/hmg/ddn308 PMC3298861

[82] Gao G, Zhang N, Wang YQ, Wu Q, Yu P, Shi ZH, et al. Mitochondrial ferritin protects hydrogen peroxide-induced neuronal cell damage. Aging Dis. 2017;8(4):458–70. 10.14336/AD.2016.1108. 28840060 10.14336/AD.2016.1108 PMC5524808

[83] Torii S, Shintoku R, Kubota C, Yaegashi M, Torii R, Sasaki M, et al. An essential role for functional lysosomes in ferroptosis of cancer cells. Biochem J. 2016;473(6):769–77. 10.1042/BJ20150658. 26759376 10.1042/BJ20150658

[84] Kumfu S, Chattipakorn S, Fucharoen S, Chattipakorn N. Mitochondrial calcium uniporter blocker prevents cardiac mitochondrial dysfunction induced by iron overload in thalassemic mice. Biometals Int J Role Met Ions Biol Biochem Med. 2012;25(6):1167–75. 10.1007/s10534-012-9579-x. 10.1007/s10534-012-9579-x 22910858

[85] Khamseekaew J, Kumfu S, Wongjaikam S, Kerdphoo S, Jaiwongkam T, Srichairatanakool S, et al. Effects of iron overload, an iron chelator and a T-type calcium channel blocker on cardiac mitochondrial biogenesis and mitochondrial dynamics in thalassemic mice. Eur J Pharmacol. 2017;799:118–27. 10.1016/j.ejphar.2017.02.015. 28192097 10.1016/j.ejphar.2017.02.015

[86] Colombini M. VDAC structure, selectivity, and dynamics. Biochim Biophys Acta. 2012;1818(6):1457–65. 10.1016/j.bbamem.2011.12.026. 22240010 10.1016/j.bbamem.2011.12.026 PMC3327780

[87] Hodge T, Colombini M. Regulation of metabolite flux through voltage-gating of VDAC channels. J Membr Biol. 1997;157(3):271–9. 10.1007/s002329900235. 9178614 10.1007/s002329900235

[88] Chen M, Cabantchik ZI, Chan S, Chan GCfung, Cheung Y. Iron overload and apoptosis of HL-1 cardiomyocytes: effects of calcium channel blockade. PLoS ONE. 2014;9(11):e112915. 10.1371/journal.pone.0112915. 25390893 10.1371/journal.pone.0112915 PMC4229305

[89] Sumneang N, Siri-Angkul N, Kumfu S, Chattipakorn SC, Chattipakorn N. The effects of iron overload on mitochondrial function, mitochondrial dynamics, and ferroptosis in cardiomyocytes. Arch Biochem Biophys. 2020;680:108241. 10.1016/j.abb.2019.108241. 31891670 10.1016/j.abb.2019.108241

[90] Suzuki S, Venkatesh D, Kanda H, Nakayama A, Hosokawa H, Lee E, et al. GLS2 is a tumor suppressor and a regulator of ferroptosis in hepatocellular carcinoma. Cancer Res. 2022;82(18):3209–22. 10.1158/0008-5472.CAN-21-3914. 35895807 10.1158/0008-5472.CAN-21-3914 PMC11057045

[91] Song X, Liu J, Kuang F, Chen X, Zeh HJ, Kang R, et al. PDK4 dictates metabolic resistance to ferroptosis by suppressing pyruvate oxidation and fatty acid synthesis. Cell Rep. 2021;34(8):108767. 10.1016/j.celrep.2021.108767. 33626342 10.1016/j.celrep.2021.108767

[92] Yuan H, Li X, Zhang X, Kang R, Tang D. Identification of ACSL4 as a biomarker and contributor of ferroptosis. Biochem Biophys Res Commun. 2016;478(3):1338–43. 10.1016/j.bbrc.2016.08.124. 27565726 10.1016/j.bbrc.2016.08.124

[93] Wu J, Minikes AM, Gao M, Bian H, Li Y, Stockwell BR, et al. Intercellular interaction dictates cancer cell ferroptosis via NF2-YAP signalling. Nature. 2019;572(7769):402–6. 10.1038/s41586-019-1426-6. 31341276 10.1038/s41586-019-1426-6 PMC6697195

[94] Stockwell BR. Ferroptosis turns 10: emerging mechanisms, physiological functions, and therapeutic applications. Cell. 2022;185(14):2401–21. 10.1016/j.cell.2022.06.003. 35803244 10.1016/j.cell.2022.06.003 PMC9273022

[95] Chen Y, Li S, Yin M, Li Y, Chen C, Zhang J, et al. Isorhapontigenin Attenuates Cardiac Microvascular Injury in Diabetes via the Inhibition of Mitochondria-Associated Ferroptosis Through PRDX2-MFN2-ACSL4 Pathways. Diabetes. 2023;72(3):389–404. 10.2337/db22-0553. 36367849 10.2337/db22-0553

[96] Feng F, He S, Li X, He J, Luo L. Mitochondria-mediated ferroptosis in diseases therapy: from molecular mechanisms to implications. Aging Dis. 2024;15(2):714–38. 10.14336/AD.2023.0717. 37548939 10.14336/AD.2023.0717 PMC10917537

[97] Peng K, Hu J, Xiao J, Dan G, Yang L, Ye F, et al. Mitochondrial ATP-sensitive potassium channel regulates mitochondrial dynamics to participate in neurodegeneration of Parkinson’s disease. Biochimica et Biophysica Acta (BBA) - Molecular Basis of Disease. 2018;1864(4 Pt A):1086–103. 10.1016/j.bbadis.2018.01.013. 29353068 10.1016/j.bbadis.2018.01.013

[98] Twig G, Shirihai OS. The interplay between mitochondrial dynamics and mitophagy. Antioxid Redox Signal. 2011;14(10):1939–51. 10.1089/ars.2010.3779. 21128700 10.1089/ars.2010.3779 PMC3078508

[99] Jang SK, Ahn SH, Kim G, Kim S, Hong J, Park KS, et al. Inhibition of VDAC1 oligomerization blocks cysteine deprivation-induced ferroptosis via mitochondrial ROS suppression. Cell Death Dis. 2024;15(11):811. 10.1038/s41419-024-07216-1. 39521767 10.1038/s41419-024-07216-1 PMC11550314

[100] Wu H, Wang F, Ta N, Zhang T, Gao W. The multifaceted regulation of mitochondria in ferroptosis. Life Basel Switz. 2021;11(3):222. 10.3390/life11030222. 10.3390/life11030222 PMC8001967 33801920

[101] Neitemeier S, Jelinek A, Laino V, Hoffmann L, Eisenbach I, Eying R, et al. Bid links ferroptosis to mitochondrial cell death pathways. Redox Biol. 2017;12:558–70. 10.1016/j.redox.2017.03.007. 28384611 10.1016/j.redox.2017.03.007 PMC5382034

[102] Jelinek A, Heyder L, Daude M, Plessner M, Krippner S, Grosse R, et al. Mitochondrial rescue prevents glutathione peroxidase-dependent ferroptosis. Free Radic Biol Med. 2018;117:45–57. 10.1016/j.freeradbiomed.2018.01.019. 29378335 10.1016/j.freeradbiomed.2018.01.019

[103] Tang S, Fuß A, Fattahi Z, Culmsee C. Drp1 depletion protects against ferroptotic cell death by preserving mitochondrial integrity and redox homeostasis. Cell Death Dis. 2024;15(8):626. 10.1038/s41419-024-07015-8. 39191736 10.1038/s41419-024-07015-8 PMC11350090

[104] L J, N K, T L, Sj W, T S, H H, et al. Ferroptosis as a p53-mediated activity during tumour suppression. Nature [Internet]. 2015;520(7545). Available from: https://pubmed.ncbi.nlm.nih.gov/25799988/, 10.1038/nature14344. Cited 29 Aug 2025. 10.1038/nature14344 PMC4455927 25799988

[105] Jiang L, Hickman JH, Wang SJ, Gu W. Dynamic roles of p53-mediated metabolic activities in ROS-induced stress responses. Cell Cycle. 2015;14(18):2881–5. 10.1080/15384101.2015.1068479. 26218928 10.1080/15384101.2015.1068479 PMC4825584

[106] Sun F, Zhou JL, Liu ZL, Jiang ZW, Peng H. Dexamethasone induces ferroptosis via P53/SLC7A11/GPX4 pathway in glucocorticoid-induced osteonecrosis of the femoral head. Biochem Biophys Res Commun. 2022;602:149–55. 10.1016/j.bbrc.2022.02.112. 35276555 10.1016/j.bbrc.2022.02.112

[107] Chu B, Kon N, Chen D, Li T, Liu T, Jiang L, et al. ALOX12 is required for p53-mediated tumour suppression through a distinct ferroptosis pathway. Nat Cell Biol. 2019;21(5):579–91. 10.1038/s41556-019-0305-6. 30962574 10.1038/s41556-019-0305-6 PMC6624840

[108] Ou Y, Wang SJ, Li D, Chu B, Gu W. Activation of SAT1 engages polyamine metabolism with p53-mediated ferroptotic responses. Proc Natl Acad Sci U S A. 2016;113(44):E6806–12. 10.1073/pnas.1607152113. 27698118 10.1073/pnas.1607152113 PMC5098629

[109] Song X, Zhu S, Chen P, Hou W, Wen Q, Liu J, et al. AMPK-mediated BECN1 phosphorylation promotes ferroptosis by directly blocking system Xc- activity. Curr Biol. 2018;28(15):2388-2399.e5. 10.1016/j.cub.2018.05.094. 30057310 10.1016/j.cub.2018.05.094 PMC6081251

[110] Zhang Z, Yao Z, Wang L, Ding H, Shao J, Chen A, et al. Activation of ferritinophagy is required for the RNA-binding protein ELAVL1/HuR to regulate ferroptosis in hepatic stellate cells. Autophagy. 2018;14(12):2083–103. 10.1080/15548627.2018.1503146. 30081711 10.1080/15548627.2018.1503146 PMC6984765

[111] Lee H, Zandkarimi F, Zhang Y, Meena JK, Kim J, Zhuang L, et al. Energy-stress-mediated AMPK activation inhibits ferroptosis. Nat Cell Biol. 2020;22(2):225–34. 10.1038/s41556-020-0461-8. 32029897 10.1038/s41556-020-0461-8 PMC7008777

[112] Kerins MJ, Ooi A. The roles of NRF2 in modulating cellular iron homeostasis. Antioxid Redox Signal. 2018;29(17):1756–73. 10.1089/ars.2017.7176. 28793787 10.1089/ars.2017.7176 PMC6208163

[113] Campbell MR, Karaca M, Adamski KN, Chorley BN, Wang X, Bell DA. Novel hematopoietic target genes in the NRF2-mediated transcriptional pathway. Oxid Med Cell Longev. 2013;2013:120305. 10.1155/2013/120305. 23766848 10.1155/2013/120305 PMC3677633

[114] Dodson M, Castro-Portuguez R, Zhang DD. NRF2 plays a critical role in mitigating lipid peroxidation and ferroptosis. Redox Biol. 2019;23:101107. 10.1016/j.redox.2019.101107. 30692038 10.1016/j.redox.2019.101107 PMC6859567

[115] Ishii T, Itoh K, Takahashi S, Sato H, Yanagawa T, Katoh Y, et al. Transcription factor Nrf2 coordinately regulates a group of oxidative stress-inducible genes in macrophages. J Biol Chem. 2000;275(21):16023–9. 10.1074/jbc.275.21.16023. 10821856 10.1074/jbc.275.21.16023

[116] Kwak MK, Itoh K, Yamamoto M, Kensler TW. Enhanced expression of the transcription factor Nrf2 by cancer chemopreventive agents: role of antioxidant response element-like sequences in the nrf2 promoter. Mol Cell Biol. 2002;22(9):2883–92. 10.1128/MCB.22.9.2883-2892.2002. 11940647 10.1128/MCB.22.9.2883-2892.2002 PMC133753

[117] Hayes JD, Dinkova-Kostova AT. The Nrf2 regulatory network provides an interface between redox and intermediary metabolism. Trends Biochem Sci. 2014;39(4):199–218. 10.1016/j.tibs.2014.02.002. 24647116 10.1016/j.tibs.2014.02.002

[118] Salazar M, Rojo AI, Velasco D, de Sagarra RM, Cuadrado A. Glycogen synthase kinase-3beta inhibits the xenobiotic and antioxidant cell response by direct phosphorylation and nuclear exclusion of the transcription factor Nrf2. J Biol Chem. 2006;281(21):14841–51. 10.1074/jbc.M513737200. 16551619 10.1074/jbc.M513737200

[119] Zhong X, Zhang Z, Shen H, Xiong Y, Shah YM, Liu Y, et al. Hepatic NF-κB-Inducing Kinase and Inhibitor of NF-κB Kinase Subunit α Promote Liver Oxidative Stress, Ferroptosis, and Liver Injury. Hepatol Commun. 2021;5(10):1704–20. 10.1002/hep4.1757. 34558831 10.1002/hep4.1757 PMC8485893

[120] Zhu K, Zhu X, Sun S, Yang W, Liu S, Tang Z, et al. Inhibition of TLR4 prevents hippocampal hypoxic-ischemic injury by regulating ferroptosis in neonatal rats. Exp Neurol. 2021;345:113828. 10.1016/j.expneurol.2021.113828. 34343528 10.1016/j.expneurol.2021.113828

[121] Liu Y, Li X, Zhou X, Wang J, Ao X. FADD as a key molecular player in cancer progression. Mol Med. 2022;28(1):132. 10.1186/s10020-022-00560-y. 36348274 10.1186/s10020-022-00560-y PMC9644706

[122] Chang W, Wang M, Zhang Y, Yu F, Hu B, Goljanek-Whysall K, et al. Roles of long noncoding RNAs and small extracellular vesicle-long noncoding RNAs in type 2 diabetes. Traffic. 2022;23(11):526–37. 10.1111/tra.12868. 36109347 10.1111/tra.12868 PMC9828071

[123] Wu Q, Wang H, Liu L, Zhu K, Yu W, Guo J. Hsa_circ_0001546 acts as a miRNA-421 sponge to inhibit the chemoresistance of gastric cancer cells via ATM/Chk2/p53-dependent pathway. Biochem Biophys Res Commun. 2020;521(2):303–9. 10.1016/j.bbrc.2019.10.117. 31668372 10.1016/j.bbrc.2019.10.117

[124] Liu Y, Ao X, Jia Y, Li X, Wang Y, Wang J. The FOXO family of transcription factors: key molecular players in gastric cancer. J Mol Med. 2022;100(7):997–1015. 10.1007/s00109-022-02219-x. 35680690 10.1007/s00109-022-02219-x

[125] Hariharan N, Ghosh S, Palakodeti D. The story of rRNA expansion segments: finding functionality amidst diversity. Wiley Interdiscip Rev RNA. 2023;14(1):e1732. 10.1002/wrna.1732. 35429135 10.1002/wrna.1732

[126] Zhou X, Ao X, Jia Z, Li Y, Kuang S, Du C, et al. Non-coding RNA in cancer drug resistance: underlying mechanisms and clinical applications. Front Oncol. 2022;12:951864. 10.3389/fonc.2022.951864. 36059609 10.3389/fonc.2022.951864 PMC9428469

[127] Zuo YB, Zhang YF, Zhang R, Tian JW, Lv XB, Li R, et al. Ferroptosis in cancer progression: role of noncoding RNAs. Int J Biol Sci. 2022;18(5):1829–43. 10.7150/ijbs.66917. 35342359 10.7150/ijbs.66917 PMC8935228

[128] Zhou Z, Wang Z, Gao J, Lin Z, Wang Y, Shan P, et al. Noncoding RNA-mediated macrophage and cancer cell crosstalk in hepatocellular carcinoma. Mol Ther Oncolytics. 2022;25:98–120. 10.1016/j.omto.2022.03.002. 35506150 10.1016/j.omto.2022.03.002 PMC9024380

[129] Zhou Z, Cao Q, Diao Y, Wang Y, Long L, Wang S, et al. Non-coding RNA-related antitumor mechanisms of marine-derived agents. Front Pharmacol. 2022;13:1053556. 10.3389/fphar.2022.1053556. 36532760 10.3389/fphar.2022.1053556 PMC9752855

[130] Song Y, Wang B, Zhu X, Hu J, Sun J, Xuan J, et al. Human umbilical cord blood-derived MSCs exosome attenuate myocardial injury by inhibiting ferroptosis in acute myocardial infarction mice. Cell Biol Toxicol. 2021;37(1):51–64. 10.1007/s10565-020-09530-8. 32535745 10.1007/s10565-020-09530-8

[131] Xiao FJ, Zhang D, Wu Y, Jia QH, Zhang L, Li YX, et al. miRNA-17-92 protects endothelial cells from erastin-induced ferroptosis through targeting the A20-ACSL4 axis. Biochem Biophys Res Commun. 2019;515(3):448–54. 10.1016/j.bbrc.2019.05.147. 31160087 10.1016/j.bbrc.2019.05.147

[132] Sun W, Shi R, Guo J, Wang H, Shen L, Shi H, et al. miR-135b-3p promotes cardiomyocyte ferroptosis by targeting GPX4 and aggravates myocardial ischemia/reperfusion injury. Front Cardiovasc Med. 2021;8:663832. 10.3389/fcvm.2021.663832. 34485394 10.3389/fcvm.2021.663832 PMC8414249

[133] Fan K, Huang W, Qi H, Song C, He C, Liu Y, et al. The Egr-1/miR-15a-5p/GPX4 axis regulates ferroptosis in acute myocardial infarction. Eur J Pharmacol. 2021;909:174403. 10.1016/j.ejphar.2021.174403. 34339707 10.1016/j.ejphar.2021.174403

[134] He D, Yan L. MiR-29b-3p aggravates cardiac hypoxia/reoxygenation injury via targeting PTX3. Cytotechnology. 2021;73(1):91–100. 10.1007/s10616-020-00446-z. 33505117 10.1007/s10616-020-00446-z PMC7817735

[135] Tang S, Wang Y, Ma T, Lu S, Huang K, Li Q, et al. Mir-30d inhibits cardiomyocytes autophagy promoting ferroptosis after myocardial infarction. Panminerva Med. 2024;66(3):249–55. 10.23736/S0031-0808.20.03979-8. 32720797 10.23736/S0031-0808.20.03979-8

[136] Zhou X, Zhuo M, Zhang Y, Shi E, Ma X, Li H. miR-190a-5p regulates cardiomyocytes response to ferroptosis via directly targeting GLS2. Biochem Biophys Res Commun. 2021;566:9–15. 10.1016/j.bbrc.2021.05.100. 34111670 10.1016/j.bbrc.2021.05.100

[137] Zhang GY, Gao Y, Guo XY, Wang GH, Guo CX. MiR-199a-5p promotes ferroptosis-induced cardiomyocyte death responding to oxygen-glucose deprivation/reperfusion injury via inhibiting Akt/eNOS signaling pathway. Kaohsiung J Med Sci. 2022;38(11):1093–102. 10.1002/kjm2.12605. 36254861 10.1002/kjm2.12605 PMC11896445

[138] Li RL, Fan CH, Gong SY, Kang S. Effect and mechanism of LRP6 on cardiac myocyte ferroptosis in myocardial infarction. Oxid Med Cell Longev. 2021;2021:8963987. 10.1155/2021/8963987. 34712388 10.1155/2021/8963987 PMC8548150

[139] Zheng H, Shi L, Tong C, Liu Y, Hou M. circSnx12 is involved in ferroptosis during heart failure by targeting miR-224-5p. Front Cardiovasc Med. 2021;8:656093. 10.3389/fcvm.2021.656093. 33969020 10.3389/fcvm.2021.656093 PMC8097164

[140] Zhuang S, Ma Y, Zeng Y, Lu C, Yang F, Jiang N, et al. METTL14 promotes doxorubicin-induced cardiomyocyte ferroptosis by regulating the KCNQ1OT1-miR-7-5p-TFRC axis. Cell Biol Toxicol. 2023;39(3):1015–35. 10.1007/s10565-021-09660-7. 34648132 10.1007/s10565-021-09660-7

[141] Wang Q, Yang Y, Fu X, Wang Z, Liu Y, Li M, et al. Long noncoding RNA XXYLT1-AS2 regulates proliferation and adhesion by targeting the RNA binding protein FUS in HUVEC. Atherosclerosis. 2020;298:58–69. 10.1016/j.atherosclerosis.2020.02.018. 32171981 10.1016/j.atherosclerosis.2020.02.018

[142] Sun L, Zhu W, Zhao P, Wang Q, Fan B, Zhu Y, et al. Long noncoding RNA UCA1 from hypoxia-conditioned hMSC-derived exosomes: a novel molecular target for cardioprotection through miR-873-5p/XIAP axis. Cell Death Dis. 2020;11(8):696. 10.1038/s41419-020-02783-5. 32826854 10.1038/s41419-020-02783-5 PMC7442657

[143] Wu G, Cai J, Han Y, Chen J, Huang ZP, Chen C, et al. LincRNA-p21 regulates neointima formation, vascular smooth muscle cell proliferation, apoptosis, and atherosclerosis by enhancing p53 activity. Circulation. 2014;130(17):1452–65. 10.1161/CIRCULATIONAHA.114.011675. 25156994 10.1161/CIRCULATIONAHA.114.011675 PMC4244705

[144] Liu Y, Ao X, Zhou X, Du C, Kuang S. The regulation of PBXs and their emerging role in cancer. J Cell Mol Med. 2022;26(5):1363–79. 10.1111/jcmm.17196. 35068042 10.1111/jcmm.17196 PMC8899182

[145] Ao X, Ding W, Li X, Xu Q, Chen X, Zhou X, et al. Non-coding RNAs regulating mitochondrial function in cardiovascular diseases. J Mol Med Berl Ger. 2023;101(5):501–26. 10.1007/s00109-023-02305-8. 10.1007/s00109-023-02305-8 37014377

[146] Liu L, Yao H, Zhou X, Chen J, Chen G, Shi X, et al. MiR-15a-3p regulates ferroptosis via targeting glutathione peroxidase GPX4 in colorectal cancer. Mol Carcinog. 2022;61(3):301–10. 10.1002/mc.23367. 34727409 10.1002/mc.23367

[147] Cloer EW, Goldfarb D, Schrank TP, Weissman BE, Major MB. NRF2 activation in cancer: from DNA to protein. Cancer Res. 2019;79(5):889–98. 10.1158/0008-5472.CAN-18-2723. 30760522 10.1158/0008-5472.CAN-18-2723 PMC6397706

[148] Zhang J, Sun H, Zhu L, Du L, Ma Y, Ma Y, et al. Micro Ribonucleic Acid 27a Aggravates Ferroptosis During Early Ischemic Stroke of Rats Through Nuclear Factor Erythroid-2-Related Factor 2. Neuroscience. 2022;504:10–20. 10.1016/j.neuroscience.2022.09.014. 36180007 10.1016/j.neuroscience.2022.09.014

[149] Liu Y, Wang Y, Li X, Jia Y, Wang J, Ao X. FOXO3a in cancer drug resistance. Cancer Lett. 2022;540:215724. 10.1016/j.canlet.2022.215724. 35545128 10.1016/j.canlet.2022.215724

[150] Sun Z, Wu J, Bi Q, Wang W. Exosomal lncRNA TUG1 derived from human urine-derived stem cells attenuates renal ischemia/reperfusion injury by interacting with SRSF1 to regulate ASCL4-mediated ferroptosis. Stem Cell Res Ther. 2022;13(1):297. 10.1186/s13287-022-02986-x. 35841017 10.1186/s13287-022-02986-x PMC9284726

[151] Yang H, Hu Y, Weng M, Liu X, Wan P, Hu Y, et al. Hypoxia inducible lncRNA-CBSLR modulates ferroptosis through m6A-YTHDF2-dependent modulation of CBS in gastric cancer. J Adv Res. 2022;37:91–106. 10.1016/j.jare.2021.10.001. 35499052 10.1016/j.jare.2021.10.001 PMC9039740

[152] Zhang R, Pan T, Xiang Y, Zhang M, Xie H, Liang Z, et al. Curcumenol triggered ferroptosis in lung cancer cells via lncRNA H19/miR-19b-3p/FTH1 axis. Bioact Mater. 2022;13:23–36. 10.1016/j.bioactmat.2021.11.013. 35224289 10.1016/j.bioactmat.2021.11.013 PMC8843976

[153] Thomson DW, Dinger ME. Endogenous microRNA sponges: evidence and controversy. Nat Rev Genet. 2016;17(5):272–83. 10.1038/nrg.2016.20. 27040487 10.1038/nrg.2016.20

[154] Fan X, Li A, Yan Z, Geng X, Lian L, Lv H, et al. From iron metabolism to ferroptosis: pathologic changes in coronary heart disease. Oxid Med Cell Longev. 2022;2022:6291889. 10.1155/2022/6291889. 35993022 10.1155/2022/6291889 PMC9385341

[155] Zhang JK, Zhang Z, Guo ZA, Fu Y, Chen XJ, Chen WJ, et al. The BMSC-derived exosomal lncRNA Mir9-3hg suppresses cardiomyocyte ferroptosis in ischemia-reperfusion mice via the Pum2/PRDX6 axis. Nutr Metab Cardiovasc Dis. 2022;32(2):515–27. 10.1016/j.numecd.2021.10.017. 34953631 10.1016/j.numecd.2021.10.017

[156] Zhang Y, Jia DD, Zhang YF, Cheng MD, Zhu WX, Li PF. The emerging function and clinical significance of circRNAs in thyroid cancer and autoimmune thyroid diseases. Int J Biol Sci. 2021;17(7):1731–41. 10.7150/ijbs.55381. 33994857 10.7150/ijbs.55381 PMC8120456

[157] Peng JJ, Song WT, Yao F, Zhang X, Peng J, Luo XJ, et al. Involvement of regulated necrosis in blinding diseases: focus on necroptosis and ferroptosis. Exp Eye Res. 2020;191:107922. 10.1016/j.exer.2020.107922. 31923413 10.1016/j.exer.2020.107922

[158] Kim EH, Wong SW, Martinez J. Programmed necrosis and disease: we interrupt your regular programming to bring you necroinflammation. Cell Death Differ. 2019;26(1):25–40. 10.1038/s41418-018-0179-3. 30349078 10.1038/s41418-018-0179-3 PMC6294794

[159] Proneth B, Conrad M. Ferroptosis and necroinflammation, a yet poorly explored link. Cell Death Differ. 2019;26(1):14–24. 10.1038/s41418-018-0173-9. 30082768 10.1038/s41418-018-0173-9 PMC6294786

[160] Wang T, Fu X, Chen Q, Patra JK, Wang D, Wang Z, et al. Arachidonic Acid Metabolism and Kidney Inflammation. Int J Mol Sci. 2019;20(15):3683. 10.3390/ijms20153683. 31357612 10.3390/ijms20153683 PMC6695795

[161] Yang WS, SriRamaratnam R, Welsch ME, Shimada K, Skouta R, Viswanathan VS, et al. Regulation of ferroptotic cancer cell death by GPX4. Cell. 2014;156(1–2):317–31. 10.1016/j.cell.2013.12.010. 24439385 10.1016/j.cell.2013.12.010 PMC4076414

[162] Araújo AC, Wheelock CE, Haeggström JZ. The eicosanoids, redox-regulated lipid mediators in immunometabolic disorders. Antioxid Redox Signal. 2018;29(3):275–96. 10.1089/ars.2017.7332. 28978222 10.1089/ars.2017.7332

[163] Loscalzo J. Membrane redox state and apoptosis: death by peroxide. Cell Metab. 2008;8(3):182–3. 10.1016/j.cmet.2008.08.004. 18762018 10.1016/j.cmet.2008.08.004

[164] Dar HH, Tyurina YY, Mikulska-Ruminska K, Shrivastava I, Ting HC, Tyurin VA, et al. *Pseudomonas aeruginosa* utilizes host polyunsaturated phosphatidylethanolamines to trigger theft-ferroptosis in bronchial epithelium. J Clin Invest. 2018;128(10):4639–53. 10.1172/JCI99490. 30198910 10.1172/JCI99490 PMC6159971

[165] Kim S, Keku TO, Martin C, Galanko J, Woosley JT, Schroeder JC, et al. Circulating levels of inflammatory cytokines and risk of colorectal adenomas. Cancer Res. 2008;68(1):323–8. 10.1158/0008-5472.CAN-07-2924. 18172326 10.1158/0008-5472.CAN-07-2924 PMC2675825

[166] Wen Q, Liu J, Kang R, Zhou B, Tang D. The release and activity of HMGB1 in ferroptosis. Biochem Biophys Res Commun. 2019;510(2):278–83. 10.1016/j.bbrc.2019.01.090. 30686534 10.1016/j.bbrc.2019.01.090

[167] Li W, Feng G, Gauthier JM, Lokshina I, Higashikubo R, Evans S, et al. Ferroptotic cell death and TLR4/Trif signaling initiate neutrophil recruitment after heart transplantation. J Clin Invest. 2019;129(6):2293–304. 10.1172/JCI126428. 30830879 10.1172/JCI126428 PMC6546457

[168] Wu MY, Yiang GT, Liao WT, Tsai APY, Cheng YL, Cheng PW, et al. Current Mechanistic Concepts in Ischemia and Reperfusion Injury. Cell Physiol Biochem Int J Exp Cell Physiol Biochem Pharmacol. 2018;46(4):1650–67. 10.1159/000489241. 10.1159/000489241 29694958

[169] Zhang Z, Wu Y, Yuan S, Zhang P, Zhang J, Li H, et al. Glutathione peroxidase 4 participates in secondary brain injury through mediating ferroptosis in a rat model of intracerebral hemorrhage. Brain Res. 2018;1701:112–25. 10.1016/j.brainres.2018.09.012. 30205109 10.1016/j.brainres.2018.09.012

[170] Yang Z, Shi J, Chen L, Fu C, Shi D, Qu H. Role of pyroptosis and ferroptosis in the progression of atherosclerotic plaques. Front Cell Dev Biol. 2022;10:811196. 10.3389/fcell.2022.811196. 35186925 10.3389/fcell.2022.811196 PMC8850398

[171] Glass CK, Witztum JL. Atherosclerosis. The road ahead. Cell. 2001;104(4):503–16. 10.1016/s0092-8674(01)00238-0. 11239408 10.1016/s0092-8674(01)00238-0

[172] Lu J, Guo S, Xue X, et al. Identification of a novel series of anti-inflammatory and anti-oxidative phospholipid oxidation products containing the cyclopentenone moiety in vitro and in vivo: implication in atherosclerosis. J Biol Chem. 2017;292(13):5378–91. 10.1074/jbc.M116.751909. 28202546 10.1074/jbc.M116.751909 PMC5392682

[173] Berliner JA, Leitinger N, Tsimikas S. The role of oxidized phospholipids in atherosclerosis. J Lipid Res. 2009;50(Suppl):S207–12. 10.1194/jlr.R800074-JLR200. 19059906 10.1194/jlr.R800074-JLR200 PMC2674746

[174] Lee S, Birukov KG, Romanoski CE, Springstead JR, Lusis AJ, Berliner JA. Role of phospholipid oxidation products in atherosclerosis. Circ Res. 2012;111(6):778–99. 10.1161/CIRCRESAHA.111.256859. 22935534 10.1161/CIRCRESAHA.111.256859 PMC3563283

[175] Berliner JA, Watson AD. A role for oxidized phospholipids in atherosclerosis [published correction appears in N Engl J Med. 2005 Oct 27;353(17):1869]. N Engl J Med. 2005;353(1):9–11. 10.1056/NEJMp058118. 16000351 10.1056/NEJMp058118

[176] Kraml P. The role of iron in the pathogenesis of atherosclerosis. Physiol Res. 2017:S55–67. 10.33549/physiolres.933589 10.33549/physiolres.933589 28379030

[177] Stadler N, Lindner RA, Davies MJ. Direct detection and quantification of transition metal ions in human atherosclerotic plaques: evidence for the presence of elevated levels of iron and copper. Arterioscler Thromb Vasc Biol. 2004;24(5):949–54. 10.1161/01.ATV.0000124892.90999.cb. 15001454 10.1161/01.ATV.0000124892.90999.cb

[178] Gustafsson H, Hallbeck M, Norell M, Lindgren M, Engström M, Rosén A, et al. Fe(III) distribution varies substantially within and between atherosclerotic plaques. Magn Reson Med. 2014;71(2):885–92. 10.1002/mrm.24687. 23447110 10.1002/mrm.24687

[179] Vinchi F, Porto G, Simmelbauer A, Altamura S, Passos ST, Garbowski M, et al. Atherosclerosis is aggravated by iron overload and ameliorated by dietary and pharmacological iron restriction. Eur Heart J. 2020;41(28):2681–95. 10.1093/eurheartj/ehz112. 30903157 10.1093/eurheartj/ehz112

[180] Celletti FL, Waugh JM, Amabile PG, Brendolan A, Hilfiker PR, Dake MD. Vascular endothelial growth factor enhances atherosclerotic plaque progression. Nat Med. 2001;7(4):425–9. 10.1038/86490. 11283668 10.1038/86490

[181] Holm PW, Slart RHJA, Zeebregts CJ, Hillebrands JL, Tio RA. Atherosclerotic plaque development and instability: a dual role for VEGF. Ann Med. 2009;41(4):257–64. 10.1080/07853890802516507. 19089693 10.1080/07853890802516507

[182] Zager RA. Parenteral iron treatment induces MCP-1 accumulation in plasma, normal kidneys, and in experimental nephropathy. Kidney Int. 2005;68(4):1533–42. 10.1111/j.1523-1755.2005.00565.x. 16164630 10.1111/j.1523-1755.2005.00565.x

[183] Bai T, Li M, Liu Y, Qiao Z, Wang Z. Inhibition of ferroptosis alleviates atherosclerosis through attenuating lipid peroxidation and endothelial dysfunction in mouse aortic endothelial cell. Free Radic Biol Med. 2020;160:92–102. 10.1016/j.freeradbiomed.2020.07.026. 32768568 10.1016/j.freeradbiomed.2020.07.026

[184] Duffy SJ, Biegelsen ES, Holbrook M, Russell JD, Gokce N, Keaney JF, et al. Iron chelation improves endothelial function in patients with coronary artery disease. Circulation. 2001;103(23):2799–804. 10.1161/01.cir.103.23.2799. 11401935 10.1161/01.cir.103.23.2799

[185] Zhou Y, Zhou H, Hua L, et al. Verification of ferroptosis and pyroptosis and identification of PTGS2 as the hub gene in human coronary artery atherosclerosis. Free Radic Biol Med. 2021;171:55–68. 10.1016/j.freeradbiomed.2021.05.009. 33974977 10.1016/j.freeradbiomed.2021.05.009

[186] Bai T, Li M, Liu Y, Qiao Z, Wang Z. Inhibition of ferroptosis alleviates atherosclerosis through attenuating lipid peroxidation and endothelial dysfunction in mouse aortic endothelial cell. Free Radic Biol Med. 2020;160:92–102. 10.1016/j.freeradbiomed.2020.07.026. 32768568 10.1016/j.freeradbiomed.2020.07.026

[187] Liu R, Dai L, Jia S, Geng S, Niu Y, Chen J, et al. Fut8 regulated Unc5b hyperfucosylation reduces macrophage emigration and accelerates atherosclerosis development via the ferroptosis pathway. Free Radic Biol Med. 2025;235:1–14. 10.1016/j.freeradbiomed.2025.04.025. 40262667 10.1016/j.freeradbiomed.2025.04.025

[188] Yang Y, Chen Z, Song D, Wu J, Wang J, YouyouYan null. Inhibition of ferroptosis alleviates atherosclerosis and foam cell formation by regulating lipid metabolism via AMPK activation. Int Immunopharmacol. 2025;153:114553. 10.1016/j.intimp.2025.114553 10.1016/j.intimp.2025.114553 40147262

[189] Zhu H, Sun A. Programmed necrosis in heart disease: molecular mechanisms and clinical implications. J Mol Cell Cardiol. 2018;116:125–34. 10.1016/j.yjmcc.2018.01.018. 29426003 10.1016/j.yjmcc.2018.01.018

[190] De SD, Krishna S, Jethwa A. Iron status and its association with coronary heart disease: systematic review and meta-analysis of prospective studies. Atherosclerosis. 2015;238(2):296–303. 10.1016/j.atherosclerosis.2014.12.018. 25544180 10.1016/j.atherosclerosis.2014.12.018

[191] Park TJ, Park JH, Lee GS, Lee JY, Shin JH, Kim MW, et al. Quantitative proteomic analyses reveal that GPX4 downregulation during myocardial infarction contributes to ferroptosis in cardiomyocytes. Cell Death Dis. 2019;10(11):835. 10.1038/s41419-019-2061-8. 31685805 10.1038/s41419-019-2061-8 PMC6828761

[192] Baba Y, Higa JK, Shimada BK, Horiuchi KM, Suhara T, Kobayashi M, et al. Protective effects of the mechanistic target of rapamycin against excess iron and ferroptosis in cardiomyocytes. Am J Physiol Heart Circ Physiol. 2018;314(3):H659–68. 10.1152/ajpheart.00452.2017. 29127238 10.1152/ajpheart.00452.2017 PMC5899260

[193] Gao F, Zhao Y, Zhang B, et al. Suppression of lncRNA Gm47283 attenuates myocardial infarction via miR-706/ Ptgs2/ferroptosis axis. Bioengineered. 2022;13(4):10786–802. 10.1080/21655979.2022.2065743. 35485136 10.1080/21655979.2022.2065743 PMC9208485

[194] Qin Y, Qiao Y, Wang D, Tang C, Yan G. Ferritinophagy and ferroptosis in cardiovascular disease: mechanisms and potential applications. Biomed Pharmacother Biomedecine Pharmacother. 2021;141:111872. 10.1016/j.biopha.2021.111872. 10.1016/j.biopha.2021.111872 34246187

[195] Heusch G. Myocardial ischaemia-reperfusion injury and cardioprotection in perspective. Nat Rev Cardiol. 2020;17(12):773–89. 10.1038/s41569-020-0403-y. 32620851 10.1038/s41569-020-0403-y

[196] Yellon DM, Hausenloy DJ. Myocardial reperfusion injury. N Engl J Med. 2007;357(11):1121–35. 10.1056/NEJMra071667. 17855673 10.1056/NEJMra071667

[197] Hausenloy DJ, Yellon DM. Myocardial ischemia-reperfusion injury: a neglected therapeutic target. J Clin Invest. 2013;123(1):92–100. 10.1172/JCI62874. 23281415 10.1172/JCI62874 PMC3533275

[198] Zhang T, Zhang Y, Cui M, Jin L, Wang Y, Lv F, et al. CaMKII is a RIP3 substrate mediating ischemia- and oxidative stress–induced myocardial necroptosis. Nat Med. 2016;22(2):175–82. 10.1038/nm.4017. 26726877 10.1038/nm.4017

[199] Bulluck H, Rosmini S, Abdel-Gadir A, et al. Residual myocardial iron following intramyocardial hemorrhage during the convalescent phase of reperfused ST-segment-elevation myocardial infarction and adverse left ventricular remodeling. Circ Cardiovasc Imaging. 2016;9(10):e004940. 10.1161/CIRCIMAGING.116.004940. 27894068 10.1161/CIRCIMAGING.116.004940 PMC5068185

[200] Baba Y, Higa JK, Shimada BK, et al. Protective effects of the mechanistic target of rapamycin against excess iron and ferroptosis in cardiomyocytes. Am J Physiol Heart Circ Physiol. 2018;314(3):H659–68. 10.1152/ajpheart.00452.2017. 29127238 10.1152/ajpheart.00452.2017 PMC5899260

[201] Farmer EE, Mueller MJ. ROS-mediated lipid peroxidation and RES-activated signaling. Annu Rev Plant Biol. 2013;64:429–50. 10.1146/annurev-arplant-050312-120132. 23451784 10.1146/annurev-arplant-050312-120132

[202] Scindia Y, Leeds J, Swaminathan S. Iron homeostasis in healthy kidney and its role in acute kidney injury. Semin Nephrol. 2019;39(1):76–84. 10.1016/j.semnephrol.2018.10.006. 30606409 10.1016/j.semnephrol.2018.10.006

[203] Fang X, Wang H, Han D, Xie E, Yang X, Wei J, et al. Ferroptosis as a target for protection against cardiomyopathy. Proc Natl Acad Sci U S A. 2019;116(7):2672–80. 10.1073/pnas.1821022116. 30692261 10.1073/pnas.1821022116 PMC6377499

[204] Stamenkovic A, O’Hara KA, Nelson DC, et al. Oxidized phosphatidylcholines trigger ferroptosis in cardiomyocytes during ischemia-reperfusion injury. Am J Physiol Heart Circ Physiol. 2021;320(3):H1170–84. 10.1152/ajpheart.00237.2020. 33513080 10.1152/ajpheart.00237.2020

[205] Gao M, Monian P, Quadri N, Ramasamy R, Jiang X. Glutaminolysis and transferrin regulate ferroptosis. Mol Cell. 2015;59(2):298–308. 10.1016/j.molcel.2015.06.011. 26166707 10.1016/j.molcel.2015.06.011 PMC4506736

[206] Tang LJ, Luo XJ, Tu H, et al. Ferroptosis occurs in phase of reperfusion but not ischemia in rat heart following ischemia or ischemia/reperfusion. Naunyn Schmiedebergs Arch Pharmacol. 2021;394(2):401–10. 10.1007/s00210-020-01932-z. 32621060 10.1007/s00210-020-01932-z

[207] Ml H, Nh M. Molecular oxygen: friend and foe. The role of the oxygen free radical system in the calcium paradox, the oxygen paradox and ischemia/reperfusion injury. J Mol Cell Cardiol [Internet]. 1984;16(11). Available from: https://pubmed.ncbi.nlm.nih.gov/6394765/, 10.1016/s0022-2828(84)80011-5. Cited 29 Aug 2025. 10.1016/s0022-2828(84)80011-5 6394765

[208] Lj T, Xj L, H T, H C, Xm X, Ns L, et al. Ferroptosis occurs in phase of reperfusion but not ischemia in rat heart following ischemia or ischemia/reperfusion. Naunyn Schmiedebergs Arch Pharmacol [Internet]. 2021;394(2). Available from: https://pubmed.ncbi.nlm.nih.gov/32621060/, 10.1007/s00210-020-01932-z. Cited 29 Aug 2025. 10.1007/s00210-020-01932-z 32621060

[209] Ghosh AP, Klocke BJ, Ballestas ME, Roth KA. CHOP potentially co-operates with FOXO3a in neuronal cells to regulate PUMA and BIM expression in response to ER stress. PLoS ONE. 2012;7(6):e39586. 10.1371/journal.pone.0039586. 22761832 10.1371/journal.pone.0039586 PMC3386252

[210] Lee YS, Lee DH, Choudry HA, Bartlett DL, Lee YJ. Ferroptosis-Induced endoplasmic reticulum stress: cross-talk between ferroptosis and apoptosis. Mol Cancer Res. 2018;16(7):1073–6. 10.1158/1541-7786.MCR-18-0055. 29592897 10.1158/1541-7786.MCR-18-0055 PMC6030493

[211] Dixon SJ, Patel DN, Welsch M, Skouta R, Lee ED, Hayano M, et al. Pharmacological inhibition of cystine-glutamate exchange induces endoplasmic reticulum stress and ferroptosis. Elife. 2014;3:e02523. 10.7554/eLife.02523. 24844246 10.7554/eLife.02523 PMC4054777

[212] Cao SS, Kaufman RJ. Endoplasmic reticulum stress and oxidative stress in cell fate decision and human disease. Antioxid Redox Signal. 2014;21(3):396–413. 10.1089/ars.2014.5851. 24702237 10.1089/ars.2014.5851 PMC4076992

[213] Kremastinos DT, Farmakis D. Iron overload cardiomyopathy in clinical practice. Circulation. 2011;124(20):2253–63. 10.1161/CIRCULATIONAHA.111.050773. 22083147 10.1161/CIRCULATIONAHA.111.050773

[214] X F, Z C, H W, D H, Q C, P Z, et al. Loss of Cardiac Ferritin H Facilitates Cardiomyopathy via Slc7a11-Mediated Ferroptosis. Circ Res [Internet]. 2020;127(4). Available from: https://pubmed.ncbi.nlm.nih.gov/32349646/, 10.1161/CIRCRESAHA.120.316509. Cited 29 Aug 2025. 10.1161/CIRCRESAHA.120.316509 32349646

[215] Chen Q, Thompson J, Hu Y, Das A, Lesnefsky EJ. Cardiac specific knockout of p53 decreases ER stress-induced mitochondrial damage. Front Cardiovasc Med. 2019;6:10. 10.3389/fcvm.2019.00010. 30838215 10.3389/fcvm.2019.00010 PMC6389610

[216] Narezkina A, Narayan HK, Zemljic-Harpf AE. Molecular mechanisms of anthracycline cardiovascular toxicity. Clin Sci. 2021;135(10):1311–32. 10.1042/CS20200301. 10.1042/CS20200301 PMC10866014 34047339

[217] S N, Y M, Q F, Y SW, N T, K I, et al. Endoplasmic Reticulum Selective Autophagy Alleviates Anthracycline-Induced Cardiotoxicity. JACC CardioOncology [Internet]. 2023;5(5). Available from: https://pubmed.ncbi.nlm.nih.gov/37969644/, 10.1016/j.jaccao.2023.05.009. Cited 29 Aug 2025. 10.1016/j.jaccao.2023.05.009 PMC10635891 37969644

[218] Rupérez AI, Olza J, Gil-Campos M, et al. Association of genetic polymorphisms for glutathione peroxidase genes with obesity in Spanish children. J Nutrigenet Nutrigenomics. 2014;7(3):130–42. 10.1159/000368833. 25471206 10.1159/000368833

[219] Katunga LA, Gudimella P, Efird JT, et al. Obesity in a model of gpx4 haploinsufficiency uncovers a causal role for lipid-derived aldehydes in human metabolic disease and cardiomyopathy [published correction appears in Mol Metab. 2015 Jun 19;4(10):753. 10.1016/j.molmet.2015.06.005]. Mol Metab. 2015;4(6):493–506. Published 21 Apr 2015. 10.1016/j.molmet.2015.04.001. PMC4443294 26042203

[220] Ma L, Li XP, Ji HS, Liu YF, Li EZ. Baicalein protects rats with diabetic cardiomyopathy against oxidative stress and inflammation injury via phosphatidylinositol 3-kinase (PI3K)/AKT pathway. Med Sci Monit. 2018;24:5368–75. 10.12659/MSM.911455. 30070262 10.12659/MSM.911455 PMC6085984

[221] Baseler WA, Dabkowski ER, Jagannathan R, et al. Reversal of mitochondrial proteomic loss in Type 1 diabetic heart with overexpression of phospholipid hydroperoxide glutathione peroxidase. Am J Physiol Regul Integr Comp Physiol. 2013;304(7):R553–65. 10.1152/ajpregu.00249.2012. 23408027 10.1152/ajpregu.00249.2012 PMC3627941

[222] Huynh K, Kiriazis H, Du XJ, et al. Coenzyme Q10 attenuates diastolic dysfunction, cardiomyocyte hypertrophy and cardiac fibrosis in the db/db mouse model of type 2 diabetes. Diabetologia. 2012;55(5):1544–53. 10.1007/s00125-012-2495-3. 22374176 10.1007/s00125-012-2495-3

[223] Chen A, Chen Z, Xia Y, et al. Liraglutide attenuates NLRP3 inflammasome-dependent pyroptosis via regulating SIRT1/NOX4/ROS pathway in H9c2 cells. Biochem Biophys Res Commun. 2018;499(2):267–72. 10.1016/j.bbrc.2018.03.142. 29571736 10.1016/j.bbrc.2018.03.142

[224] Kostoryz EL, Yourtee DM. Oxidative mutagenesis of doxorubicin-Fe(III) complex. Mutat Res. 2001;490(2):131–9. 10.1016/s1383-5718(00)00158-3. 11342239 10.1016/s1383-5718(00)00158-3

[225] Li H, Lin L, Xia YL, Xie Y, Yang X. Research progress on the role of ferroptosis in cardiovascular disease. Front Cardiovasc Med. 2022;9:1077332. 10.3389/fcvm.2022.1077332. 36620630 10.3389/fcvm.2022.1077332 PMC9815775

[226] X F, H W, D H, E X, X Y, J W, et al. Ferroptosis as a target for protection against cardiomyopathy. Proc Natl Acad Sci U S A [Internet]. 2019;116(7). Available from: https://pubmed.ncbi.nlm.nih.gov/30692261/, 10.1073/pnas.1821022116. Cited 29 Aug 2025. 10.1073/pnas.1821022116 PMC6377499 30692261

[227] Jankowska EA, Kasztura M, Sokolski M, Bronisz M, Nawrocka S, Oleśkowska-Florek W, et al. Iron deficiency defined as depleted iron stores accompanied by unmet cellular iron requirements identifies patients at the highest risk of death after an episode of acute heart failure. Eur Heart J. 2014;35(36):2468–76. 10.1093/eurheartj/ehu235. 24927731 10.1093/eurheartj/ehu235

[228] Liu B, Zhao C, Li H, Chen X, Ding Y, Xu S. Puerarin protects against heart failure induced by pressure overload through mitigation of ferroptosis. Biochem Biophys Res Commun. 2018;497(1):233–40. 10.1016/j.bbrc.2018.02.061. 29427658 10.1016/j.bbrc.2018.02.061

[229] Omiya S, Hikoso S, Imanishi Y, et al. Downregulation of ferritin heavy chain increases labile iron pool, oxidative stress and cell death in cardiomyocytes. J Mol Cell Cardiol. 2009;46(1):59–66. 10.1016/j.yjmcc.2008.09.714. 18992754 10.1016/j.yjmcc.2008.09.714

[230] Fujisue K, Sugamura K, Kurokawa H, et al. Colchicine improves survival, left ventricular remodeling, and chronic cardiac function after acute myocardial infarction. Circ J. 2017;81(8):1174–82. 10.1253/circj.CJ-16-0949. 28420825 10.1253/circj.CJ-16-0949

[231] Liu B, Zhao C, Li H, Chen X, Ding Y, Xu S. Puerarin protects against heart failure induced by pressure overload through mitigation of ferroptosis. Biochem Biophys Res Commun. 2018;497(1):233–40. 10.1016/j.bbrc.2018.02.061. 29427658 10.1016/j.bbrc.2018.02.061

[232] Tang M, Huang Z, Luo X, et al. Ferritinophagy activation and sideroflexin1-dependent mitochondria iron overload is involved in apelin-13-induced cardiomyocytes hypertrophy. Free Radic Biol Med. 2019;134:445–57. 10.1016/j.freeradbiomed.2019.01.052. 30731113 10.1016/j.freeradbiomed.2019.01.052

[233] Yang WS, Stockwell BR. Synthetic lethal screening identifies compounds activating iron-dependent, nonapoptotic cell death in oncogenic-RAS-harboring cancer cells. Chem Biol. 2008;15(3):234–45. 10.1016/j.chembiol.2008.02.010. 18355723 10.1016/j.chembiol.2008.02.010 PMC2683762

[234] Gao M, Monian P, Jiang X. Metabolism and iron signaling in ferroptotic cell death. Oncotarget. 2015;6(34):35145–6. 10.18632/oncotarget.5671. 26387139 10.18632/oncotarget.5671 PMC4742090

[235] Chen X, Xu S, Zhao C, Liu B. Role of TLR4/NADPH oxidase 4 pathway in promoting cell death through autophagy and ferroptosis during heart failure. Biochem Biophys Res Commun. 2019;516(1):37–43. 10.1016/j.bbrc.2019.06.015. 31196626 10.1016/j.bbrc.2019.06.015

[236] Brown IAM, Diederich L, Good ME, DeLalio LJ, Murphy SA, Cortese-Krott MM, et al. Vascular smooth muscle remodeling in conductive and resistance arteries in hypertension. Arterioscler Thromb Vasc Biol. 2018;38(9):1969–85. 10.1161/ATVBAHA.118.311229. 30354262 10.1161/ATVBAHA.118.311229 PMC6205219

[237] Zhang Z, Tang J, Song J, Xie M, Liu Y, Dong Z, et al. Elabela alleviates ferroptosis, myocardial remodeling, fibrosis and heart dysfunction in hypertensive mice by modulating the IL-6/STAT3/GPX4 signaling. Free Radic Biol Med. 2022;181:130–42. 10.1016/j.freeradbiomed.2022.01.020. 35122997 10.1016/j.freeradbiomed.2022.01.020

[238] Zhang S, Bei Y, Huang Y, et al. Induction of ferroptosis promotes vascular smooth muscle cell phenotypic switching and aggravates neointimal hyperplasia in mice. Mol Med. 2022;28(1):121. 10.1186/s10020-022-00549-7. 36192693 10.1186/s10020-022-00549-7 PMC9528136

[239] Lee DH, Kang SK, Choi WJ, et al. Association between serum ferritin and hypertension according to the working type in Korean men: the fifth Korean National Health and nutrition examination survey 2010-2012. Ann Occup Environ Med. 2018;30(1):40. 10.1186/s40557-018-0251-y. 29942520 10.1186/s40557-018-0251-y PMC5996563

[240] Määttä KM, Nikkari ST, Kunnas TA. Genetic variant coding for iron regulatory protein HFE contributes to hypertension, the TAMRISK study. Medicine (Baltimore). 2015;94(4):e464. 10.1097/MD.0000000000000464. 25634189 10.1097/MD.0000000000000464 PMC4602945

[241] Nikkari ST, Visto AL, Määttä KM, Kunnas TA. Minor variant of rs 16827043 in the iron regulator hemojuvelin gene (HJV) contributes to hypertension: the TAMRISK study. Medicine (Baltimore). 2017;96(5):e6052. 10.1097/MD.0000000000006052. 28151915 10.1097/MD.0000000000006052 PMC5293478

[242] Yang J, Wang M, Wang S, Li G, Gao Y. Study on ferroptosis pathway that operates in hypertensive brain damage. Clin Exp Hypertens. 2020;42(8):748–52. 10.1080/10641963.2020.1783545. 32564622 10.1080/10641963.2020.1783545

[243] Jin R, Yang R, Cui C, et al. Ferroptosis due to cystathionine γ lyase/hydrogen sulfide downregulation under high hydrostatic pressure exacerbates VSMC dysfunction. Front Cell Dev Biol. 2022;10:829316. 10.3389/fcell.2022.829316. 35186934 10.3389/fcell.2022.829316 PMC8850391

[244] Zhang X, Zheng C, Gao Z, et al. SLC7A11/xCT Prevents Cardiac Hypertrophy by Inhibiting Ferroptosis. Cardiovasc Drugs Ther. 2022;36(3):437–47. 10.1007/s10557-021-07220-z. 34259984 10.1007/s10557-021-07220-z

[245] Montezano AC, Dulak-Lis M, Tsiropoulou S, Harvey A, Briones AM, Touyz RM. Oxidative stress and human hypertension: vascular mechanisms, biomarkers, and novel therapies. Can J Cardiol. 2015;31(5):631–41. 10.1016/j.cjca.2015.02.008. 25936489 10.1016/j.cjca.2015.02.008

[246] Farooqui Z, Mohammad RS, Lokhandwala MF, Banday AA. Nrf2 inhibition induces oxidative stress, renal inflammation and hypertension in mice. Clin Exp Hypertens. 2021;43(2):175–80. 10.1080/10641963.2020.1836191. 33070655 10.1080/10641963.2020.1836191 PMC7790982

[247] Dikalov S, Itani H, Richmond B, et al. Tobacco smoking induces cardiovascular mitochondrial oxidative stress, promotes endothelial dysfunction, and enhances hypertension [published correction appears in Am J Physiol Heart Circ Physiol. 2019;316(4):H939. 10.1152/ajpheart.zh4-2748-corr.2019]. Am J Physiol Heart Circ Physiol. 2019;316(3):H639-H646. 10.1152/ajpheart.00595.2018. PMC6459311 30608177

[248] Hickey AJ, Chai CC, Choong SY, et al. Impaired ATP turnover and ADP supply depress cardiac mitochondrial respiration and elevate superoxide in nonfailing spontaneously hypertensive rat hearts. Am J Physiol Cell Physiol. 2009;297(3):C766–74. 10.1152/ajpcell.00111.200. 19553568 10.1152/ajpcell.00111.2009

[249] Dikalova AE, Pandey A, Xiao L, et al. Mitochondrial deacetylase Sirt3 reduces vascular dysfunction and hypertension while Sirt3 depletion in essential hypertension is linked to vascular inflammation and oxidative stress. Circ Res. 2020;126(4):439–52. 10.1161/CIRCRESAHA.119.315767. 31852393 10.1161/CIRCRESAHA.119.315767 PMC7035170

[250] Hu Y, Chi L, Kuebler WM, Goldenberg NM. Perivascular inflammation in pulmonary arterial hypertension. Cells. 2020;9(11):2338. 10.3390/cells9112338. 33105588 10.3390/cells9112338 PMC7690279

[251] Chen YH, Yuan W, Meng LK, Zhong JC, Liu XY. The role and mechanism of gut microbiota in pulmonary arterial hypertension. Nutrients. 2022;14(20):4278. 10.3390/nu14204278. 36296961 10.3390/nu14204278 PMC9610674

[252] Quatredeniers M, Mendes-Ferreira P, Santos-Ribeiro D, Nakhleh MK, Ghigna MR, Cohen-Kaminsky S, et al. Iron Deficiency in Pulmonary Arterial Hypertension: A Deep Dive into the Mechanisms. Cells. 2021;10(2):477. 10.3390/cells10020477. 33672218 10.3390/cells10020477 PMC7926484

[253] Callejo M, Barberá JA, Duarte J, Perez-Vizcaino F. Impact of nutrition on pulmonary arterial hypertension. Nutrients. 2020;12(1):169. 10.3390/nu12010169. 31936113 10.3390/nu12010169 PMC7019983

[254] Viethen T, Gerhardt F, Dumitrescu D, Knoop-Busch S, ten Freyhaus H, Rudolph TK, et al. Ferric carboxymaltose improves exercise capacity and quality of life in patients with pulmonary arterial hypertension and iron deficiency: a pilot study. Int J Cardiol. 2014;175(2):233–9. 10.1016/j.ijcard.2014.04.233. 24880481 10.1016/j.ijcard.2014.04.233

[255] Ruiter G, Manders E, Happé CM, Schalij I, Groepenhoff H, Howard LS, et al. Intravenous iron therapy in patients with idiopathic pulmonary arterial hypertension and iron deficiency. Pulm Circ. 2015;5(3):466–72. 10.1086/682217. 26401247 10.1086/682217 PMC4556497

[256] Lakhal-Littleton S, Crosby A, Frise MC, Mohammad G, Carr CA, Loick PAM, et al. Intracellular iron deficiency in pulmonary arterial smooth muscle cells induces pulmonary arterial hypertension in mice. Proc Natl Acad Sci U S A. 2019;116(26):13122–30. 10.1073/pnas.1822010116. 31152133 10.1073/pnas.1822010116 PMC6600981

[257] Xie SS, Deng Y, Guo SL, Li JQ, Zhou YC, Liao J, et al. Endothelial cell ferroptosis mediates monocrotaline-induced pulmonary hypertension in rats by modulating NLRP3 inflammasome activation. Sci Rep. 2022;12(1):3056. 10.1038/s41598-022-06848-7. 35197507 10.1038/s41598-022-06848-7 PMC8866506

[258] Wong CM, Preston IR, Hill NS, Suzuki YJ. Iron chelation inhibits the development of pulmonary vascular remodeling. Free Radic Biol Med. 2012;53(9):1738–47. 10.1016/j.freeradbiomed.2012.08.576. 22974762 10.1016/j.freeradbiomed.2012.08.576 PMC3472156

[259] Li B, Wang Z, Hong J, Che Y, Chen R, Hu Z, et al. Iron deficiency promotes aortic medial degeneration via destructing cytoskeleton of vascular smooth muscle cells. Clin Transl Med. 2021;11(1):e276. 10.1002/ctm2.276. 33463069 10.1002/ctm2.276 PMC7805404

[260] Shi F, Wang Z, Wu Q, Zhong X, Zhang M, Li B, et al. Iron deficiency promotes aortic media degeneration by activating endoplasmic reticulum stress-mediated IRE1 signaling pathway. Pharmacol Res. 2022;183:106366. 10.1016/j.phrs.2022.106366. 35882294 10.1016/j.phrs.2022.106366

[261] Li N, Yi X, He Y, Huo B, Chen Y, Zhang Z, et al. Targeting ferroptosis as a novel approach to alleviate aortic dissection. Int J Biol Sci. 2022;18(10):4118–34. 10.7150/ijbs.72528. 35844806 10.7150/ijbs.72528 PMC9274489

[262] Chen Y, Yi X, Huo B, He Y, Guo X, Zhang Z, et al. BRD4770 functions as a novel ferroptosis inhibitor to protect against aortic dissection. Pharmacol Res. 2022;177:106122. 10.1016/j.phrs.2022.106122. 35149187 10.1016/j.phrs.2022.106122

[263] Lau A, Tymianski M. Glutamate receptors, neurotoxicity and neurodegeneration. Pflugers Arch. 2010;460(2):525–42. 10.1007/s00424-010-0809-1. 20229265 10.1007/s00424-010-0809-1

[264] Li Q, Han X, Lan X, Gao Y, Wan J, Durham F, et al. Inhibition of neuronal ferroptosis protects hemorrhagic brain. JCI Insight. 2017;2(7):e90777. 10.1172/jci.insight.90777. 28405617 10.1172/jci.insight.90777 PMC5374066

[265] Guan X, Li X, Yang X, Yan J, Shi P, Ba L, et al. The neuroprotective effects of carvacrol on ischemia/reperfusion-induced hippocampal neuronal impairment by ferroptosis mitigation. Life Sci. 2019;235:116795. 10.1016/j.lfs.2019.116795. 31470002 10.1016/j.lfs.2019.116795

[266] Kontoghiorghes GJ, Eracleous E, Economides C, Kolnagou A. Advances in iron overload therapies. Prospects for effective use of deferiprone (L1), deferoxamine, the new experimental chelators ICL670, GT56-252, L1NA11 and their combinations. Curr Med Chem. 2005;12(23):2663–81. 10.2174/092986705774463003. 16305464 10.2174/092986705774463003

[267] Zille M, Karuppagounder SS, Chen Y, Gough PJ, Bertin J, Finger J, et al. Neuronal death after hemorrhagic stroke in vitro and in vivo shares features of ferroptosis and necroptosis. Stroke. 2017;48(4):1033–43. 10.1161/STROKEAHA.116.015609. 28250197 10.1161/STROKEAHA.116.015609 PMC5613764

[268] Yamada N, Karasawa T, Wakiya T, Sadatomo A, Ito H, Kamata R, et al. Iron overload as a risk factor for hepatic ischemia-reperfusion injury in liver transplantation: potential role of ferroptosis. Am J Transplant. 2020;20(6):1606–18. 10.1111/ajt.15773. 31909544 10.1111/ajt.15773

[269] Li J, Cao F, Yin HL, Huang ZJ, Lin ZT, Mao N, et al. Ferroptosis: past, present and future. Cell Death Dis. 2020;11(2):88. 10.1038/s41419-020-2298-2. 32015325 10.1038/s41419-020-2298-2 PMC6997353

[270] Qi J, Kim JW, Zhou Z, Lim CW, Kim B. Ferroptosis affects the progression of nonalcoholic steatohepatitis via the modulation of lipid peroxidation-mediated cell death in mice. Am J Pathol. 2020;190(1):68–81. 10.1016/j.ajpath.2019.09.011. 31610178 10.1016/j.ajpath.2019.09.011

[271] Porter JB, Taher AT, Cappellini MD, Vichinsky EP. Ethical issues and risk/benefit assessment of iron chelation therapy: advances with deferiprone/deferoxamine combinations and concerns about the safety, efficacy and costs of deferasirox [Kontoghiorghes GJ, Hemoglobin 2008;32(1-2):1-15.]. Hemoglobin. 2008;32(6):601–7. 10.1080/03630260802342008. 19065340 10.1080/03630260802342008

[272] Kontoghiorghes GJ. Design, properties, and effective use of the oral chelator L1 and other alpha-ketohydroxypyridines in the treatment of transfusional iron overload in thalassemia. Ann N Y Acad Sci. 1990;612:339–50. 10.1111/j.1749-6632.1990.tb24321.x. 2291562 10.1111/j.1749-6632.1990.tb24321.x

[273] Hider RC, Hoffbrand AV. The role of deferiprone in iron chelation. N Engl J Med. 2018;379(22):2140–50. 10.1056/NEJMra1800219. 30485781 10.1056/NEJMra1800219

[274] Fredenburg AM, Sethi RK, Allen DD, Yokel RA. The pharmacokinetics and blood-brain barrier permeation of the chelators 1,2 dimethyl-, 1,2 diethyl-, and 1-[ethan-1′ ol]-2-methyl-3-hydroxypyridin-4-one in the rat. Toxicology. 1996;108(3):191–9. 10.1016/0300-483x(95)03301-u. 8658538 10.1016/0300-483x(95)03301-u

[275] Kakhlon O, Manning H, Breuer W, Melamed-Book N, Lu C, Cortopassi G, et al. Cell functions impaired by frataxin deficiency are restored by drug-mediated iron relocation. Blood. 2008;112(13):5219–27. 10.1182/blood-2008-06-161919. 18796625 10.1182/blood-2008-06-161919

[276] Ichikawa Y, Ghanefar M, Bayeva M, Wu R, Khechaduri A, Naga Prasad SV, et al. Cardiotoxicity of doxorubicin is mediated through mitochondrial iron accumulation. J Clin Invest. 2014;124(2):617–30. 10.1172/JCI72931. 24382354 10.1172/JCI72931 PMC3904631

[277] Eberhard Y, McDermott SP, Wang X, Gronda M, Venugopal A, Wood TE, et al. Chelation of intracellular iron with the antifungal agent ciclopirox olamine induces cell death in leukemia and myeloma cells. Blood. 2009;114(14):3064–73. 10.1182/blood-2009-03-209965. 19589922 10.1182/blood-2009-03-209965

[278] Shen T, Huang S. Repositioning the old fungicide ciclopirox for new medical uses. Curr Pharm Des. 2016;22(28):4443–50. 10.2174/1381612822666160530151209. 27238364 10.2174/1381612822666160530151209 PMC6623967

[279] Feng H, Schorpp K, Jin J, Yozwiak CE, Hoffstrom BG, Decker AM, et al. Transferrin receptor is a specific ferroptosis marker. Cell Rep. 2020;30(10):3411-3423.e7. 10.1016/j.celrep.2020.02.049. 32160546 10.1016/j.celrep.2020.02.049 PMC7172030

[280] Horonchik L, Wessling-Resnick M. The small-molecule iron transport inhibitor ferristatin/NSC306711 promotes degradation of the transferrin receptor. Chem Biol. 2008;15(7):647–53. 10.1016/j.chembiol.2008.05.011. 18635001 10.1016/j.chembiol.2008.05.011 PMC3747564

[281] Fleming MD, Romano MA, Su MA, Garrick LM, Garrick MD, Andrews NC. Nramp2 is mutated in the anemic Belgrade (b) rat: evidence of a role for Nramp2 in endosomal iron transport. Proc Natl Acad Sci U S A. 1998;95(3):1148–53. 10.1073/pnas.95.3.1148. 9448300 10.1073/pnas.95.3.1148 PMC18702

[282] Wetli HA, Buckett PD, Wessling-Resnick M. Small-molecule screening identifies the selanazal drug ebselen as a potent inhibitor of DMT1-mediated iron uptake. Chem Biol. 2006;13(9):965–72. 10.1016/j.chembiol.2006.08.005. 16984886 10.1016/j.chembiol.2006.08.005 PMC2542486

[283] Zhang Z, Kodumuru V, Sviridov S, Liu S, Chafeev M, Chowdhury S, et al. Discovery of benzylisothioureas as potent divalent metal transporter 1 (DMT1) inhibitors. Bioorg Med Chem Lett. 2012;22(15):5108–13. 10.1016/j.bmcl.2012.05.129. 22749870 10.1016/j.bmcl.2012.05.129

[284] Donovan A, Lima CA, Pinkus JL, Pinkus GS, Zon LI, Robine S, et al. The iron exporter ferroportin/Slc40a1 is essential for iron homeostasis. Cell Metab. 2005;1(3):191–200. 10.1016/j.cmet.2005.01.003. 16054062 10.1016/j.cmet.2005.01.003

[285] Nemeth E, Tuttle MS, Powelson J, Vaughn MB, Donovan A, Ward DM, et al. Hepcidin regulates cellular iron efflux by binding to ferroportin and inducing its internalization. Science. 2004;306(5704):2090–3. 10.1126/science.1104742. 15514116 10.1126/science.1104742

[286] Katsarou A, Pantopoulos K. Hepcidin therapeutics. Pharm Basel Switz. 2018;11(4):127. 10.3390/ph11040127. 10.3390/ph11040127 PMC6316648 30469435

[287] Chung B, Verdier F, Matak P, Deschemin JC, Mayeux P, Vaulont S. Oncostatin M is a potent inducer of hepcidin, the iron regulatory hormone. FASEB J Off Publ Fed Am Soc Exp Biol. 2010;24(6):2093–103. 10.1096/fj.09-152561. 10.1096/fj.09-152561 20124431

[288] Boyce M, Warrington S, Cortezi B, Zöllner S, Vauléon S, Swinkels DW, et al. Safety, pharmacokinetics and pharmacodynamics of the anti-hepcidin Spiegelmer lexaptepid pegol in healthy subjects. Br J Pharmacol. 2016;173(10):1580–8. 10.1111/bph.13433. 26773325 10.1111/bph.13433 PMC4842915

[289] Vadhan-Raj S, Abonour R, Goldman JW, Smith DA, Slapak CA, Ilaria RL, et al. A first-in-human phase 1 study of a hepcidin monoclonal antibody, LY2787106, in cancer-associated anemia. J Hematol OncolJ Hematol Oncol. 2017;10(1):73. 10.1186/s13045-017-0427-x. 28327200 10.1186/s13045-017-0427-x PMC5361694

[290] Mancias JD, Wang X, Gygi SP, Harper JW, Kimmelman AC. Quantitative proteomics identifies NCOA4 as the cargo receptor mediating ferritinophagy. Nature. 2014;509(7498):105–9. 10.1038/nature13148. 24695223 10.1038/nature13148 PMC4180099

[291] Hou W, Xie Y, Song X, Sun X, Lotze MT, Zeh HJ, et al. Autophagy promotes ferroptosis by degradation of ferritin. Autophagy. 2016;12(8):1425–8. 10.1080/15548627.2016.1187366. 27245739 10.1080/15548627.2016.1187366 PMC4968231

[292] Gao M, Monian P, Pan Q, Zhang W, Xiang J, Jiang X. Ferroptosis is an autophagic cell death process. Cell Res. 2016;26(9):1021–32. 10.1038/cr.2016.95. 27514700 10.1038/cr.2016.95 PMC5034113

[293] Fang Y, Chen X, Tan Q, Zhou H, Xu J, Gu Q. Inhibiting ferroptosis through disrupting the NCOA4-FTH1 interaction: a new mechanism of action. ACS Cent Sci. 2021;7(6):980–9. 10.1021/acscentsci.0c01592. 34235259 10.1021/acscentsci.0c01592 PMC8227600

[294] Suttner DM, Dennery PA. Reversal of HO-1 related cytoprotection with increased expression is due to reactive iron. FASEB J Off Publ Fed Am Soc Exp Biol. 1999;13(13):1800–9. 10.1096/fasebj.13.13.1800. 10.1096/fasebj.13.13.1800 10506583

[295] Hassannia B, Vandenabeele P, Vanden Berghe T. Targeting ferroptosis to iron out cancer. Cancer Cell. 2019;35(6):830–49. 10.1016/j.ccell.2019.04.002. 31105042 10.1016/j.ccell.2019.04.002

[296] Vreman HJ, Ekstrand BC, Stevenson DK. Selection of metalloporphyrin heme oxygenase inhibitors based on potency and photoreactivity. Pediatr Res. 1993;33(2):195–200. 10.1203/00006450-199302000-00021. 8433895 10.1203/00006450-199302000-00021

[297] Morisawa T, Wong RJ, Bhutani VK, Vreman HJ, Stevenson DK. Inhibition of heme oxygenase activity in newborn mice by azalanstat. Can J Physiol Pharmacol. 2008;86(10):651–9. 10.1139/y08-069. 18841169 10.1139/y08-069

[298] Kuhn H, Saam J, Eibach S, Holzhütter HG, Ivanov I, Walther M. Structural biology of mammalian lipoxygenases: enzymatic consequences of targeted alterations of the protein structure. Biochem Biophys Res Commun. 2005;338(1):93–101. 10.1016/j.bbrc.2005.08.238. 16168952 10.1016/j.bbrc.2005.08.238

[299] Angeli JPF, Shah R, Pratt DA, Conrad M. Ferroptosis inhibition: mechanisms and opportunities. Trends Pharmacol Sci. 2017;38(5):489–98. 10.1016/j.tips.2017.02.005. 28363764 10.1016/j.tips.2017.02.005

[300] Haeggström JZ, Funk CD. Lipoxygenase and leukotriene pathways: biochemistry, biology, and roles in disease. Chem Rev. 2011;111(10):5866–98. 10.1021/cr200246d. 21936577 10.1021/cr200246d

[301] Liu Y, Wang W, Li Y, Xiao Y, Cheng J, Jia J. The 5-lipoxygenase inhibitor zileuton confers neuroprotection against glutamate oxidative damage by inhibiting ferroptosis. Biol Pharm Bull. 2015;38(8):1234–9. 10.1248/bpb.b15-00048. 26235588 10.1248/bpb.b15-00048

[302] Luci DK, Jameson JB, Yasgar A, Diaz G, Joshi N, Kantz A, et al. Synthesis and structure-activity relationship studies of 4-((2-hydroxy-3-methoxybenzyl)amino)benzenesulfonamide derivatives as potent and selective inhibitors of 12-lipoxygenase. J Med Chem. 2014;57(2):495–506. 10.1021/jm4016476. 24393039 10.1021/jm4016476 PMC3967794

[303] Adili R, Tourdot BE, Mast K, Yeung J, Freedman JC, Green A, et al. First Selective 12-LOX Inhibitor, ML355, Impairs Thrombus Formation and Vessel Occlusion In Vivo With Minimal Effects on Hemostasis. Arterioscler Thromb Vasc Biol. 2017;37(10):1828–39. 10.1161/ATVBAHA.117.309868. 28775075 10.1161/ATVBAHA.117.309868 PMC5620123

[304] Ma K, Xiao A, Park SH, Glenn L, Jackson L, Barot T, et al. 12-lipoxygenase inhibitor improves functions of cytokine-treated human islets and type 2 diabetic islets. J Clin Endocrinol Metab. 2017;102(8):2789–97. 10.1210/jc.2017-00267. 28609824 10.1210/jc.2017-00267 PMC5546865

[305] Zhao J, Wu Y, Liang S, Piao X. Activation of SSAT1/ALOX15 axis aggravates cerebral ischemia/reperfusion injury via triggering neuronal ferroptosis. Neuroscience. 2022;485:78–90. 10.1016/j.neuroscience.2022.01.017. 35090880 10.1016/j.neuroscience.2022.01.017

[306] Ma XH, Liu JHZ, Liu CY, Sun WY, Duan WJ, Wang G, et al. ALOX15-launched PUFA-phospholipids peroxidation increases the susceptibility of ferroptosis in ischemia-induced myocardial damage. Signal Transduct Target Ther. 2022;7(1):288. 10.1038/s41392-022-01090-z. 35970840 10.1038/s41392-022-01090-z PMC9378747

[307] Cai W, Liu L, Shi X, Liu Y, Wang J, Fang X, et al. Alox15/15-HpETE aggravates myocardial ischemia-reperfusion injury by promoting cardiomyocyte ferroptosis. Circulation. 2023;147(19):1444–60. 10.1161/CIRCULATIONAHA.122.060257. 36987924 10.1161/CIRCULATIONAHA.122.060257

[308] Skouta R, Dixon SJ, Wang J, Dunn DE, Orman M, Shimada K, et al. Ferrostatins inhibit oxidative lipid damage and cell death in diverse disease models. J Am Chem Soc. 2014;136(12):4551–6. 10.1021/ja411006a. 24592866 10.1021/ja411006a PMC3985476

[309] Linkermann A, Skouta R, Himmerkus N, Mulay SR, Dewitz C, De Zen F, et al. Synchronized renal tubular cell death involves ferroptosis. Proc Natl Acad Sci U S A. 2014;111(47):16836–41. 10.1073/pnas.1415518111. 25385600 10.1073/pnas.1415518111 PMC4250130

[310] Devisscher L, Van Coillie S, Hofmans S, Van Rompaey D, Goossens K, Meul E, et al. Discovery of novel, drug-like ferroptosis inhibitors with in vivo efficacy. J Med Chem. 2018;61(22):10126–40. 10.1021/acs.jmedchem.8b01299. 30354101 10.1021/acs.jmedchem.8b01299

[311] Hanthorn JJ, Valgimigli L, Pratt DA. Incorporation of ring nitrogens into diphenylamine antioxidants: striking a balance between reactivity and stability. J Am Chem Soc. 2012;134(20):8306–9. 10.1021/ja300086z. 22369282 10.1021/ja300086z

[312] Shah R, Margison K, Pratt DA. The potency of diarylamine radical-trapping antioxidants as inhibitors of ferroptosis underscores the role of autoxidation in the mechanism of cell death. ACS Chem Biol. 2017;12(10):2538–45. 10.1021/acschembio.7b00730. 28837769 10.1021/acschembio.7b00730

[313] Zilka O, Shah R, Li B, Friedmann Angeli JP, Griesser M, Conrad M, et al. On the mechanism of cytoprotection by ferrostatin-1 and liproxstatin-1 and the role of lipid peroxidation in ferroptotic cell death. ACS Cent Sci. 2017;3(3):232–43. 10.1021/acscentsci.7b00028. 28386601 10.1021/acscentsci.7b00028 PMC5364454

[314] Li J, Lama R, Galster SL, Inigo JR, Wu J, Chandra D, et al. Small-Molecule MMRi62 Induces Ferroptosis and Inhibits Metastasis in Pancreatic Cancer via Degradation of Ferritin Heavy Chain and Mutant p53. Mol Cancer Ther. 2022;21(4):535–45. 10.1158/1535-7163.MCT-21-0728. 35131878 10.1158/1535-7163.MCT-21-0728 PMC10258866

[315] Homma T, Kobayashi S, Sato H, Fujii J. Edaravone, a free radical scavenger, protects against ferroptotic cell death in vitro. Exp Cell Res. 2019;384(1):111592. 10.1016/j.yexcr.2019.111592. 31479686 10.1016/j.yexcr.2019.111592

[316] Kagan VE, Mao G, Qu F, Angeli JPF, Doll S, Croix CS, et al. Oxidized arachidonic and adrenic PEs navigate cells to ferroptosis. Nat Chem Biol. 2017;13(1):81–90. 10.1038/nchembio.2238. 27842066 10.1038/nchembio.2238 PMC5506843

[317] Yang WS, Kim KJ, Gaschler MM, Patel M, Shchepinov MS, Stockwell BR. Peroxidation of polyunsaturated fatty acids by lipoxygenases drives ferroptosis. Proc Natl Acad Sci U S A. 2016;113(34):E4966-4975. 10.1073/pnas.1603244113. 27506793 10.1073/pnas.1603244113 PMC5003261

[318] Shah R, Shchepinov MS, Pratt DA. Resolving the role of lipoxygenases in the initiation and execution of ferroptosis. ACS Cent Sci. 2018;4(3):387–96. 10.1021/acscentsci.7b00589. 29632885 10.1021/acscentsci.7b00589 PMC5879472

[319] Magtanong L, Ko PJ, To M, Cao JY, Forcina GC, Tarangelo A, et al. Exogenous Monounsaturated Fatty Acids Promote a Ferroptosis-Resistant Cell State. Cell Chem Biol. 2019;26(3):420-432.e9. 10.1016/j.chembiol.2018.11.016. 30686757 10.1016/j.chembiol.2018.11.016 PMC6430697

[320] Michaelis M, Geiler J, Naczk P, Sithisarn P, Leutz A, Doerr HW, et al. Glycyrrhizin exerts antioxidative effects in H5N1 influenza A virus-infected cells and inhibits virus replication and pro-inflammatory gene expression. PLoS ONE. 2011;6(5):e19705. 10.1371/journal.pone.0019705. 21611183 10.1371/journal.pone.0019705 PMC3096629

[321] Monti DA, Zabrecky G, Kremens D, Liang TW, Wintering NA, Cai J, et al. N-Acetyl Cysteine May Support Dopamine Neurons in Parkinson’s Disease: Preliminary Clinical and Cell Line Data. PLoS ONE. 2016;11(6):e0157602. 10.1371/journal.pone.0157602. 27309537 10.1371/journal.pone.0157602 PMC4911055

[322] Ates B, Abraham L, Ercal N. Antioxidant and free radical scavenging properties of N-acetylcysteine amide (NACA) and comparison with N-acetylcysteine (NAC). Free Radic Res. 2008;42(4):372–7. 10.1080/10715760801998638. 18404536 10.1080/10715760801998638

[323] He R, Zheng W, Ginman T, Ottosson H, Norgren S, Zhao Y, et al. Pharmacokinetic profile of N-acetylcysteine amide and its main metabolite in mice using new analytical method. Eur J Pharm Sci. 2020;143:105158. 10.1016/j.ejps.2019.105158. 31740394 10.1016/j.ejps.2019.105158

[324] Ingold I, Berndt C, Schmitt S, Doll S, Poschmann G, Buday K, et al. Selenium Utilization by GPX4 Is Required to Prevent Hydroperoxide-Induced Ferroptosis. Cell. 2018;172(3):409-422.e21. 10.1016/j.cell.2017.11.048. 29290465 10.1016/j.cell.2017.11.048

[325] Hatfield DL, Gladyshev VN. How selenium has altered our understanding of the genetic code. Mol Cell Biol. 2002;22(11):3565–76. 10.1128/MCB.22.11.3565-3576.2002. 11997494 10.1128/MCB.22.11.3565-3576.2002 PMC133838

[326] Alim I, Caulfield JT, Chen Y, Swarup V, Geschwind DH, Ivanova E, et al. Selenium drives a transcriptional adaptive program to block ferroptosis and treat stroke. Cell. 2019;177(5):1262-1279.e25. 10.1016/j.cell.2019.03.032. 31056284 10.1016/j.cell.2019.03.032

[327] Wang D, Peng Y, Xie Y, Zhou B, Sun X, Kang R, et al. Antiferroptotic activity of non-oxidative dopamine. Biochem Biophys Res Commun. 2016;480(4):602–7. 10.1016/j.bbrc.2016.10.099. 27793671 10.1016/j.bbrc.2016.10.099

[328] Li C, Deng X, Zhang W, Xie X, Conrad M, Liu Y, et al. Novel Allosteric Activators for Ferroptosis Regulator Glutathione Peroxidase 4. J Med Chem. 2019;62(1):266–75. 10.1021/acs.jmedchem.8b00315. 29688708 10.1021/acs.jmedchem.8b00315

[329] Wu M, Xu LG, Li X, Zhai Z, Shu HB. AMID, an apoptosis-inducing factor-homologous mitochondrion-associated protein, induces caspase-independent apoptosis. J Biol Chem. 2002;277(28):25617–23. 10.1074/jbc.M202285200. 11980907 10.1074/jbc.M202285200

[330] Fang Y, Tan Q, Zhou H, Gu Q, Xu J. Discovery of novel diphenylbutene derivative ferroptosis inhibitors as neuroprotective agents. Eur J Med Chem. 2022;231:114151. 10.1016/j.ejmech.2022.114151. 35123296 10.1016/j.ejmech.2022.114151

[331] Hensley CT, Wasti AT, DeBerardinis RJ. Glutamine and cancer: cell biology, physiology, and clinical opportunities. J Clin Invest. 2013;123(9):3678–84. 10.1172/JCI69600. 23999442 10.1172/JCI69600 PMC3754270

[332] Kong L, Deng J, Zhou X, Cai B, Zhang B, Chen X, et al. Sitagliptin activates the p62-Keap1-Nrf2 signalling pathway to alleviate oxidative stress and excessive autophagy in severe acute pancreatitis-related acute lung injury. Cell Death Dis. 2021;12(10):928. 10.1038/s41419-021-04227-0. 34635643 10.1038/s41419-021-04227-0 PMC8505515

[333] Chen Y, Li X, Wang S, Miao R, Zhong J. Targeting iron metabolism and ferroptosis as novel therapeutic approaches in cardiovascular diseases. Nutrients. 2023;15(3):591. 10.3390/nu15030591. 36771298 10.3390/nu15030591 PMC9921472

[334] Chen Y, Zhang P, Chen W, Chen G. Ferroptosis mediated DSS-induced ulcerative colitis associated with Nrf2/HO-1 signaling pathway. Immunol Lett. 2020;225:9–15. 10.1016/j.imlet.2020.06.005. 32540488 10.1016/j.imlet.2020.06.005

[335] Sauerbeck A, Schonberg DL, Laws JL, McTigue DM. Systemic iron chelation results in limited functional and histological recovery after traumatic spinal cord injury in rats. Exp Neurol. 2013;248:53–61. 10.1016/j.expneurol.2013.05.011. 23712107 10.1016/j.expneurol.2013.05.011 PMC5503200

[336] Imai T, Tsuji S, Matsubara H, Ohba T, Sugiyama T, Nakamura S, et al. Deferasirox, a trivalent iron chelator, ameliorates neuronal damage in hemorrhagic stroke models. Naunyn Schmiedebergs Arch Pharmacol. 2021;394(1):73–84. 10.1007/s00210-020-01963-6. 32808069 10.1007/s00210-020-01963-6

[337] Li T, Tan Y, Ouyang S, He J, Liu L. Resveratrol protects against myocardial ischemia-reperfusion injury via attenuating ferroptosis. Gene. 2022;808:145968. 10.1016/j.gene.2021.145968. 34530090 10.1016/j.gene.2021.145968

[338] Peng Y, Liao B, Zhou Y, Zeng W, Zeng ZY. Atorvastatin Inhibits Ferroptosis of H9C2 Cells by regulatingSMAD7/Hepcidin Expression to Improve Ischemia-Reperfusion Injury. Cardiol Res Pract. 2022;2022:3972829. 10.1155/2022/3972829. 36398315 10.1155/2022/3972829 PMC9666047

[339] Patel HH, Fryer RM, Gross ER, Bundey RA, Hsu AK, Isbell M, et al. 12-lipoxygenase in opioid-induced delayed cardioprotection: gene array, mass spectrometric, and pharmacological analyses. Circ Res. 2003;92(6):676–82. 10.1161/01.RES.0000065167.52922.F6. 12623876 10.1161/01.RES.0000065167.52922.F6

[340] van Leyen K, Kim HY, Lee SR, Jin G, Arai K, Lo EH. Baicalein and 12/15-lipoxygenase in the ischemic brain. Stroke. 2006;37(12):3014–8. 10.1161/01.STR.0000249004.25444.a5. 17053180 10.1161/01.STR.0000249004.25444.a5

[341] Fan Z, Cai L, Wang S, Wang J, Chen B. Baicalin Prevents Myocardial Ischemia/Reperfusion Injury Through Inhibiting ACSL4 Mediated Ferroptosis. Front Pharmacol. 2021;12:628988. 10.3389/fphar.2021.628988. 33935719 10.3389/fphar.2021.628988 PMC8079950

[342] Lin JH, Yang KT, Ting PC, Luo YP, Lin DJ, Wang YS, et al. Gossypol Acetic Acid Attenuates Cardiac Ischemia/Reperfusion Injury in Rats via an Antiferroptotic Mechanism. Biomolecules. 2021;11(11):1667. 10.3390/biom11111667. 34827665 10.3390/biom11111667 PMC8615989

[343] Pan Y, Wang X, Liu X, Shen L, Chen Q, Shu Q. Targeting Ferroptosis as a Promising Therapeutic Strategy for Ischemia-Reperfusion Injury. Antioxid Basel Switz. 2022;11(11):2196. 10.3390/antiox11112196. 10.3390/antiox11112196 PMC9686892 36358568

[344] Lin JH, Yang KT, Lee WS, Ting PC, Luo YP, Lin DJ, et al. Xanthohumol protects the rat myocardium against ischemia/reperfusion injury-induced ferroptosis. Oxid Med Cell Longev. 2022;2022:9523491. 10.1155/2022/9523491. 35082973 10.1155/2022/9523491 PMC8786462

[345] Qian W, Liu D, Han Y, Liu M, Liu B, Ji Q, et al. Cyclosporine A-loaded apoferritin alleviates myocardial ischemia-reperfusion injury by simultaneously blocking ferroptosis and apoptosis of cardiomyocytes. Acta Biomater. 2023;160:265–80. 10.1016/j.actbio.2023.02.025. 36822483 10.1016/j.actbio.2023.02.025

[346] Lu H, Xiao H, Dai M, Xue Y, Zhao R. Britanin relieves ferroptosis-mediated myocardial ischaemia/reperfusion damage by upregulating GPX4 through activation of AMPK/GSK3β/Nrf2 signalling. Pharm Biol. 2022;60(1):38–45. 10.1080/13880209.2021.2007269. 34860639 10.1080/13880209.2021.2007269 PMC8648013

[347] Li YC, Cheng ML. Carvedilol confers ferroptosis resistance in HL-1 cells by upregulating GPX4, FTH1, and FTL1 and inducing metabolic remodeling under hypoxia/reoxygenation. Antioxidants. 2024;14(1):7. 10.3390/antiox14010007. 39857341 10.3390/antiox14010007 PMC11762394

[348] Chen W, Zhang Y, Wang Z, Tan M, Lin J, Qian X, et al. Dapagliflozin alleviates myocardial ischemia/reperfusion injury by reducing ferroptosis via MAPK signaling inhibition. Front Pharmacol. 2023;14:1078205. 10.3389/fphar.2023.1078205. 36891270 10.3389/fphar.2023.1078205 PMC9986553

[349] Wang Z, Yao M, Jiang L, Wang L, Yang Y, Wang Q, et al. Dexmedetomidine attenuates myocardial ischemia/reperfusion-induced ferroptosis via AMPK/GSK-3β/Nrf2 axis. Biomed Pharmacother. 2022;154:113572. 10.1016/j.biopha.2022.113572. 35988428 10.1016/j.biopha.2022.113572

[350] Dang R, Wang M, Li X, Wang H, Liu L, Wu Q, et al. Edaravone ameliorates depressive and anxiety-like behaviors via Sirt1/Nrf2/HO-1/Gpx4 pathway. J Neuroinflammation. 2022;19(1):41. 10.1186/s12974-022-02400-6. 35130906 10.1186/s12974-022-02400-6 PMC8822843

[351] Zeng Y, Zhu G, Zhu M, Song J, Cai H, Song Y, et al. Edaravone attenuated particulate matter-induced lung inflammation by inhibiting ROS-NF-κB signaling pathway. Oxid Med Cell Longev. 2022;2022:6908884. 10.1155/2022/6908884. 35502210 10.1155/2022/6908884 PMC9056219

[352] Lv Z, Wang F, Zhang X, Zhang X, Zhang J, Liu R. Etomidate attenuates the ferroptosis in myocardial ischemia/reperfusion rat model via Nrf2/HO-1 pathway. Shock. 2021;56(3):440–9. 34091586 10.1097/SHK.0000000000001751

[353] Liu X, Qi K, Gong Y, Long X, Zhu S, Lu F, et al. Ferulic acid alleviates myocardial ischemia reperfusion injury via upregulating AMPKα2 expression-mediated ferroptosis depression. J Cardiovasc Pharmacol. 2021;79(4):489–500. 10.1097/FJC.0000000000001199. 34935700 10.1097/FJC.0000000000001199 PMC8983949

[354] Hwang JW, Park JH, Park BW, Kim H, Kim JJ, Sim WS, et al. Histochrome attenuates myocardial ischemia-reperfusion injury by inhibiting ferroptosis-induced cardiomyocyte death. Antioxidants. 2021;10(10):1624. 10.3390/antiox10101624. 34679760 10.3390/antiox10101624 PMC8533175

[355] Feng Y, Madungwe NB, Imam Aliagan AD, Tombo N, Bopassa JC. Liproxstatin-1 protects the mouse myocardium against ischemia/reperfusion injury by decreasing VDAC1 levels and restoring GPX4 levels. Biochem Biophys Res Commun. 2019;520(3):606–11. 10.1016/j.bbrc.2019.10.006. 31623831 10.1016/j.bbrc.2019.10.006 PMC7457545

[356] Karuppagounder SS, Alin L, Chen Y, Brand D, Bourassa MW, Dietrich K, et al. N-acetylcysteine targets 5 lipoxygenase-derived, toxic lipids and can synergize with prostaglandin E2 to inhibit ferroptosis and improve outcomes following hemorrhagic stroke in mice. Ann Neurol. 2018;84(6):854–72. 10.1002/ana.25356. 30294906 10.1002/ana.25356 PMC6519209

[357] Li Q, Liao J, Chen W, Zhang K, Li H, Ma F, et al. NAC alleviative ferroptosis in diabetic nephropathy via maintaining mitochondrial redox homeostasis through activating SIRT3-SOD2/Gpx4 pathway. Free Radic Biol Med. 2022;187:158–70. 10.1016/j.freeradbiomed.2022.05.024. 35660452 10.1016/j.freeradbiomed.2022.05.024

[358] Huang J, Xie H, Yang Y, Chen L, Lin T, Wang B, et al. The role of ferroptosis and endoplasmic reticulum stress in intermittent hypoxia-induced myocardial injury. Sleep Breath. 2023;27(3):1005–11. 10.1007/s11325-022-02692-1. 35951213 10.1007/s11325-022-02692-1

[359] Xu S, Wu B, Zhong B, Lin L, Ding Y, Jin X, et al. Naringenin alleviates myocardial ischemia/reperfusion injury by regulating the nuclear factor-erythroid factor 2-related factor 2 (Nrf2) /System xc-/ glutathione peroxidase 4 (GPX4) axis to inhibit ferroptosis. Bioengineered. 2021;12(2):10924–34. 10.1080/21655979.2021.1995994. 34699317 10.1080/21655979.2021.1995994 PMC8809912

[360] Li S, Lei Z, Yang X, Zhao M, Hou Y, Wang D, et al. Propofol Protects Myocardium From Ischemia/Reperfusion Injury by Inhibiting Ferroptosis Through the AKT/p53 Signaling Pathway. Front Pharmacol. 2022;13:841410. 10.3389/fphar.2022.841410. 35370724 10.3389/fphar.2022.841410 PMC8966655

[361] Wu S, Zhu J, Wu G, Hu Z, Ying P, Bao Z, et al. 6-Gingerol alleviates ferroptosis and inflammation of diabetic cardiomyopathy via the Nrf2/HO-1 pathway. Oxid Med Cell Longev. 2022;2022:3027514. 10.1155/2022/3027514. 36624878 10.1155/2022/3027514 PMC9825225

[362] Mei SL, Xia ZY, Qiu Z, Jia YF, Zhou JJ, Zhou B. Shenmai injection attenuates myocardial ischemia/reperfusion injury by targeting Nrf2/GPX4 signalling-mediated ferroptosis. Chin J Integr Med. 2022;28(11):983–91. 10.1007/s11655-022-3620-x. 35997859 10.1007/s11655-022-3620-x

[363] Mishra D, Jain N, Rajoriya V, Jain AK. Glycyrrhizin conjugated chitosan nanoparticles for hepatocyte-targeted delivery of lamivudine. J Pharm Pharmacol. 2014;66(8):1082–93. 10.1111/jphp.12235. 24641311 10.1111/jphp.12235

[364] Ojha S, Javed H, Azimullah S, Abul Khair SB, Haque ME. Glycyrrhizic acid attenuates neuroinflammation and oxidative stress in rotenone model of Parkinson’s disease. Neurotox Res. 2016;29(2):275–87. 10.1007/s12640-015-9579-z. 26607911 10.1007/s12640-015-9579-z

[365] Wang XR, Hao HG, Chu L. Glycyrrhizin inhibits LPS-induced inflammatory mediator production in endometrial epithelial cells. Microb Pathog. 2017;109:110–3. 10.1016/j.micpath.2017.05.032. 28552807 10.1016/j.micpath.2017.05.032

[366] Wang Y, Chen Q, Shi C, Jiao F, Gong Z. Mechanism of glycyrrhizin on ferroptosis during acute liver failure by inhibiting oxidative stress. Mol Med Rep. 2019;20(5):4081–90. 10.3892/mmr.2019.10660. 31545489 10.3892/mmr.2019.10660 PMC6797988

[367] Huang Y, Tsang SY, Yao X, Chen ZY. Biological properties of baicalein in cardiovascular system. Curr Drug Targets Cardiovasc Haematol Disord. 2005;5(2):177–84. 10.2174/1568006043586206. 15853750 10.2174/1568006043586206

[368] Afanas’ev IB, Dorozhko AI, Brodskii AV, Kostyuk VA, Potapovitch AI. Chelating and free radical scavenging mechanisms of inhibitory action of rutin and quercetin in lipid peroxidation. Biochem Pharmacol. 1989;38(11):1763–9. 10.1016/0006-2952(89)90410-3. 2735934 10.1016/0006-2952(89)90410-3

[369] Xie Y, Song X, Sun X, Huang J, Zhong M, Lotze MT, et al. Identification of baicalein as a ferroptosis inhibitor by natural product library screening. Biochem Biophys Res Commun. 2016;473(4):775–80. 10.1016/j.bbrc.2016.03.052. 27037021 10.1016/j.bbrc.2016.03.052

[370] Polystyrene Nanoparticles Reduced ROS and Inhibited Ferroptosis by Triggering Lysosome Stress and TFEB Nucleus Translocation in a Size-Dependent Manner - PubMed [Internet]. Available from: https://pubmed.ncbi.nlm.nih.gov/31558022/. Cited 7 Sept 2025.

[371] Dharmalingam P, Talakatta G, Mitra J, Wang H, Derry PJ, Nilewski LG, et al. Pervasive genomic damage in experimental intracerebral hemorrhage: therapeutic potential of a mechanistic-based carbon nanoparticle. ACS Nano. 2020;14(3):2827–46. 10.1021/acsnano.9b05821. 32049495 10.1021/acsnano.9b05821 PMC7850811

[372] Y L, C L, B F, X C, K W, H X, et al. Ferroptosis, a therapeutic target for cardiovascular diseases, neurodegenerative diseases and cancer. J Transl Med [Internet]. 2024;22(1). Available from: https://pubmed.ncbi.nlm.nih.gov/39710702/10.1186/s12967-024-05881-6. Cited 4 Nov 2025. 10.1186/s12967-024-05881-6 PMC11663363 39710702

[373] L Z, Yl L, Y X, Xy B, Rr Q, X Z, et al. Ferroptosis inhibitors: past, present and future. Front Pharmacol [Internet]. 2024;15. Available from: https://pubmed.ncbi.nlm.nih.gov/38846099/10.3389/fphar.2024.1407335. Cited 4 Nov 2025. 10.3389/fphar.2024.1407335 PMC11153831 38846099

[374] Y W, S L, W L, J W, X H, T T, et al. Cardiac-targeted and ROS-responsive liposomes containing puerarin for attenuating myocardial ischemia-reperfusion injury. Nanomed [Internet]. 2024;19(28). Available from: https://pubmed.ncbi.nlm.nih.gov/39316570/10.1080/17435889.2024.2402678. Cited 4 Nov 2025. 10.1080/17435889.2024.2402678 PMC11492708 39316570

[375] Hofmans S, Vanden Berghe T, Devisscher L, Hassannia B, Lyssens S, Joossens J, et al. Novel ferroptosis inhibitors with improved potency and ADME properties. J Med Chem. 2016;59(5):2041–53. 10.1021/acs.jmedchem.5b01641. 26696014 10.1021/acs.jmedchem.5b01641

[376] Li S, Li F, Wang Y, Li W, Wu J, Hu X, et al. Multiple delivery strategies of nanocarriers for myocardial ischemia-reperfusion injury: current strategies and future prospective. Drug Deliv. 2024;31(1):2298514. 10.1080/10717544.2023.2298514. 38147501 10.1080/10717544.2023.2298514 PMC10763895

[377] Luan Y, Yang Y, Luan Y, Liu H, Xing H, Pei J, et al. Targeting ferroptosis and ferritinophagy: new targets for cardiovascular diseases. J Zhejiang University-SCIENCE B. 2024;25(1):1–22. 10.1631/jzus.B2300097. 10.1631/jzus.B2300097 PMC10758208 38163663

[378] Hadian K, Stockwell BR. A roadmap to creating ferroptosis-based medicines. Nat Chem Biol. 2021;17(11):1113–6. 10.1038/s41589-021-00853-z. 34675413 10.1038/s41589-021-00853-z PMC8990224

[379] Fratta Pasini AM, Stranieri C, Busti F, Di Leo EG, Girelli D, Cominacini L. New insights into the role of ferroptosis in cardiovascular diseases. Cells. 2023;12(6):867. 10.3390/cells12060867. 36980208 10.3390/cells12060867 PMC10047059

[380] Hu H, Chen Y, Jing L, Zhai C, Shen L. The link between ferroptosis and cardiovascular diseases: a novel target for treatment. Front Cardiovasc Med. 2021;8:710963. 10.3389/fcvm.2021.710963. 34368260 10.3389/fcvm.2021.710963 PMC8341300

[381] Cicha I, Chauvierre C, Texier I, Cabella C, Metselaar JM, Szebeni J, et al. From design to the clinic: practical guidelines for translating cardiovascular nanomedicine. Cardiovasc Res. 2018;114(13):1714–27. 10.1093/cvr/cvy219. 30165574 10.1093/cvr/cvy219 PMC6198738

[382] Zhang L, Zhang Y, Zhao Y, Wang Y, Ding H, Xue S, et al. Circulating miRNAs as biomarkers for early diagnosis of coronary artery disease. Expert Opin Ther Pat. 2018;28(8):591–601. 10.1080/13543776.2018.1503650. 30064285 10.1080/13543776.2018.1503650

[383] Zhang H, Deng T, Liu R, Ning T, Yang H, Liu D, et al. CAF secreted miR-522 suppresses ferroptosis and promotes acquired chemo-resistance in gastric cancer. Mol Cancer. 2020;19(1):43. 10.1186/s12943-020-01168-8. 32106859 10.1186/s12943-020-01168-8 PMC7045485

[384] Fang X, Ardehali H, Min J, Wang F. The molecular and metabolic landscape of iron and ferroptosis in cardiovascular disease. Nat Rev Cardiol. 2023;20(1):7–23. 10.1038/s41569-022-00735-4. 35788564 10.1038/s41569-022-00735-4 PMC9252571

[385] Dhaliwal S, Kalogeropoulos AP. Markers of iron metabolism and outcomes in patients with heart failure: a systematic review. Int J Mol Sci. 2023;24(6):5645. 10.3390/ijms24065645. 36982717 10.3390/ijms24065645 PMC10059277

[386] Gan S, Azzo JD, Zhao L, Pourmussa B, Dib MJ, Salman O, et al. Transferrin saturation, serum iron, and ferritin in heart failure: prognostic significance and proteomic associations. Circ Heart Fail. 2025;18(2):e011728. 10.1161/CIRCHEARTFAILURE.124.011728. 39831311 10.1161/CIRCHEARTFAILURE.124.011728 PMC11835534

[387] Jankowska EA, Malyszko J, Ardehali H, Koc-Zorawska E, Banasiak W, von Haehling S, et al. Iron status in patients with chronic heart failure. Eur Heart J. 2013;34(11):827–34. 10.1093/eurheartj/ehs377. 23178646 10.1093/eurheartj/ehs377 PMC3697803

[388] Daiber A, Hahad O, Andreadou I, Steven S, Daub S, Münzel T. Redox-related biomarkers in human cardiovascular disease - classical footprints and beyond. Redox Biol. 2021;42:101875. 10.1016/j.redox.2021.101875. 33541847 10.1016/j.redox.2021.101875 PMC8113038

[389] Zhou D, Yang Y, Han R, He J, Liu D, Xia W, et al. Ferroptosis and its potential determinant role in myocardial susceptibility to ischemia/reperfusion injury in diabetes. Rev Cardiovasc Med. 2024;25(10):360. 10.31083/j.rcm2510360. 39484139 10.31083/j.rcm2510360 PMC11522832

[390] Kiyuna LA, Candido DS, Bechara LRG, Jesus ICG, Ramalho LS, Krum B, et al. 4-hydroxynonenal impairs miRNA maturation in heart failure via Dicer post-translational modification. Eur Heart J. 2023;44(44):4696–712. 10.1093/eurheartj/ehad662. 37944136 10.1093/eurheartj/ehad662 PMC13016698

[391] Pastori D, Andreozzi P, Carnevale R, Bartimoccia S, Limaj S, Melandri S, et al. Does the Coexistence of Chronic Obstructive Pulmonary Disease and Atrial Fibrillation Affect Nox2 Activity and Urinary Isoprostanes Excretion? Antioxid Redox Signal. 2019;31(11):786–90. 10.1089/ars.2019.7811. 31250672 10.1089/ars.2019.7811

[392] Davies SS, Roberts LJ. F2-isoprostanes as an indicator and risk factor for coronary heart disease. Free Radic Biol Med. 2011;50(5):559–66. 10.1016/j.freeradbiomed.2010.11.023. 21126576 10.1016/j.freeradbiomed.2010.11.023 PMC3058898

[393] Kalayinia S, Arjmand F, Maleki M, Malakootian M, Singh CP. MicroRNAs: roles in cardiovascular development and disease. Cardiovasc Pathol Off J Soc Cardiovasc Pathol. 2021;50:107296. 10.1016/j.carpath.2020.107296. 10.1016/j.carpath.2020.107296 33022373

[394] Li J, Wang N, Wen X, Huang LY, Cui RQ, Zhang J. Serum miRNA-203 as a novel biomarker for the early prediction of acute ST-elevation myocardial infarction. J Cardiovasc Transl Res. 2022;15(6):1406–13. 10.1007/s12265-022-10269-2. 35507256 10.1007/s12265-022-10269-2

[395] Xiao J, Gao R, Bei Y, Zhou Q, Zhou Y, Zhang H, et al. Circulating miR-30d predicts survival in patients with acute heart failure. Cell Physiol Biochem Int J Exp Cell Physiol Biochem Pharmacol. 2017;41(3):865–74. 10.1159/000459899. 10.1159/000459899 PMC5509048 28214846

[396] Yan L, Zhang Y, Zhang W, Deng SQ, Ge ZR. LncRNA-NRF is a potential biomarker of heart failure after acute myocardial infarction. J Cardiovasc Transl Res. 2020;13(6):1008–15. 10.1007/s12265-020-10029-0. 32440913 10.1007/s12265-020-10029-0 PMC7708339

[397] Zhu L, Feng Q, Fan J, Huang J, Zhu Y, Wu Y, et al. Clinical value of long non-coding RNA KCNQ1OT1 in estimating the stenosis, lipid level, inflammation status, and prognostication in coronary heart disease patients. J Clin Lab Anal. 2023;37(1):e24775. 10.1002/jcla.24775. 36458365 10.1002/jcla.24775 PMC9833965

[398] Sonnenschein K, Wilczek AL, de Gonzalo-Calvo D, Pfanne A, Derda AA, Zwadlo C, et al. Serum circular RNAs act as blood-based biomarkers for hypertrophic obstructive cardiomyopathy. Sci Rep. 2019;9(1):20350. 10.1038/s41598-019-56617-2. 31889077 10.1038/s41598-019-56617-2 PMC6937321

[399] Zhang J, Liu X, Li X, Cai Y, Zhou Y, Wang Q, et al. The emerging role of noncoding RNA regulation of the ferroptosis in cardiovascular diseases. Oxid Med Cell Longev. 2022;2022:3595745. 10.1155/2022/3595745. 36187333 10.1155/2022/3595745 PMC9519351

[400] Wu T, Shi G, Ji Z, Wang S, Geng L, Guo Z. Circulating small extracellular vesicle-encapsulated SEMA5A-IT1 attenuates myocardial ischemia-reperfusion injury after cardiac surgery with cardiopulmonary bypass. Cell Mol Biol Lett. 2022;27(1):95. 10.1186/s11658-022-00395-9. 36284269 10.1186/s11658-022-00395-9 PMC9594885

[401] Hou PP, Zheng CM, Wu SH, Liu XX, Xiang GX, Cai WY, et al. Extracellular vesicle-packaged ACSL4 induces hepatocyte senescence to promote hepatocellular carcinoma progression. Cancer Res. 2024;84(23):3953–66. 10.1158/0008-5472.CAN-24-0832. 39226516 10.1158/0008-5472.CAN-24-0832

[402] Núñez J, Miñana G, Cardells I, Palau P, Llàcer P, Fácila L, et al. Noninvasive imaging estimation of myocardial iron repletion following administration of intravenous iron: the Myocardial-IRON trial. J Am Heart Assoc. 2020;9(4):e014254. 10.1161/JAHA.119.014254. 32067585 10.1161/JAHA.119.014254 PMC7070181

[403] Ferré-Vallverdú M, Sánchez-Lacuesta E, Plaza-López D, Díez-Gil JL, Sepúlveda-Sanchis P, Gil-Cayuela C, et al. Prognostic value and clinical predictors of intramyocardial hemorrhage measured by CMR T2* sequences in STEMI. Int J Cardiovasc Imaging. 2021;37(5):1735–44. 10.1007/s10554-020-02142-7. 33442854 10.1007/s10554-020-02142-7

[404] Mota F, Pell VR, Singh N, Baark F, Waters E, Sadasivam P, et al. A Reactivity-Based 18F-Labeled Probe for PET Imaging of Oxidative Stress in Chemotherapy-Induced Cardiotoxicity. Mol Pharm. 2022;19(1):18–25. 10.1021/acs.molpharmaceut.1c00496. 34846906 10.1021/acs.molpharmaceut.1c00496 PMC8728736

[405] Felekkis K, Papaneophytou C. Challenges in using circulating micro-RNAs as biomarkers for cardiovascular diseases. Int J Mol Sci. 2020;21(2):561. 10.3390/ijms21020561. 31952319 10.3390/ijms21020561 PMC7013987

[406] Lawrence SR, Shah KM. Prospects and current challenges of extracellular vesicle-based biomarkers in cancer. Biology. 2024;13(9):694. 10.3390/biology13090694. 39336121 10.3390/biology13090694 PMC11428408

